# The causal relationship between sleep and risk of psychiatric disorders: A two-sample mendelian randomization study

**DOI:** 10.3389/fgene.2024.1380544

**Published:** 2024-06-17

**Authors:** Pei Chen, Jiuhang Qin, Yueying Wang, Jinjin Yuan, Yang Pan, Bingqian Zhu

**Affiliations:** ^1^ College of Nursing, University of Illinois Chicago, Chicago, IL, United States; ^2^ Department of Mathematics, Statistics, and Computer Science, University of Illinois Chicago, Chicago, IL, United States; ^3^ School of Nursing, Shanghai Jiao Tong University, Shanghai, China; ^4^ Division of Nephrology, Department of Medicine, College of Medicine, University of Illinois Chicago, Chicago, IL, United States

**Keywords:** sleep, psychiatric disorder, major depressive disorder, schizophrenia, attention-deficit/hyperactivity disorder, mendelian randomization, causal relationship

## Abstract

**Introduction:**

Sleep is associated with psychiatric disorders. However, their causality remains unknown.

**Methods:**

The study explored the causal relationship between seven sleep parameters (sleep duration, insomnia, sleep apnea, chronotype, daytime dozing, napping during the day, and snoring) and three psychiatric disorders including major depressive disorder (MDD), schizophrenia, and attention-deficit/hyperactivity disorder (ADHD) using two-sample Mendelian randomization (MR). Genome-wide association study (GWAS) summary data for sleep parameters were obtained from the United Kingdom biobank, FinnGen biobank, and EBI databases. MR-Egger, weighted median, inverse-variance weighted (IVW), simple mode, weighted mode, maximum likelihood, penalized weighted median, and IVW(fixed effects) were used to perform the MR analysis. The heterogeneity was detected by Cochran’s Q statistic. The horizontal pleiotropy was detected by MR Egger. The sensitivity was investigated by the leave-one-out analysis.

**Results:**

Insomnia (OR = 2.02, 95%CI = 1.34–3.03, *p* = 0.001, False-discovery rate (FDR) corrected *p*-value = 0.011) and napping during the day (OR = 1.81, 95%CI = 1.34–2.44, FDR corrected *p*-value<0.001) were associated with an increased risk of MDD. Longer sleep duration (OR = 2.20, 95%CI = 1.24–3.90, FDR corrected *p*-value = 0.049) had an association with the increased risk of schizophrenia, while daytime dozing (OR = 4.44, 95%CI = 1.20–16.41, corrected *p*-value = 0.088)and napping during the day (OR = 2.11, 95%CI = 1.11–4.02, FDR corrected *p*-value = 0.088) had a suggestive association with an increased risk of schizophrenia. Longer sleep duration had a suggestive association with a decreased risk of ADHD (OR = 0.66, 95%CI = 0.42–0.93, FDR corrected *p*-value = 0.088).

**Conclusion:**

This study provides further evidence for a complex relationship between sleep and psychiatric disorders. Our findings highlight the potential benefits of addressing sleep problems in the prevention of psychiatric disorders.

## 1 Introduction

Psychiatric disorders represent a significant public health concern, as they are implicated in approximately 70% of suicides (Baldessarini and Tondo, 2020; Pooja et al., 2021). Among these, major depressive disorder (MDD) has been the most common psychiatric disorder, affecting around 163 million people worldwide (Hashmi et al., 2022). Meanwhile, 73 million people were affected by attention-deficit/hyperactivity disorder (ADHD) and 19 million people suffered from schizophrenia (GBD, 2017 Disease and Injury Incidence and Prevalence Collaborators, 2018), bringing huge burdens. Psychiatric disorders, prominently MDD, ADHD, and schizophrenia, impose significant public health challenges, affecting millions globally. The profound impacts of these conditions on both personal and societal levels underscore the critical need for identifying modifiable risk factors that can be targeted through therapeutic interventions (Murray et al., 2012).

Psychiatric disorders are closely associated with sleep (Sutton, 2014), including individual sleep dimensions (e.g., sleep duration, sleep quality, and sleep timing) and sleep disturbances (e.g., insomnia, sleep apnea). On the one hand, sleep disruptions (e.g., insomnia or excessive sleep) are the defining features and diagnostic criteria of depression (Battle, 2013). Based on an epidemiological study conducted in the US, among individuals with depression, 92% reported at least one type of sleep-related complaint, 85% experienced insomnia, and 48% experienced hypersomnia (Geoffroy et al., 2018). On the other hand, sleep disturbances may serve as potential risk factors for the onset of psychiatric disorders. According to a prospective cohort study, insomnia symptoms were predictors of depression, (Dong and Yang, 2019). Therapeutic interventions targeting sleep disturbances (e.g., insomnia and sleep apnea) alleviated symptoms related to depression (Hertenstein et al., 2022), schizophrenia (Mylonas et al., 2020), and ADHD (Liu et al., 2023). Given these insights, our study has meticulously selected seven sleep parameters: sleep duration, insomnia, sleep apnea, chronotype, daytime dozing, napping during the day, and snoring. These parameters were specifically chosen for their documented impacts on psychiatric health, as evidenced by their diagnostic relevance and frequent alteration in psychiatric patients, supported by both clinical observations and epidemiological studies. This comprehensive examination of selected sleep parameters across major depressive disorder, schizophrenia, and ADHD not only aims to elucidate the causal pathways involved but also seeks to identify potential therapeutic interventions that could significantly improve outcomes for patients suffering from these debilitating disorders.

Mendelian randomization (MR) is an epidemiological method that uses genetic variants as instrumental variables to investigate the causal association between exposures and outcomes. MR could be considered conceptually as natural randomized controlled trial studies (RCTs) because genetic variants are randomly inherited (Emdin et al., 2017). MR is an alternative approach to explore the potential causal relationship between an exposure and an outcome when RCTs are not feasible due to practical reasons (e.g., expensive financial investment and ethical issues). In addition, MR studies can overcome limitations of observational studies (e.g., inability to control for potential confounding factors), and thus provide stronger evidence of causation. For instance, certain genetic variants have been associated with sleep characteristics (e.g., sleep duration, propensity for insomnia, and circadian rhythms) (Sun et al., 2022). These genetic variants are fixed at birth and are rarely influenced by acquired environment and behaviors. Therefore, by analyzing associations between the underlying genetic variants of sleep characteristics with psychiatric disorders, causality can be reliably inferred.

Recent advances in the field of MR have significantly contributed to our understanding of the intricate causal relationships between various sleep traits and psychiatric disorders. A pivotal bidirectional Mendelian Randomization (MR) study meticulously examined the complex interactions between crucial sleep characteristics, including insomnia, chronotype, and sleep duration, and a spectrum of psychiatric disorders such as MDD, ADHD, Post-Traumatic Stress Disorder (PTSD), Bipolar Disorder (BD), and Schizophrenia (Sun et al., 2022). The findings of this study revealed distinct associations: insomnia was linked to MDD, ADHD, and PTSD; chronotype showed associations with MDD and Schizophrenia; and sleep duration was correlated with bipolar disorder. These associations underscore the multifaceted impact of sleep patterns on mental health, highlighting specific sleep-related factors contributing to the etiology of diverse psychiatric conditions. A seminal study by Huang et al. is particularly illuminating in this regard, revealing a robust association between insomnia and increased susceptibility to ADHD and MDD (Huang et al., 2021). This finding is corroborated by subsequent research, which further underscores the link between insomnia and an elevated risk of MDD development. Another study adds another layer of complexity, suggesting a protective effect of morning diurnal preference against schizophrenia (Wang et al., 2022). In this study, longer sleep durations and habitual daytime napping are implicated in an increased risk of developing schizophrenia. Moreover, other MR investigations have shed light on the broader implications of diurnal preferences, linking late diurnal preference to poorer mental health outcomes (O’Loughlin et al., 2021). A particularly targeted study provides compelling evidence that, among the various sleep disturbances, insomnia specifically, as opposed to short or long sleep durations, exerts a causal influence on anxiety disorders (Huang et al., 2020). Those studies primarily focused on a specific sleep parameter (e.g., insomnia or sleep duration) or targeted individual psychiatric disorders (e.g., schizophrenia). This selective focus, despite its contributions, has resulted in a narrow scope of evidence. There is a need to include a wide range of sleep parameters and examine their effects on common psychiatric disorders to address existing gaps and enhance our understanding of these complex relationships. Such an inclusive and expansive approach is essential to deeper address the existing gaps in the literature and to deepen our holistic understanding of the complex interplay between sleep traits and psychiatric health.

In the present study, we employed a two-sample MR approach to systematically explore the causal relationship between a comprehensive set of sleep parameters—sleep duration, insomnia, sleep apnea, chronotype, daytime dozing, napping during the day, and snoring—and three major psychiatric disorders: MDD, schizophrenia, and ADHD. This method offers a robust alternative to traditional observational studies by mitigating issues related to confounding factors and reverse causality. Our investigation aims to enhance our understanding of the etiology of these psychiatric disorders and could lead to the development of novel preventative strategies.

## 2 Materials and methods

### 2.1 Study design

A two-sample MR design was selected to investigate the causal associations of sleep (sleep duration, insomnia, sleep apnea, chronotype, daytime dozing, napping during the day, and snoring) with psychiatric disorders including MDD, schizophrenia, and ADHD. Sleep was the exposure and psychiatric disorders were the outcomes. We thus performed a total of 21 MR analyses.

Instrumental variables (IV) for both sleep and psychiatric disorders were based on the following three key assumptions (Woolf et al., 2022): (a) a relevance assumption--the genetic variants should be strongly associated with the exposure; (b) an independence assumption--the genetic variants should be independent of potential confounders; and (c) an exclusion restriction--the genetic variants should be associated with the outcome only through the exposure.

### 2.2 Selection of genetic instruments for MR analyses

All individuals included in the genome-wide association study (GWAS) were of European ethnic origin (Table 1). For each exposure factor, the single-nucleotide polymorphisms (SNPs) were filtered according to the three main MR assumptions. To satisfy the “relevance assumption”, SNPs that achieved p < 5 × 10^−8^ were selected. To meet the “independence assumption”, we searched the curated SNP-to-phenotype databases of PhenoScanner (Version 2; http://www.phenoscanner.medschl.cam.ac.uk/) for other significant (p < 5 × 10^−8^) genome-wide phenotypes related to the chosen SNPs and their correlated SNPs, which may affect the outcomes via paths other than the exposures of interest (Kamat et al., 2019). Additionally, we performed linkage disequilibrium (LD) calculation across SNPs to select independent SNPs based on LD *r* ^2^ < 0.001 and clump distance >10,000 kb (Purcell et al., 2007). We used these carefully chosen SNPs as the final genetic IVs for the subsequent MR analysis.

**TABLE 1:** GWAS for sleep parameters.

Author, year	GWAS ID	Trait	Sample size	nSNP	Consortium
Dashti, 2019 (Dashti et al., 2019)	ukb-b-4424	Sleep duration	460,099	9,851,867	United Kingdom Biobank
Lane, 2019 (Lane et al., 2019)	ukb-b-3957	Insomnia	462,341	9,851,867	United Kingdom Biobank
Strausz, 2021 (Strausz et al., 2021)	finn-b-G6_SLEEPAPNO_INCLAVO	Sleep apnea	218,792	16,380,466	FinGen biobank
Jones, 2019 (Jones et al., 2019)	ukb-b-4956	Chronotype	413,343	9,851,867	United Kingdom Biobank
Wang, 2019 (Wang et al., 2019)	ukb-b-5776	Daytime dozing	460,913	9,851,867	United Kingdom Biobank
Wang, 2019 (Wang et al., 2019)	ukb-b-4616	Napping during the day	462,400	9,851,867	United Kingdom Biobank
Campos, 2020 (Campos et al., 2020)	ebi-a-GCST009760	Snoring	408,317	10,707,662	EBI database

Abbreviations: GWAS, genome‐wide association study; GWAS ID, GWAS, identity; nSNP, the number of single‐nucleotide polymorphism.

### 2.3 Data sources and SNP selection for sleep parameters

For sleep duration, insomnia, chronotype, daytime dozing, and napping during the day, we used the largest published GWAS, which had been conducted in a population of European descent (Elsworth et al., 2020). These data were from an MRC Integrative Epidemiology Unit (IEU) at the University of Bristol analysis of United Kingdom Biobank phenotypes (UK Biobank, 2017). The United Kingdom Biobank is a comprehensive database, encompassing data from approximately 500,000 volunteers, who were aged between 40 and 69 years at the time of recruitment. This database includes not only genetic data and physiological measurements but also detailed health records and information about the lifestyle and environment. SNPs were selected to serve as instrumental variables for various sleep parameters. It included 71 SNPs associated with sleep duration (refer to Supplementary Table S1), 42 SNPs linked to insomnia (see Supplementary Table S2), and 161 SNPs related to chronotype (outlined in Supplementary Table S3). Additionally, 31 SNPs were chosen for their relevance to daytime dozing (detailed in Supplementary Table S4), while 94 SNPs were selected for their association with napping during the day (listed in Supplementary Table S5).

Sleep duration was measured by a standardized question “About how many hours of sleep do you get in every 24 h? (please include naps)” with responses in integer hours. Two binary variables were derived from self-reported sleep duration, i.e., short sleep (<7 h, the number of cases was 106,192) and long sleep (<7 h, the number of cases was 34,184) (Cappuccio et al., 2010). Insomnia was assessed by the question “do you have trouble falling asleep at night or do you wake up in the middle of the night?”, with 129,720 insomnia patients and 237,627 controls. Chronotype was measured by a question “Do you consider yourself to be definitely a morning person/more a morning than evening/more an evening than morning/definitely an evening person/do not know/prefer not to answer”, identifying 252,287 as morning person and 150,908 as evening person. Daytime dozing was assessed by a question “How likely are you to doze off or fall asleep during the daytime when you do not mean to? E.g., when working, reading or driving”, resulting in 104,786 daytime dozing and 356,127 controls. Napping during the day was measured by a question “Do you have a nap during the day” while the numbers of cases was not avaliable.

For sleep apnea, we used the largest number of SNPs of GWAS which was conducted in a population of European descent. These data were from FinnGen biobank, which included data for 16,761 sleep apnea patients and 201,194 controls. Sleep apnea were defined using participant-reported diagnosis or ICD diagnostic codes available in electronic health records (ICD-9: 327.23 and ICD-10: G47.3) (Campos et al., 2023). FinnGen is a large biobank study that aims to genotype 500,0000 Finns, which included data for including prospective and retrospective epidemiological and disease-based cohorts as well as hospital biobank samples. In total, five SNPs that showed genome-wide significance were selected as instrumental variables (Supplementary Table S6).

For snoring, we used the largest and most recently published GWAS which was conducted in a population of European descent, including 152,302 snoring cases and 256,015 controls. Snoring was measured by a question “Does your partner or a close relative or friend complain about your snoring”. These data were from the European Bioinformatics Institute (EBI) databases which is a hub for research and services in bioinformatics (Cook et al., 2020; Cantelli et al., 2022). In total, 38 SNPs that showed genome-wide significance were selected as instrumental variables for snoring (Supplementary Table S7).

### 2.4 Statistical analysis

**FIGURE 1:**
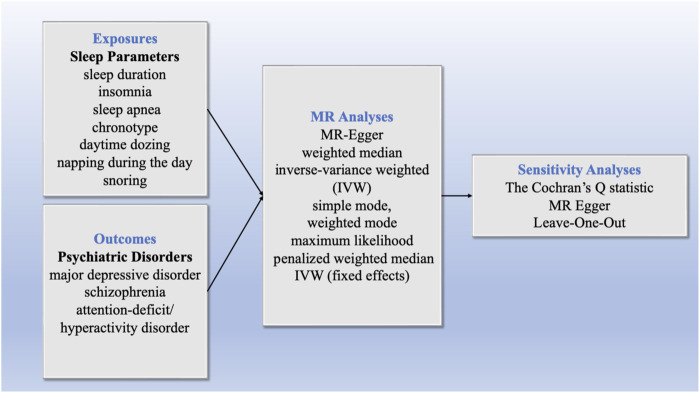
Flowchart of MR analysis for sleep parameters and psychiatric disorders.

We performed the statistical analyses using R version 3.6.3 (R Foundation for Statistical Computing, Vienna, Austria) and R package TwoSampleMR. We compared eight different MR methods: MR-Egger, weighted median, inverse-variance weighted (IVW), simple mode, weighted mode, maximum likelihood, penalized weighted median, and inverse-variance weighted (fixed effects). All these methods are based on similar assumptions about the nature of pleiotropy (Boef et al., 2015). We conducted the primary analysis using the IVW method with multiplicative random effects. This method is optimal for two-sample MR, provided that SNPs strictly adhere to essential assumptions (Burgess et al., 2019). The differences between these 8 MR methods (Sanderson et al., 2022) were shown in Table 2. The Cochran’s Q statistic (IVW) and Rucker’s Q statistic (MR Egger) were used to detect the heterogeneity of our MR analysis, and *p*-values> 0.05 indicated no heterogeneity (Hemani et al., 2018). The intercept test of MR Egger was used to detect the horizontal pleiotropy, and *p*-values>0.05 indicated no horizontal pleiotropy (Shu et al., 2022). The leave-one-out analysis was used to investigate whether the causal relationship between sleep and psychiatric disorders was influenced by a single SNP (Lee, 2019). The main parameters included sample size, type I error rate, proportion of cases, odds ratio (OR) of the outcome, and proportion of the variance explained for the association between the SNPs and the exposure variable (*r* ^2^). The statistical significance was set at a two-sided *p*-values<0.05. To avoid the inflation of false-positive findings, we calculated the false-discovery rate (FDR) corrected *p*-values for the main analyses (Korthauer et al., 2019). Significance was determined as FDR-corrected *p*-values<0.05, whereas *p*-values <0.05 that did not meet the FDR-corrected threshold were regarded as suggestive evidence of an association. The overview of the study design, illustrating the various sleep parameters and psychiatric disorders analyzed, the MR methods employed, and the sensitivity analyses conducted was shown in Figure 1.

**TABLE 2:** Comparison of mendelian randomization methods.

	Strengths	Limitations
MR-Egger (Burgess and Thompson, 2017)	Provides unbiased estimates under some pleiotropic conditions	Less precise, potentially higher standard errors; affected by weaker instruments
Weighted Median (Bowden et al., 2016)	Offers compromise between robustness and efficiency	Requires larger number of valid instruments for reliable estimation
IVW (Boef et al., 2015)	Efficient under ideal conditions	Biased under pleiotropy; assumes homogeneity in absence of random effects
Simple Mode (Bowden et al., 2016)	Computationally simple and fast	Susceptible to biases from invalid instruments and pleiotropy
Weighted Mode (Bowden et al., 2016)	Reduces impact of outlier instrumental variables	Complexity in determining optimal weights
Maximum Likelihood (Jiang et al., 2022)	Utilizes all available information, potentially increasing power	Computationally intensive; requires strong assumptions about model form
Penalized Weighted Median (Slob and Burgess, 2020)	Enhances stability and robustness	Selection of appropriate penalty parameters can be challenging
IVW (Fixed Effects) (Xu et al., 2022)	Simple and efficient with homogeneous instruments	Poor performance if homogeneity assumption is violated

### 2.5 Ethics approval and consent to participate

All cited genome-wide association studies, epigenome-wide association studies and summary-level data had been approved by a relevant review board.

## 3 Results

In this study, a total of 21 MR analyses were conducted. These analyses encompassed a range of MR methodologies, including but not limited to MR-Egger, Weighted Median, and IVW. Each method was rigorously applied and evaluated to explore various aspects of the genetic associations under investigation. We used the IVW method as the main method for all MR analyses. The IVW model, which generates a stable causal inference even in the presence of horizontal pleiotropy, provided a weighted regression of the IV-specific causal estimation. The results of IVW were shown in Table 3. To maintain clarity and conciseness in the presentation of our findings, the detailed outcomes of all MR analyses have been compiled in the (Supplementary Table S8) accompanying this manuscript.

**TABLE 3:** Summary of IVW results for the association between sleep parameters and psychiatric disorders.

Exposure	Outcome	OR	95%CI lower	95%CI upper	*p*	FDR adjusted_*p*
sleep duration	major depressive disorder	0.855	0.627	1.166	0.322	0.564
insomnia	major depressive disorder	2.017	1.344	3.026	0.001	0.011
sleep apnea	major depressive disorder	1.038	0.863	1.249	0.692	0.855
chronotype	major depressive disorder	1.079	0.949	1.228	0.247	0.564
daytime dozing	major depressive disorder	1.185	0.683	2.054	0.546	0.835
napping during the day	major depressive disorder	1.807	1.336	2.443	<0.001	<0.001
snoring	major depressive disorder	0.836	0.428	1.632	0.599	0.835
sleep duration	schizophrenia	2.2	1.241	3.901	0.007	0.049
insomnia	schizophrenia	1.006	0.454	2.231	0.988	0.988
sleep apnea	schizophrenia	1.015	0.865	1.191	0.855	0.908
chronotype	schizophrenia	1.233	0.943	1.613	0.125	0.328
daytime dozing	schizophrenia	4.439	1.201	16.407	0.025	0.088
napping during the day	schizophrenia	2.111	1.109	4.018	0.023	0.088
snoring	schizophrenia	0.514	0.146	1.813	0.301	0.564
sleep duration	ADHD	0.625	0.417	0.934	0.022	0.088
insomnia	ADHD	1.43	0.729	2.804	0.298	0.564
sleep apnea	ADHD	1.015	0.854	1.206	0.865	0.908
chronotype	ADHD	0.936	0.723	1.212	0.617	0.835
daytime dozing	ADHD	0.896	0.3	2.675	0.844	0.908
napping during the day	ADHD	1.455	0.901	2.35	0.125	0.328
snoring	ADHD	1.265	0.479	3.338	0.636	0.835

Note: ADHD, attention-deficit/hyperactivity disorder. CI, confidence interval; OR, odds ratio.

**FIGURE 2:**
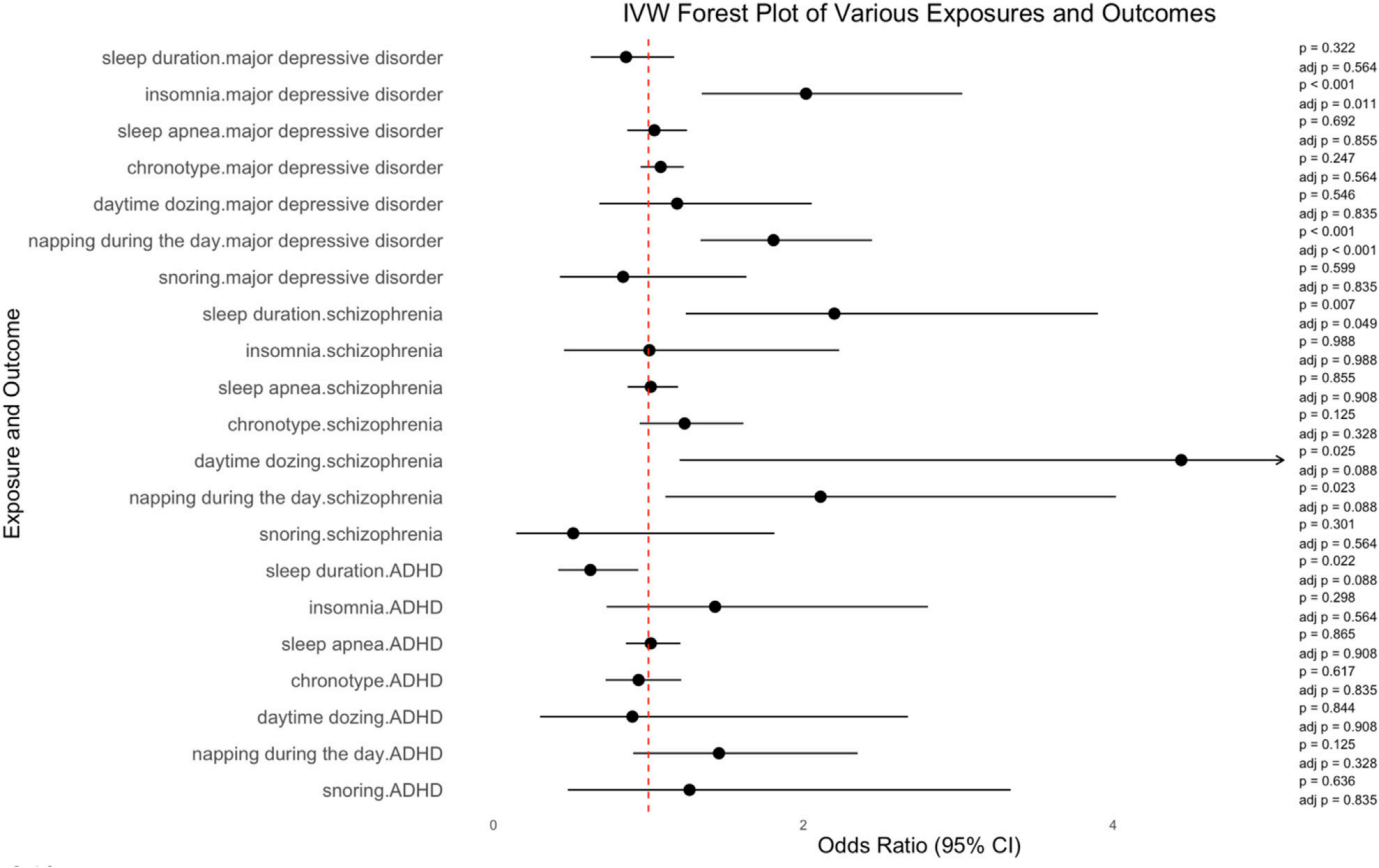
IVW results of the causal relationship between sleep and risk of psychiatric disorders.

Insomnia was significantly associated with an increased risk of MDD, according to the results of IVW (number of SNPs = 32, OR = 2.017, 95%CI = 1.344–3.026, SE = 0.207, corrected *p*-value = 0.011). Napping during the day also showed a significant association with an increased risk of MDD, according to the results or IVW (number of SNPs = 83, OR = 1.807, 95%CI = 1.336–2.443, SE = 0.154, corrected *p*-value<0.001). The increased risk of schizophrenia was linked to sleep duration (number of SNPs = 57, OR = 2.200, 95%CI = 1.241–3.901, SE = 0.292, corrected *p*-value = 0.049). Daytime dozing was associated with schizophrenia, but the association was not statistically significant (number of SNPs = 23, OR = 4.439, 95%CI = 1.201–16.407, SE = 0.667, corrected *p*-value = 0.088). Similarly, the association between napping during the day and schizophrenia was not significant (number of SNPs = 82, OR = 2.111, 95%CI = 1.109–4.018, SE = 0.328, corrected *p*-value = 0.088). Neither was the association between sleep duration and ADHD (number of SNPs = 52, OR = 0.625, 95%CI = 0.417–0.934, SE = 0.206, corrected *p*-value = 0.088). IVW results of the causal relationship between sleep parameters and the risk of psychiatric disorders are shown in Figure 2.

No causal association was found of sleep duration (number of SNPs = 56, OR = 0.855, 95%CI = 0.627, 1.166, SE = 0.158, corrected *p*-value = 0.564), sleep apnea (number of SNPs = 5, OR = 1.038, 95%CI = 0.863, 1.249, SE = 0.094, corrected *p*-value = 0.855), chronotype (number of SNPs = 133, OR = 1.079, 95%CI = 0.949–1.228, SE = 0.066, corrected *p*-value = 0.564), day time dozing (number of SNPs = 28, OR = 1.185, 95%CI = 0.683–2.054, SE = 0.281, corrected *p*-value = 0.835), and snoring (number of SNPs = 23, OR = 0.836, 95%CI = 0.428–1.632, SE = 0.341, corrected *p*-value = 0.835) with MDD. Additionally, there was no causal relationship of insomnia (number of SNPs = 34, OR = 1.006, 95%CI = 0.454–2.231, SE = 0.406, corrected *p*-value = 0.988), sleep apnea (number of SNPs = 5, OR = 1.015, 95%CI = 0.865–1.191, SE = 0.081, corrected *p*-value = 0.908), chronotype (number of SNPs = 142, OR = 1.233, 95%CI = 0.943–1.613, SE = 0.137, corrected *p*-value = 0.328), and snoring (number of SNPs = 23, OR = 0.514, 95%CI = 0.146–1.813, SE = 0.643, corrected *p*-value = 0.564) with schizophrenia. There was no causal relationship of insomnia (number of SNPs = 31, OR = 1.430, 95%CI = 0.729–2.804, SE = 0.344, corrected *p*-value = 0.564), sleep apnea (number of SNPs = 4, OR = 1.015, 95%CI = 0.854–1.206, SE = 0.088, corrected *p*-value = 0.908), chronotype (number of SNPs = 129, OR = 0.936, 95%CI = 0.723–1.212, SE = 0.132, corrected *p*-value = 0.835), day time dozing (number of SNPs = 30, OR = 0.896, 95%CI = 0.300–2.675, SE = 0.558, corrected *p*-value = 0.908), napping during the day (number of SNPs = 78, OR = 1.455, 95%CI = 0.901–2.350, SE = 0.245, corrected *p*-value = 0.328), and snoring (number of SNPs = 29, OR = 1.265, 95%CI = 0.479–3.338, SE = 0.495, corrected *p*-value = 0.835) with ADHD. More details are shown in Supplementary Table S8 and Supplementary Figure S1–S21.

Sensitivity analyses revealed the robustness of the findings (Supplementary Table S9; Supplementary Figure S22–S63). Heterogeneity was found only in the test of sleep duration and ADHD. There was no heterogeneity among other tests. Horizontal pleiotropy was unlikely to skew the causality of sleep-related factors with psychiatric disorders according to the MR-Egger regression (Supplementary Table S10). Leave-one-out analysis indicated that the causal estimates of sleep and psychiatric disorders were not driven by any single SNP (Supplementary Figure S64–S84).

## 4 Discussion

In this MR study, we explored the causal associations between seven sleep parameters and three common psychiatric disorders. Our findings indicate that insomnia and napping during the day were significantly associated with increased MDD risk. Additionally, sleep duration, daytime dozing, and napping during the day showed an effect on increasing the risk of schizophrenia, while sleep duration showed an effect on decreasing the risk of ADHD.

Epidemiological evidence has revealed associations between insomnia, daytime dozing, and napping during the day. However, the extent to which the underlying genetics are shared is unknown (Lane et al., 2017). Our results confirmed daytime dozing and napping during the day shared the same SNP rs12140153 and rs13284688 which could be considered evidence for underlying genetics. The mutations in SNP rs12140153 have a profound effect on the protein Pals1-associated tight junction (PATJ). The PATJ gene encodes a membrane-associated protein that plays a crucial role in the formation of tight junctions, essential for maintaining the integrity of various cellular structures and functions. Research has implicated this protein in photoreception in model organisms such as mice and *Drosophila*, suggesting that it may play a role in sleep-wake cycle regulation via the entrainment of circadian rhythms to light-dark cycles (Peirson et al., 2007). The exact mechanism through which mutations in rs12140513 influence PATJ function and, in turn, affect sleep patterns warrants further investigation.

Our findings indicate that insomnia and napping during the day are significantly associated with an increased MDD risk. This finding consistent with findings from recent studies (Baranova et al., 2022; Sun et al., 2022; Yao et al., 2022). The potential underlying mechanisms are likely due to neurotransmitter imbalance, such as enhanced cholinergic or diminished aminergic neurotransmissions, brain activation abnormalities in emotion regulation areas, dysregulation of the hypothalamic–pituitary–adrenal axis, and increased inflammation (Freeman et al., 2020). Chronic insomnia may cause overactivity in brain regions responsible for mood regulation, leading to more frequent mood swings and contributing to the low mood characteristic of depression. Furthermore, insomnia might reduce brain’s sensitivity to neurotransmitters like serotonin. A reduction in serotonin receptor sensitivity and serotonin neurotransmission has often been implicated in the pathophysiology of major depressive disorder (Meerlo et al., 2008). Interventions targeting sleep have showed an effect on reducing depressive symptoms, underscoring the connection between sleep and mood (Gee et al., 2019).

This link is crucial for understanding the broader implications of sleep disorders on mental health and underscores the importance of addressing sleep disturbances within mental health treatment paradigms (Baranova et al., 2022). In addition to psychiatric disorders, insomnia has demonstrated associations with systemic diseases such as cardiovascular disease, as discussed in recent studies (Cao et al., 2024). This connection suggests that the scope of insomnia’s impact extends well beyond mental health, influencing overall physical health and potentially complicating treatment approaches for related physical ailments. Daytime napping may disrupt normal circadian rhythms, affecting hormone production and the body clock, and is associated with symptoms of depression (e.g., a lack of energy and low mood). While a moderate amount of napping may help reduced sleep pressure and restore energy, too much may lead to decreased quality of sleep at night, further exacerbating depressive symptoms (Crouse et al., 2021). Currently, evidence about the relationship between napping and MDD is scarce. In the future, more studies using a longitudinal or interventional design are needed to confirm their association.

Our study showed that sleep duration was associated with an increased risk for schizophrenia, while daytime dozing and napping during the day had a suggestive association with the risk of schizophrenia, consistent with Wang et al. study (Wang et al., 2022). The underlying mechanisms that link sleep with schizophrenia are complex and multifaceted and may involve disturbances in the sleep structure and circadian rhythm system in patients with schizophrenia (Waite et al., 2020). When sleep deprivation occurs, it can cause an increase in dopamine release in the brain. This increase is particularly significant in individuals with schizophrenia, as it can intensify positive symptoms (Ferrarelli, 2021). Moreover, slow-wave sleep (or deep sleep) plays a pivotal role in cognitive processing and emotional stabilization (Walker, 2009). A lack of sleep can result in a decrease in the proportion of slow-wave sleep, which may affect cognitive processing, making individuals more susceptible to hallucinations and delusions (Walker, 2009). Our study used Mendelian randomization to establish a causal link between sleep and schizophrenia, adding to the current evidence base.

Our study also revealed that sleep duration had a suggestive association with the risk of ADHD. The crucial role of sleep in promoting the optimal development and organization of neural circuitry is well-known (Lunsford-Avery et al., 2016). Both insufficient and excessive sleep can disrupt the equilibrium of neurotransmitters in the brain, particularly those involved in regulating attention, behavior, and emotions, such as dopamine (Tononi and Cirelli, 2012). This disruption could elevate the risk of developing ADHD. In addition, the quality of sleep, often compromised by either too little or too much sleep, may result in daytime fatigue, decreased attention span, and emotional instability (Tononi and Cirelli, 2012). These findings provide an important direction for future research about the relationship between sleep and ADHD.

A major strength of this study was the MR design, which minimized the residual confounding and reverse causality inherent in observational studies and allowed us to interrogate the potential causality between sleep and common psychiatric disorders. Additionally, we included a variety of sleep parameters and psychiatric disorders. Sensitivity analyses supported the validity of the estimates. The IVs used in this study were extracted from the largest GWAS of sleep with the largest sample sizes and were strongly associated with the exposure of interest. This approach minimized weak-instrument bias and increased statistical power in our analyses. However, our study also has limitations. First, the study’s reliance on genetic instruments from GWAS summary data may not fully capture the multifaceted nature of sleep parameters and psychiatric disorders. Genetic variants used as instruments may not comprehensively represent the biological mechanisms underlying these conditions. Second, although horizontal pleiotropy was assessed using MR-Egger, the potential for unrecognized pleiotropic effects remains a concern. MR assumptions such as the instrument’s exclusive effect through the exposure (exclusion restriction) and the absence of unmeasured confounders between the instrument and the outcome are critical for unbiased causal inference. Any violations of these assumptions can lead to biased estimates, limiting the conclusiveness of our findings. Moreover, the detected heterogeneity, as indicated by Cochran’s Q statistic, suggests variability in the effects across different genetic instruments, which could further complicate the causal interpretation. Third, the GWAS summary data derived from the United Kingdom Biobank, FinnGen Biobank, and EBI databases may limit the generalizability of the findings. These populations may not fully represent the global diversity, particularly in terms of ethnic and genetic backgrounds, which could influence the sleep-psychiatry relationship. Lastly, while this study focused on three specific psychiatric disorders (MDD, schizophrenia, and ADHD), the causal relationship between sleep parameters and other psychiatric conditions remains to be explored. This suggests a need for broader investigation into how sleep disturbances may influence a wider range of psychiatric conditions, thereby extending the relevance of our findings to other disorders and potentially uncovering novel therapeutic targets or preventative strategies.

## 5 Conclusion

Our study found a causal association between insomnia and napping during the day with the risk of MDD. Additionally, sleep duration was associated with an increased risk for schizophrenia, while daytime dozing and napping during the day had a suggestive association with the risk of schizophrenia. Furthermore, our findings also revealed that sleep duration had a suggestive association with the risk of ADHD.

## Data Availability

The original contributions presented in the study are included in the article/Supplementary Material, further inquiries can be directed to the corresponding author.
