# Supplementary material for: The causal relationship between sleep and risk of psychiatric disorders: A two-sample mendelian randomization study
Source: Front Genet. 2024 Jun 17;15:1380544. doi: 10.3389/fgene.2024.1380544 (PMC11215123; doi:10.3389/fgene.2024.1380544)
Supplement: Supplementary file 1 [file Table1.DOCX]

Supplementary Material

# Supplementary tables

Table S1. Sleep Duration Genetic IVs

| ID | SNP | EA | NEA | EAF | β | SE | chr | P-value | mr_keep.outcome |
| --- | --- | --- | --- | --- | --- | --- | --- | --- | --- |
| ukb-b-4424 | rs915416 | G | C | 0.70906 | -0.01273 | 0.00176031 | 1 | 4.79954E-13 | TRUE |
| ukb-b-4424 | rs2186122 | T | A | 0.559911 | -0.01083 | 0.00162062 | 1 | 2.29985E-11 | TRUE |
| ukb-b-4424 | rs2279681 | G | C | 0.341785 | 0.009299 | 0.00168475 | 1 | 3.40001E-08 | TRUE |
| ukb-b-4424 | rs7517981 | C | T | 0.601498 | -0.00997 | 0.00163399 | 1 | 1.09999E-09 | TRUE |
| ukb-b-4424 | rs12567114 | A | G | 0.276368 | 0.012338 | 0.00179427 | 1 | 6.09958E-12 | TRUE |
| ukb-b-4424 | rs1463053 | A | G | 0.639694 | 0.00927 | 0.00166133 | 1 | 2.39999E-08 | TRUE |
| ukb-b-4424 | rs6681755 | A | G | 0.199783 | 0.011527 | 0.00200498 | 1 | 8.99995E-09 | TRUE |
| ukb-b-4424 | rs374153 | T | C | 0.842567 | -0.0131 | 0.00219716 | 2 | 2.5E-09 | TRUE |
| ukb-b-4424 | rs2863957 | A | C | 0.220537 | 0.028904 | 0.00192922 | 2 | 9.60064E-51 | TRUE |
| ukb-b-4424 | rs1972712 | C | T | 0.249455 | 0.011796 | 0.00184758 | 2 | 1.7E-10 | TRUE |
| ukb-b-4424 | rs72831782 | A | T | 0.269409 | -0.01018 | 0.00184372 | 2 | 3.40001E-08 | TRUE |
| ukb-b-4424 | rs2683630 | G | C | 0.629076 | 0.014951 | 0.0016555 | 2 | 1.69981E-19 | TRUE |
| ukb-b-4424 | rs75539574 | C | A | 0.085774 | 0.023665 | 0.00287424 | 2 | 1.80011E-16 | TRUE |
| ukb-b-4424 | rs35662245 | A | T | 0.338713 | 0.010157 | 0.00169075 | 2 | 1.89998E-09 | TRUE |
| ukb-b-4424 | rs6783516 | T | G | 0.583817 | -0.00984 | 0.00163116 | 3 | 1.6E-09 | TRUE |
| ukb-b-4424 | rs76258078 | G | A | 0.049994 | -0.02169 | 0.00368137 | 3 | 3.79997E-09 | TRUE |
| ukb-b-4424 | rs113021516 | C | G | 0.335898 | 0.011481 | 0.00169722 | 3 | 1.29987E-11 | TRUE |
| ukb-b-4424 | rs17732997 | G | C | 0.429679 | -0.00884 | 0.00161761 | 3 | 4.60002E-08 | TRUE |
| ukb-b-4424 | rs9810474 | T | C | 0.232184 | -0.01115 | 0.00189404 | 3 | 3.89996E-09 | TRUE |
| ukb-b-4424 | rs7644809 | C | T | 0.576033 | -0.01015 | 0.00162466 | 3 | 4.09996E-10 | TRUE |
| ukb-b-4424 | rs13107325 | T | C | 0.074905 | -0.02427 | 0.00303933 | 4 | 1.39991E-15 | TRUE |
| ukb-b-4424 | rs2192528 | G | A | 0.522493 | -0.00981 | 0.00160143 | 4 | 9.09997E-10 | TRUE |
| ukb-b-4424 | rs2839753 | C | T | 0.265347 | -0.01064 | 0.00181248 | 4 | 4.39997E-09 | TRUE |
| ukb-b-4424 | rs7711696 | T | G | 0.304987 | -0.00987 | 0.00173522 | 5 | 1.29999E-08 | TRUE |
| ukb-b-4424 | rs12518468 | C | T | 0.328685 | -0.01064 | 0.00170288 | 5 | 4.09996E-10 | TRUE |
| ukb-b-4424 | rs365663 | G | A | 0.45495 | -0.00928 | 0.00161004 | 5 | 8.10009E-09 | TRUE |
| ukb-b-4424 | rs6889592 | A | G | 0.332608 | 0.011766 | 0.00169707 | 5 | 4.10015E-12 | TRUE |
| ukb-b-4424 | rs151014368 | A | G | 0.20734 | 0.011379 | 0.00198822 | 5 | 0.00000001 | TRUE |
| ukb-b-4424 | rs9382445 | C | T | 0.375168 | -0.00948 | 0.00164901 | 6 | 8.9E-09 | TRUE |
| ukb-b-4424 | rs1611719 | A | G | 0.209698 | -0.01321 | 0.00202652 | 6 | 7.10068E-11 | TRUE |
| ukb-b-4424 | rs113113059 | C | T | 0.219834 | -0.01113 | 0.00193332 | 6 | 8.60003E-09 | TRUE |
| ukb-b-4424 | rs7740402 | G | T | 0.3061 | -0.00951 | 0.00173455 | 6 | 4.09996E-08 | TRUE |
| ukb-b-4424 | rs9345234 | C | A | 0.57786 | 0.009192 | 0.00162351 | 6 | 0.000000015 | TRUE |
| ukb-b-4424 | rs34556183 | G | A | 0.279888 | -0.01335 | 0.00178155 | 6 | 6.59933E-14 | TRUE |
| ukb-b-4424 | rs11982852 | T | C | 0.243858 | -0.01173 | 0.00186248 | 7 | 3.09999E-10 | TRUE |
| ukb-b-4424 | rs62444917 | C | A | 0.222315 | 0.012963 | 0.00192625 | 7 | 1.69981E-11 | TRUE |
| ukb-b-4424 | rs2079070 | G | C | 0.734582 | -0.01344 | 0.00181083 | 7 | 1.20005E-13 | TRUE |
| ukb-b-4424 | rs7831557 | A | G | 0.517438 | -0.01057 | 0.00160135 | 8 | 4.19952E-11 | TRUE |
| ukb-b-4424 | rs7016314 | C | T | 0.655927 | 0.010001 | 0.00168824 | 8 | 3.09999E-09 | TRUE |
| ukb-b-4424 | rs17391944 | G | T | 0.04986 | 0.021852 | 0.00372396 | 9 | 4.39997E-09 | TRUE |
| ukb-b-4424 | rs112100783 | A | G | 0.033439 | -0.02529 | 0.00454852 | 10 | 2.69998E-08 | TRUE |
| ukb-b-4424 | rs10510128 | A | G | 0.207951 | 0.011403 | 0.00197431 | 10 | 7.69999E-09 | TRUE |
| ukb-b-4424 | rs2236295 | T | G | 0.403043 | -0.00908 | 0.00163637 | 10 | 2.90001E-08 | TRUE |
| ukb-b-4424 | rs1517572 | C | A | 0.581256 | 0.011659 | 0.00162182 | 11 | 6.4998E-13 | TRUE |
| ukb-b-4424 | rs7115856 | C | A | 0.461273 | 0.010819 | 0.00160279 | 11 | 1.50003E-11 | TRUE |
| ukb-b-4424 | rs11039216 | T | C | 0.532944 | -0.01028 | 0.00160373 | 11 | 1.5E-10 | TRUE |
| ukb-b-4424 | rs2734831 | G | T | 0.606881 | -0.0098 | 0.00163853 | 11 | 2.19999E-09 | TRUE |
| ukb-b-4424 | rs174564 | G | A | 0.348602 | 0.009745 | 0.001678 | 11 | 6.29999E-09 | TRUE |
| ukb-b-4424 | rs1939455 | T | G | 0.120291 | -0.0158 | 0.0025174 | 11 | 3.50002E-10 | TRUE |
| ukb-b-4424 | rs1553132 | G | A | 0.258638 | 0.010526 | 0.0018253 | 11 | 8.10009E-09 | TRUE |
| ukb-b-4424 | rs34354917 | A | C | 0.288625 | -0.01002 | 0.00176809 | 12 | 1.40001E-08 | TRUE |
| ukb-b-4424 | rs4767550 | G | A | 0.413171 | 0.010873 | 0.00163264 | 12 | 2.70023E-11 | TRUE |
| ukb-b-4424 | rs6561715 | A | T | 0.630636 | 0.009782 | 0.001661 | 13 | 3.89996E-09 | TRUE |
| ukb-b-4424 | rs55658675 | T | C | 0.352906 | -0.00969 | 0.00167463 | 14 | 7.10003E-09 | TRUE |
| ukb-b-4424 | rs11621908 | T | C | 0.082807 | -0.01999 | 0.00294327 | 14 | 1.10002E-11 | TRUE |
| ukb-b-4424 | rs2748809 | C | T | 0.429258 | -0.00925 | 0.00164549 | 14 | 1.89998E-08 | TRUE |
| ukb-b-4424 | rs8038326 | G | A | 0.273169 | -0.01338 | 0.00179311 | 15 | 8.4004E-14 | TRUE |
| ukb-b-4424 | rs56367859 | G | A | 0.397585 | 0.011622 | 0.00163585 | 16 | 1.20005E-12 | TRUE |
| ukb-b-4424 | rs9302680 | A | G | 0.439272 | 0.012044 | 0.00161069 | 16 | 7.59976E-14 | TRUE |
| ukb-b-4424 | rs11643715 | G | C | 0.292625 | 0.010949 | 0.00176035 | 16 | 5E-10 | TRUE |
| ukb-b-4424 | rs8047587 | T | G | 0.439514 | -0.01102 | 0.00161283 | 16 | 8.30042E-12 | TRUE |
| ukb-b-4424 | rs72771082 | G | A | 0.217832 | 0.010974 | 0.00193583 | 16 | 1.40001E-08 | TRUE |
| ukb-b-4424 | rs8074498 | A | T | 0.580951 | -0.00933 | 0.00163392 | 17 | 1.09999E-08 | TRUE |
| ukb-b-4424 | rs11650677 | A | G | 0.339185 | 0.01117 | 0.00168963 | 17 | 3.80014E-11 | TRUE |
| ukb-b-4424 | rs9903898 | T | C | 0.488883 | -0.00945 | 0.00160094 | 17 | 3.59998E-09 | TRUE |
| ukb-b-4424 | rs8072993 | G | T | 0.635677 | 0.010922 | 0.00199248 | 17 | 4.20001E-08 | TRUE |
| ukb-b-4424 | rs1348047 | T | G | 0.267253 | -0.01264 | 0.00182049 | 18 | 3.80014E-12 | TRUE |
| ukb-b-4424 | rs35126035 | C | A | 0.55833 | -0.0092 | 0.00164378 | 19 | 2.19999E-08 | TRUE |
| ukb-b-4424 | rs34786000 | T | G | 0.553361 | 0.010958 | 0.00162787 | 19 | 1.69981E-11 | TRUE |
| ukb-b-4424 | rs2072727 | C | T | 0.56459 | -0.00927 | 0.00161401 | 20 | 9.29994E-09 | TRUE |
| ukb-b-4424 | rs9611007 | T | C | 0.141673 | -0.01359 | 0.00229721 | 22 | 3.29997E-09 | TRUE |

Abbreviations: *β*, the regression coefficient based on the carnitine effect allele; EA, effect allele; EAF, effect allele frequency; IVs, instrumental variants; NEA, non‐effect allele; SE, standard error; SNP, single‐nucleotide polymorphism.

Table S2. Insomnia Genetic IVs

| ID | SNP | EA | NEA | EAF | β | SE | chr | P-value | mr_keep.outcome |
| --- | --- | --- | --- | --- | --- | --- | --- | --- | --- |
| ukb-b-3957 | rs2803296 | C | G | 0.543586 | -0.00862 | 0.00149 | 1 | 7.3E-09 | TRUE |
| ukb-b-3957 | rs12049261 | C | G | 0.292534 | 0.011187 | 0.00163 | 1 | 6.8E-12 | TRUE |
| ukb-b-3957 | rs6690017 | G | T | 0.408855 | -0.01027 | 0.00151 | 1 | 1.1E-11 | TRUE |
| ukb-b-3957 | rs2644128 | G | C | 0.548279 | 0.010628 | 0.001491 | 1 | 1E-12 | TRUE |
| ukb-b-3957 | rs4572538 | T | C | 0.364055 | -0.00961 | 0.001562 | 2 | 7.7E-10 | TRUE |
| ukb-b-3957 | rs56365214 | A | C | 0.155781 | -0.01479 | 0.002052 | 2 | 5.6E-13 | TRUE |
| ukb-b-3957 | rs4577309 | G | A | 0.533655 | -0.00855 | 0.001492 | 2 | 1E-08 | TRUE |
| ukb-b-3957 | rs12470989 | G | A | 0.203934 | -0.01024 | 0.001845 | 2 | 2.8E-08 | TRUE |
| ukb-b-3957 | rs113851554 | T | G | 0.057291 | 0.04678 | 0.003313 | 2 | 2.9E-45 | TRUE |
| ukb-b-3957 | rs56093896 | A | C | 0.214053 | -0.01241 | 0.001814 | 2 | 7.7E-12 | TRUE |
| ukb-b-3957 | rs2014830 | T | C | 0.303519 | -0.0116 | 0.001623 | 3 | 8.9E-13 | TRUE |
| ukb-b-3957 | rs705219 | A | T | 0.887373 | 0.013423 | 0.002353 | 3 | 1.2E-08 | TRUE |
| ukb-b-3957 | rs9845387 | A | C | 0.040279 | -0.02186 | 0.003776 | 3 | 7.1E-09 | TRUE |
| ukb-b-3957 | rs1988337 | G | A | 0.552395 | 0.008387 | 0.001496 | 4 | 2.1E-08 | TRUE |
| ukb-b-3957 | rs11097861 | G | A | 0.716256 | 0.010044 | 0.001649 | 4 | 1.1E-09 | TRUE |
| ukb-b-3957 | rs2604551 | G | T | 0.640384 | -0.00848 | 0.001552 | 4 | 4.7E-08 | TRUE |
| ukb-b-3957 | rs1592757 | C | G | 0.355788 | 0.010222 | 0.00155 | 5 | 4.3E-11 | TRUE |
| ukb-b-3957 | rs7711696 | T | G | 0.305042 | 0.011172 | 0.001611 | 5 | 4.1E-12 | TRUE |
| ukb-b-3957 | rs1430205 | T | C | 0.461507 | 0.009475 | 0.001491 | 5 | 2.1E-10 | TRUE |
| ukb-b-3957 | rs314280 | G | A | 0.547047 | 0.009714 | 0.001491 | 6 | 7.3E-11 | TRUE |
| ukb-b-3957 | rs6975972 | G | A | 0.578726 | -0.00902 | 0.001504 | 7 | 2E-09 | TRUE |
| ukb-b-3957 | rs8180817 | C | G | 0.431016 | -0.01006 | 0.00151 | 7 | 2.7E-11 | TRUE |
| ukb-b-3957 | rs17151854 | T | G | 0.15241 | 0.012989 | 0.002074 | 8 | 3.8E-10 | TRUE |
| ukb-b-3957 | rs11790060 | C | T | 0.330834 | -0.01034 | 0.001579 | 9 | 5.8E-11 | TRUE |
| ukb-b-3957 | rs224032 | A | G | 0.550358 | 0.008391 | 0.001491 | 10 | 1.8E-08 | TRUE |
| ukb-b-3957 | rs17709610 | G | A | 0.297979 | -0.00992 | 0.001621 | 10 | 9.5E-10 | TRUE |
| ukb-b-3957 | rs2297787 | A | T | 0.080193 | -0.0178 | 0.00275 | 10 | 9.6E-11 | TRUE |
| ukb-b-3957 | rs72924721 | T | C | 0.073137 | 0.016478 | 0.002881 | 11 | 1.1E-08 | TRUE |
| ukb-b-3957 | rs10838708 | A | G | 0.45899 | -0.00948 | 0.001503 | 11 | 2.9E-10 | TRUE |
| ukb-b-3957 | rs68094047 | T | C | 0.251267 | 0.010336 | 0.001717 | 12 | 1.7E-09 | TRUE |
| ukb-b-3957 | rs931221 | A | T | 0.236738 | 0.010636 | 0.001753 | 12 | 1.3E-09 | TRUE |
| ukb-b-3957 | rs324017 | C | A | 0.705433 | -0.00988 | 0.001631 | 12 | 1.4E-09 | TRUE |
| ukb-b-3957 | rs9570080 | C | T | 0.344131 | -0.01064 | 0.001579 | 13 | 1.6E-11 | TRUE |
| ukb-b-3957 | rs6561715 | A | T | 0.630662 | -0.01162 | 0.001542 | 13 | 4.8E-14 | TRUE |
| ukb-b-3957 | rs1547630 | A | G | 0.651513 | 0.009108 | 0.001564 | 13 | 5.8E-09 | TRUE |
| ukb-b-3957 | rs4886860 | C | G | 0.767408 | -0.0118 | 0.001756 | 15 | 1.8E-11 | TRUE |
| ukb-b-3957 | rs11635495 | C | T | 0.512179 | 0.009373 | 0.001485 | 15 | 2.8E-10 | TRUE |
| ukb-b-3957 | rs2062113 | C | T | 0.568257 | -0.00962 | 0.001503 | 16 | 1.6E-10 | TRUE |
| ukb-b-3957 | rs9894577 | A | G | 0.3182 | 0.013205 | 0.001597 | 17 | 1.3E-16 | TRUE |
| ukb-b-3957 | rs9906181 | G | A | 0.687567 | -0.00915 | 0.001639 | 17 | 2.4E-08 | TRUE |
| ukb-b-3957 | rs11152363 | A | G | 0.186319 | 0.015639 | 0.001925 | 18 | 4.5E-16 | TRUE |
| ukb-b-3957 | rs56330606 | G | A | 0.378954 | 0.009309 | 0.00153 | 19 | 1.2E-09 | TRUE |

Abbreviations: *β*, the regression coefficient based on the carnitine effect allele; EA, effect allele; EAF, effect allele frequency; IVs, instrumental variants; NEA, non‐effect allele; SE, standard error; SNP, single‐nucleotide polymorphism.

Table S3. Chronotype Genetic IVs

| ID | SNP | EA | NEA | EAF | β | SE | chr | P-value | mr_keep.outcome |
| --- | --- | --- | --- | --- | --- | --- | --- | --- | --- |
| ukb-b-4956 | rs61773390 | T | G | 0.195349 | -0.02567 | 0.002563 | 1 | 1.3E-23 | TRUE |
| ukb-b-4956 | rs72720396 | G | A | 0.229643 | -0.02062 | 0.002414 | 1 | 1.3E-17 | TRUE |
| ukb-b-4956 | rs10737452 | T | C | 0.624486 | 0.014352 | 0.002098 | 1 | 7.9E-12 | TRUE |
| ukb-b-4956 | rs56372114 | T | C | 0.384232 | 0.01213 | 0.002088 | 1 | 6.3E-09 | TRUE |
| ukb-b-4956 | rs7547493 | G | A | 0.177307 | -0.02811 | 0.002657 | 1 | 3.7E-26 | TRUE |
| ukb-b-4956 | rs509476 | C | T | 0.970151 | 0.087817 | 0.00598 | 1 | 8.1E-49 | TRUE |
| ukb-b-4956 | rs17448682 | T | C | 0.232109 | -0.01649 | 0.00241 | 1 | 7.9E-12 | TRUE |
| ukb-b-4956 | rs6658041 | A | G | 0.599474 | -0.012 | 0.002077 | 1 | 7.5E-09 | TRUE |
| ukb-b-4956 | rs12117333 | A | G | 0.077242 | 0.024093 | 0.003802 | 1 | 2.4E-10 | TRUE |
| ukb-b-4956 | rs12140153 | T | G | 0.093978 | 0.026997 | 0.003574 | 1 | 4.2E-14 | TRUE |
| ukb-b-4956 | rs4949980 | G | A | 0.066883 | -0.02293 | 0.004105 | 1 | 2.3E-08 | TRUE |
| ukb-b-4956 | rs17575798 | A | G | 0.193371 | 0.01606 | 0.002568 | 1 | 4E-10 | TRUE |
| ukb-b-4956 | rs11587758 | A | G | 0.396163 | -0.0188 | 0.002073 | 1 | 1.2E-19 | TRUE |
| ukb-b-4956 | rs111761918 | A | G | 0.068055 | 0.022045 | 0.004039 | 1 | 4.8E-08 | TRUE |
| ukb-b-4956 | rs848552 | G | C | 0.527723 | -0.01231 | 0.002034 | 2 | 1.4E-09 | TRUE |
| ukb-b-4956 | rs7586062 | C | G | 0.471841 | 0.018032 | 0.002048 | 2 | 1.3E-18 | TRUE |
| ukb-b-4956 | rs4549082 | C | T | 0.484139 | -0.01531 | 0.002036 | 2 | 5.5E-14 | TRUE |
| ukb-b-4956 | rs13011556 | G | C | 0.239067 | -0.01687 | 0.002393 | 2 | 1.8E-12 | TRUE |
| ukb-b-4956 | rs116131939 | T | C | 0.083611 | -0.02286 | 0.003721 | 2 | 8E-10 | TRUE |
| ukb-b-4956 | rs4671379 | C | T | 0.58559 | -0.01238 | 0.002065 | 2 | 2E-09 | TRUE |
| ukb-b-4956 | rs6718119 | G | A | 0.376746 | -0.01199 | 0.002098 | 2 | 1.1E-08 | TRUE |
| ukb-b-4956 | rs138964083 | T | C | 0.058599 | -0.02569 | 0.004334 | 2 | 3.1E-09 | TRUE |
| ukb-b-4956 | rs57435966 | T | C | 0.08629 | 0.041075 | 0.003623 | 2 | 8.5E-30 | TRUE |
| ukb-b-4956 | rs12713014 | G | A | 0.058725 | 0.026212 | 0.004362 | 2 | 1.9E-09 | TRUE |
| ukb-b-4956 | rs10175975 | T | C | 0.181726 | -0.01838 | 0.002654 | 2 | 4.4E-12 | TRUE |
| ukb-b-4956 | rs28380327 | T | A | 0.369986 | 0.015401 | 0.002104 | 2 | 2.5E-13 | TRUE |
| ukb-b-4956 | rs197273 | G | A | 0.529834 | 0.012096 | 0.002041 | 2 | 3.1E-09 | TRUE |
| ukb-b-4956 | rs114870822 | A | G | 0.012965 | -0.04935 | 0.009026 | 2 | 4.6E-08 | TRUE |
| ukb-b-4956 | rs62182115 | T | C | 0.341565 | 0.013285 | 0.002142 | 2 | 5.5E-10 | TRUE |
| ukb-b-4956 | rs2712056 | T | C | 0.185573 | -0.01573 | 0.002616 | 2 | 1.8E-09 | TRUE |
| ukb-b-4956 | rs2850298 | G | A | 0.698902 | -0.01869 | 0.002214 | 2 | 3.1E-17 | TRUE |
| ukb-b-4956 | rs812925 | G | C | 0.351718 | -0.01512 | 0.002125 | 2 | 1.1E-12 | TRUE |
| ukb-b-4956 | rs62198772 | A | T | 0.410702 | 0.011818 | 0.002077 | 2 | 1.3E-08 | TRUE |
| ukb-b-4956 | rs2706762 | T | C | 0.149575 | 0.018034 | 0.002845 | 2 | 2.3E-10 | TRUE |
| ukb-b-4956 | rs17786957 | C | G | 0.16512 | 0.016311 | 0.002747 | 3 | 2.9E-09 | TRUE |
| ukb-b-4956 | rs114848860 | T | A | 0.024346 | -0.04146 | 0.006623 | 3 | 3.9E-10 | TRUE |
| ukb-b-4956 | rs112555644 | T | C | 0.067556 | -0.02856 | 0.004219 | 3 | 1.3E-11 | TRUE |
| ukb-b-4956 | rs7652260 | G | C | 0.161797 | 0.016002 | 0.002775 | 3 | 8.1E-09 | TRUE |
| ukb-b-4956 | rs13316611 | T | G | 0.255868 | -0.01352 | 0.002333 | 3 | 6.8E-09 | TRUE |
| ukb-b-4956 | rs11714441 | T | C | 0.400637 | 0.011797 | 0.002076 | 3 | 1.3E-08 | TRUE |
| ukb-b-4956 | rs7626349 | C | T | 0.705763 | -0.01254 | 0.002232 | 3 | 1.9E-08 | TRUE |
| ukb-b-4956 | rs34244172 | T | C | 0.292264 | 0.012477 | 0.002249 | 3 | 2.9E-08 | TRUE |
| ukb-b-4956 | rs2239626 | C | T | 0.305676 | -0.01391 | 0.002214 | 3 | 3.3E-10 | TRUE |
| ukb-b-4956 | rs6441169 | A | G | 0.856023 | -0.01618 | 0.002895 | 3 | 2.3E-08 | TRUE |
| ukb-b-4956 | rs9831488 | G | A | 0.353395 | -0.01387 | 0.002148 | 3 | 1.1E-10 | TRUE |
| ukb-b-4956 | rs6442446 | G | A | 0.708837 | 0.01302 | 0.002251 | 3 | 7.2E-09 | TRUE |
| ukb-b-4956 | rs66710942 | T | C | 0.592333 | -0.01331 | 0.00208 | 3 | 1.6E-10 | TRUE |
| ukb-b-4956 | rs4484214 | G | A | 0.314742 | 0.013636 | 0.00219 | 3 | 4.8E-10 | TRUE |
| ukb-b-4956 | rs1800828 | G | C | 0.253098 | 0.013115 | 0.002338 | 3 | 2E-08 | TRUE |
| ukb-b-4956 | rs149611468 | C | T | 0.011957 | 0.058505 | 0.009546 | 3 | 8.9E-10 | TRUE |
| ukb-b-4956 | rs13059636 | G | A | 0.469784 | -0.0134 | 0.002047 | 3 | 6E-11 | TRUE |
| ukb-b-4956 | rs1135946 | C | T | 0.232423 | 0.01727 | 0.002405 | 4 | 6.9E-13 | TRUE |
| ukb-b-4956 | rs4241964 | G | T | 0.475791 | -0.01523 | 0.002047 | 4 | 1E-13 | TRUE |
| ukb-b-4956 | rs10058356 | T | C | 0.697815 | 0.013099 | 0.002221 | 5 | 3.7E-09 | TRUE |
| ukb-b-4956 | rs147762489 | T | C | 0.249106 | 0.016287 | 0.00236 | 5 | 5.2E-12 | TRUE |
| ukb-b-4956 | rs10461917 | C | T | 0.689585 | 0.012321 | 0.00221 | 5 | 2.5E-08 | TRUE |
| ukb-b-4956 | rs4518438 | C | T | 0.510399 | -0.01526 | 0.002035 | 5 | 6.3E-14 | TRUE |
| ukb-b-4956 | rs286808 | C | T | 0.524555 | 0.011713 | 0.00204 | 5 | 9.4E-09 | TRUE |
| ukb-b-4956 | rs9291813 | C | T | 0.760663 | -0.01339 | 0.002383 | 5 | 1.9E-08 | TRUE |
| ukb-b-4956 | rs1976423 | C | A | 0.495282 | -0.014 | 0.002037 | 5 | 6.4E-12 | TRUE |
| ukb-b-4956 | rs67988891 | G | C | 0.318639 | -0.01872 | 0.002187 | 5 | 1.1E-17 | TRUE |
| ukb-b-4956 | rs7735794 | A | G | 0.223944 | -0.01517 | 0.002579 | 5 | 4E-09 | TRUE |
| ukb-b-4956 | rs610590 | G | C | 0.211955 | -0.01464 | 0.002498 | 6 | 4.6E-09 | TRUE |
| ukb-b-4956 | rs76223855 | C | T | 0.010981 | -0.07986 | 0.009755 | 6 | 2.7E-16 | TRUE |
| ukb-b-4956 | rs12525312 | C | T | 0.550602 | 0.012291 | 0.002045 | 6 | 1.9E-09 | TRUE |
| ukb-b-4956 | rs9395520 | T | C | 0.304164 | -0.01737 | 0.002212 | 6 | 4.1E-15 | TRUE |
| ukb-b-4956 | rs35101255 | G | A | 0.079468 | 0.024415 | 0.003761 | 6 | 8.5E-11 | TRUE |
| ukb-b-4956 | rs9348050 | C | T | 0.510601 | 0.012457 | 0.002036 | 6 | 9.5E-10 | TRUE |
| ukb-b-4956 | rs486416 | A | G | 0.636771 | 0.011578 | 0.002103 | 6 | 3.7E-08 | TRUE |
| ukb-b-4956 | rs1983891 | T | C | 0.276348 | 0.012909 | 0.002241 | 6 | 8.5E-09 | TRUE |
| ukb-b-4956 | rs2653349 | G | A | 0.787049 | 0.028396 | 0.002484 | 6 | 2.9E-30 | TRUE |
| ukb-b-4956 | rs9476310 | T | C | 0.51056 | -0.0126 | 0.002039 | 6 | 6.5E-10 | TRUE |
| ukb-b-4956 | rs2881955 | T | C | 0.278459 | -0.01363 | 0.002273 | 6 | 2E-09 | TRUE |
| ukb-b-4956 | rs1996399 | A | G | 0.300648 | -0.01217 | 0.002222 | 7 | 4.3E-08 | TRUE |
| ukb-b-4956 | rs202157 | T | C | 0.701066 | 0.018979 | 0.002229 | 7 | 1.7E-17 | TRUE |
| ukb-b-4956 | rs2971970 | G | T | 0.782046 | 0.015157 | 0.002467 | 7 | 8E-10 | TRUE |
| ukb-b-4956 | rs17161045 | C | T | 0.369932 | 0.015343 | 0.002121 | 7 | 4.7E-13 | TRUE |
| ukb-b-4956 | rs4729854 | A | T | 0.482 | 0.02167 | 0.002082 | 7 | 2.3E-25 | TRUE |
| ukb-b-4956 | rs7783012 | A | G | 0.590654 | -0.01212 | 0.002075 | 7 | 5.2E-09 | TRUE |
| ukb-b-4956 | rs10280205 | C | T | 0.309279 | 0.013315 | 0.002203 | 7 | 1.5E-09 | TRUE |
| ukb-b-4956 | rs6967481 | T | C | 0.497073 | -0.01552 | 0.002043 | 7 | 3E-14 | TRUE |
| ukb-b-4956 | rs10954933 | G | A | 0.42758 | -0.01548 | 0.002062 | 8 | 6E-14 | TRUE |
| ukb-b-4956 | rs769066 | C | T | 0.18416 | -0.01654 | 0.002631 | 8 | 3.2E-10 | TRUE |
| ukb-b-4956 | rs3100052 | G | A | 0.613151 | 0.012203 | 0.00209 | 8 | 5.2E-09 | TRUE |
| ukb-b-4956 | rs13258797 | A | G | 0.168546 | -0.01588 | 0.002717 | 8 | 5E-09 | TRUE |
| ukb-b-4956 | rs17716502 | T | C | 0.204456 | -0.01884 | 0.002542 | 8 | 1.2E-13 | TRUE |
| ukb-b-4956 | rs4321976 | C | T | 0.221209 | 0.017206 | 0.002452 | 8 | 2.3E-12 | TRUE |
| ukb-b-4956 | rs35524253 | A | G | 0.356248 | -0.01211 | 0.00213 | 8 | 1.3E-08 | TRUE |
| ukb-b-4956 | rs6601686 | T | A | 0.410116 | 0.013711 | 0.002083 | 8 | 4.6E-11 | TRUE |
| ukb-b-4956 | rs12377175 | C | A | 0.22858 | 0.015536 | 0.002362 | 9 | 4.8E-11 | TRUE |
| ukb-b-4956 | rs10988239 | T | C | 0.512284 | 0.012885 | 0.002069 | 9 | 4.7E-10 | TRUE |
| ukb-b-4956 | rs62553781 | T | C | 0.034554 | 0.037935 | 0.005581 | 9 | 1.1E-11 | TRUE |
| ukb-b-4956 | rs113171806 | C | T | 0.11444 | 0.018143 | 0.0032 | 9 | 1.4E-08 | TRUE |
| ukb-b-4956 | rs28458909 | T | C | 0.122383 | 0.02988 | 0.003098 | 9 | 5.2E-22 | TRUE |
| ukb-b-4956 | rs2291589 | G | T | 0.377049 | 0.015191 | 0.002098 | 9 | 4.5E-13 | TRUE |
| ukb-b-4956 | rs10118767 | T | C | 0.197744 | 0.013967 | 0.002553 | 9 | 4.5E-08 | TRUE |
| ukb-b-4956 | rs57994353 | C | T | 0.299321 | -0.0128 | 0.002218 | 9 | 7.8E-09 | TRUE |
| ukb-b-4956 | rs2893787 | A | G | 0.743579 | 0.014813 | 0.002327 | 10 | 1.9E-10 | TRUE |
| ukb-b-4956 | rs12249410 | T | G | 0.109634 | 0.018994 | 0.003319 | 10 | 1E-08 | TRUE |
| ukb-b-4956 | rs3808964 | T | G | 0.633453 | -0.01212 | 0.002114 | 10 | 1E-08 | TRUE |
| ukb-b-4956 | rs1914772 | A | T | 0.894653 | 0.021161 | 0.003368 | 11 | 3.3E-10 | TRUE |
| ukb-b-4956 | rs4936291 | G | A | 0.389142 | -0.01363 | 0.002144 | 11 | 2.1E-10 | TRUE |
| ukb-b-4956 | rs74357745 | G | A | 0.12053 | 0.020238 | 0.003126 | 11 | 9.6E-11 | TRUE |
| ukb-b-4956 | rs9795439 | G | A | 0.803776 | 0.014776 | 0.002561 | 11 | 7.9E-09 | TRUE |
| ukb-b-4956 | rs4141920 | A | G | 0.455163 | 0.011652 | 0.002045 | 11 | 1.2E-08 | TRUE |
| ukb-b-4956 | rs2077432 | T | C | 0.269742 | -0.0134 | 0.0023 | 11 | 5.7E-09 | TRUE |
| ukb-b-4956 | rs3729986 | T | C | 0.10224 | -0.0189 | 0.00335 | 11 | 1.7E-08 | TRUE |
| ukb-b-4956 | rs4237555 | T | C | 0.527732 | -0.0117 | 0.002038 | 11 | 9.4E-09 | TRUE |
| ukb-b-4956 | rs72632979 | G | A | 0.172073 | 0.016548 | 0.002703 | 11 | 9.2E-10 | TRUE |
| ukb-b-4956 | rs10742179 | G | A | 0.739373 | 0.012901 | 0.002318 | 11 | 2.6E-08 | TRUE |
| ukb-b-4956 | rs11032362 | A | G | 0.090981 | -0.02622 | 0.003534 | 11 | 1.2E-13 | TRUE |
| ukb-b-4956 | rs3168135 | A | G | 0.24042 | 0.01497 | 0.00238 | 11 | 3.2E-10 | TRUE |
| ukb-b-4956 | rs12811046 | G | A | 0.445344 | 0.012957 | 0.002046 | 12 | 2.4E-10 | TRUE |
| ukb-b-4956 | rs11181153 | T | C | 0.586892 | 0.018015 | 0.002067 | 12 | 2.9E-18 | TRUE |
| ukb-b-4956 | rs4595586 | T | A | 0.506544 | 0.022753 | 0.002039 | 12 | 6.4E-29 | TRUE |
| ukb-b-4956 | rs7308565 | T | C | 0.399536 | -0.01475 | 0.002096 | 12 | 2E-12 | TRUE |
| ukb-b-4956 | rs7304278 | G | A | 0.723741 | -0.01472 | 0.002283 | 12 | 1.1E-10 | TRUE |
| ukb-b-4956 | rs80097534 | T | G | 0.098204 | 0.022313 | 0.003452 | 12 | 1E-10 | TRUE |
| ukb-b-4956 | rs7316768 | T | G | 0.400891 | 0.014223 | 0.002079 | 12 | 7.8E-12 | TRUE |
| ukb-b-4956 | rs11183201 | C | T | 0.508456 | -0.01292 | 0.00204 | 12 | 2.4E-10 | TRUE |
| ukb-b-4956 | rs74097630 | T | G | 0.140832 | 0.018245 | 0.002932 | 12 | 4.8E-10 | TRUE |
| ukb-b-4956 | rs7959983 | C | T | 0.405084 | -0.01408 | 0.002064 | 12 | 9E-12 | TRUE |
| ukb-b-4956 | rs9573971 | G | A | 0.03387 | 0.052568 | 0.005629 | 13 | 9.7E-21 | TRUE |
| ukb-b-4956 | rs2762088 | G | T | 0.761336 | -0.01447 | 0.002396 | 13 | 1.6E-09 | TRUE |
| ukb-b-4956 | rs17517 | A | G | 0.511747 | 0.011642 | 0.002049 | 13 | 1.3E-08 | TRUE |
| ukb-b-4956 | rs9597250 | A | C | 0.189491 | 0.016084 | 0.002603 | 13 | 6.5E-10 | TRUE |
| ukb-b-4956 | rs1927719 | A | T | 0.765682 | -0.01398 | 0.002412 | 13 | 6.7E-09 | TRUE |
| ukb-b-4956 | rs12432176 | A | C | 0.379609 | -0.01208 | 0.002106 | 14 | 9.7E-09 | TRUE |
| ukb-b-4956 | rs10149448 | G | A | 0.396431 | 0.011546 | 0.002083 | 14 | 3E-08 | TRUE |
| ukb-b-4956 | rs698015 | T | C | 0.646935 | -0.01313 | 0.002161 | 14 | 1.2E-09 | TRUE |
| ukb-b-4956 | rs7148842 | T | C | 0.386473 | 0.012183 | 0.002107 | 14 | 7.4E-09 | TRUE |
| ukb-b-4956 | rs1439319 | C | G | 0.645445 | 0.012165 | 0.002144 | 15 | 1.4E-08 | TRUE |
| ukb-b-4956 | rs2701524 | C | T | 0.413565 | 0.011493 | 0.002079 | 15 | 3.2E-08 | TRUE |
| ukb-b-4956 | rs4886947 | A | G | 0.660074 | 0.011932 | 0.002163 | 15 | 3.5E-08 | TRUE |
| ukb-b-4956 | rs4784655 | C | G | 0.321967 | 0.01634 | 0.002184 | 16 | 7.4E-14 | TRUE |
| ukb-b-4956 | rs12927162 | G | A | 0.276664 | 0.020948 | 0.002274 | 16 | 3.2E-20 | TRUE |
| ukb-b-4956 | rs1421085 | C | T | 0.403565 | -0.02085 | 0.002074 | 16 | 8.9E-24 | TRUE |
| ukb-b-4956 | rs9932577 | A | C | 0.505674 | 0.012651 | 0.002055 | 16 | 7.4E-10 | TRUE |
| ukb-b-4956 | rs17604349 | A | G | 0.179748 | 0.021948 | 0.002656 | 16 | 1.4E-16 | TRUE |
| ukb-b-4956 | rs3760185 | T | C | 0.247661 | 0.018906 | 0.002384 | 17 | 2.2E-15 | TRUE |
| ukb-b-4956 | rs225298 | G | T | 0.830934 | 0.015177 | 0.002712 | 17 | 2.2E-08 | TRUE |
| ukb-b-4956 | rs6504758 | G | A | 0.535685 | -0.01247 | 0.00205 | 17 | 1.2E-09 | TRUE |
| ukb-b-4956 | rs2364972 | G | A | 0.462953 | -0.0128 | 0.002044 | 17 | 3.7E-10 | TRUE |
| ukb-b-4956 | rs72829706 | G | A | 0.039463 | 0.029534 | 0.005242 | 17 | 1.8E-08 | TRUE |
| ukb-b-4956 | rs2518022 | C | T | 0.914438 | 0.030702 | 0.003628 | 17 | 2.6E-17 | TRUE |
| ukb-b-4956 | rs12965577 | G | A | 0.334996 | 0.015874 | 0.002159 | 18 | 2E-13 | TRUE |
| ukb-b-4956 | rs12969848 | T | C | 0.529314 | -0.01642 | 0.002045 | 18 | 9.6E-16 | TRUE |
| ukb-b-4956 | rs62082401 | G | C | 0.191147 | -0.02005 | 0.002588 | 18 | 9.6E-15 | TRUE |
| ukb-b-4956 | rs9962650 | G | C | 0.422583 | -0.01392 | 0.002063 | 18 | 1.5E-11 | TRUE |
| ukb-b-4956 | rs9964420 | A | C | 0.302896 | 0.021488 | 0.002224 | 18 | 4.4E-22 | TRUE |
| ukb-b-4956 | rs56076457 | T | C | 0.529246 | -0.01172 | 0.002038 | 18 | 8.9E-09 | TRUE |
| ukb-b-4956 | rs10460095 | A | G | 0.574433 | -0.0134 | 0.002063 | 18 | 8.1E-11 | TRUE |
| ukb-b-4956 | rs10402849 | T | C | 0.201704 | -0.01517 | 0.00254 | 19 | 2.3E-09 | TRUE |
| ukb-b-4956 | rs12971913 | A | G | 0.448148 | 0.012664 | 0.002051 | 19 | 6.6E-10 | TRUE |
| ukb-b-4956 | rs12462111 | T | C | 0.464858 | 0.013166 | 0.002067 | 19 | 1.9E-10 | TRUE |
| ukb-b-4956 | rs1874493 | G | A | 0.680061 | 0.0123 | 0.002183 | 19 | 1.8E-08 | TRUE |
| ukb-b-4956 | rs78095690 | C | T | 0.437331 | -0.01149 | 0.002058 | 20 | 2.4E-08 | TRUE |
| ukb-b-4956 | rs2072727 | C | T | 0.564423 | 0.011655 | 0.002053 | 20 | 1.4E-08 | TRUE |
| ukb-b-4956 | rs6131942 | G | A | 0.579505 | -0.01431 | 0.002067 | 20 | 4.5E-12 | TRUE |
| ukb-b-4956 | rs1056322 | G | C | 0.321001 | 0.012641 | 0.002179 | 22 | 6.6E-09 | TRUE |
| ukb-b-4956 | rs139911 | T | C | 0.575785 | 0.01755 | 0.002067 | 22 | 2E-17 | TRUE |

Abbreviations: *β*, the regression coefficient based on the carnitine effect allele; EA, effect allele; EAF, effect allele frequency; IVs, instrumental variants; NEA, non‐effect allele; SE, standard error; SNP, single‐nucleotide polymorphism.

Table S4. Daytime Dozing IVs

| ID | SNP | EA | NEA | EAF | β | SE | chr | P-value | mr_keep.outcome |
| --- | --- | --- | --- | --- | --- | --- | --- | --- | --- |
| ukb-b-5776 | rs2787120 | G | A | 0.168023 | -0.00765 | 0.001377 | 1 | 2.8E-08 | TRUE |
| ukb-b-5776 | rs12140153 | T | G | 0.094277 | -0.01693 | 0.001807 | 1 | 7.4E-21 | TRUE |
| ukb-b-5776 | rs57746981 | T | C | 0.35614 | -0.0069 | 0.001076 | 1 | 1.5E-10 | TRUE |
| ukb-b-5776 | rs553314 | C | T | 0.635125 | -0.00668 | 0.001078 | 1 | 5.7E-10 | TRUE |
| ukb-b-5776 | rs825127 | G | T | 0.468559 | -0.00583 | 0.001034 | 1 | 1.7E-08 | TRUE |
| ukb-b-5776 | rs4286272 | C | T | 0.532938 | -0.00595 | 0.001033 | 2 | 8.5E-09 | TRUE |
| ukb-b-5776 | rs4665972 | C | T | 0.604477 | -0.00671 | 0.001058 | 2 | 2.3E-10 | TRUE |
| ukb-b-5776 | rs55767040 | C | T | 0.302656 | -0.00713 | 0.001128 | 2 | 2.5E-10 | TRUE |
| ukb-b-5776 | rs11123962 | G | T | 0.44596 | 0.00792 | 0.001036 | 2 | 2.1E-14 | TRUE |
| ukb-b-5776 | rs7607363 | G | A | 0.437902 | 0.00601 | 0.00104 | 2 | 7.4E-09 | TRUE |
| ukb-b-5776 | rs13010456 | G | A | 0.405192 | -0.00765 | 0.001052 | 2 | 3.5E-13 | TRUE |
| ukb-b-5776 | rs34478464 | T | C | 0.191618 | 0.008843 | 0.001307 | 3 | 1.3E-11 | TRUE |
| ukb-b-5776 | rs843372 | T | C | 0.770761 | -0.00824 | 0.001229 | 3 | 2E-11 | TRUE |
| ukb-b-5776 | rs59262698 | C | T | 0.413259 | -0.00668 | 0.001046 | 5 | 1.7E-10 | TRUE |
| ukb-b-5776 | rs11242450 | T | C | 0.523658 | -0.00684 | 0.001034 | 5 | 3.7E-11 | TRUE |
| ukb-b-5776 | rs56143617 | T | C | 0.179101 | -0.0079 | 0.001348 | 6 | 4.6E-09 | TRUE |
| ukb-b-5776 | rs3122170 | A | C | 0.770296 | -0.00924 | 0.00123 | 6 | 5.7E-14 | TRUE |
| ukb-b-5776 | rs6923811 | C | T | 0.322762 | -0.00659 | 0.001104 | 6 | 2.4E-09 | TRUE |
| ukb-b-5776 | rs62519822 | A | G | 0.11231 | 0.009559 | 0.001632 | 8 | 4.7E-09 | TRUE |
| ukb-b-5776 | rs285793 | A | G | 0.539876 | -0.00659 | 0.001036 | 8 | 2E-10 | TRUE |
| ukb-b-5776 | rs1566362 | C | T | 0.367775 | -0.00668 | 0.00107 | 9 | 4.4E-10 | TRUE |
| ukb-b-5776 | rs13284688 | C | T | 0.207093 | 0.009436 | 0.001272 | 9 | 1.2E-13 | TRUE |
| ukb-b-5776 | rs7476897 | A | G | 0.319441 | -0.00699 | 0.001104 | 10 | 2.5E-10 | TRUE |
| ukb-b-5776 | rs4765939 | C | G | 0.418059 | 0.006157 | 0.001046 | 12 | 4E-09 | TRUE |
| ukb-b-5776 | rs2160515 | G | A | 0.437142 | 0.005796 | 0.001047 | 12 | 3.1E-08 | TRUE |
| ukb-b-5776 | rs1846644 | C | T | 0.408096 | 0.011303 | 0.001049 | 12 | 4.6E-27 | TRUE |
| ukb-b-5776 | rs8015449 | G | A | 0.461183 | -0.00619 | 0.001036 | 14 | 2.2E-09 | TRUE |
| ukb-b-5776 | rs17356118 | G | A | 0.231686 | 0.007241 | 0.001221 | 15 | 3E-09 | TRUE |
| ukb-b-5776 | rs886114 | T | C | 0.641076 | -0.00607 | 0.001074 | 16 | 1.6E-08 | TRUE |
| ukb-b-5776 | rs11078398 | A | G | 0.257546 | -0.00744 | 0.00124 | 17 | 1.9E-09 | TRUE |
| ukb-b-5776 | rs7207582 | G | A | 0.228698 | -0.00847 | 0.001227 | 17 | 5.1E-12 | TRUE |

Abbreviations: *β*, the regression coefficient based on the carnitine effect allele; EA, effect allele; EAF, effect allele frequency; IVs, instrumental variants; NEA, non‐effect allele; SE, standard error; SNP, single‐nucleotide polymorphism.

Table S5. Napping during the Day IVs

| ID | SNP | EA | NEA | EAF | β | SE | chr | P-value | mr_keep.outcome |
| --- | --- | --- | --- | --- | --- | --- | --- | --- | --- |
| ukb-b-4616 | rs6696864 | A | T | 0.401498 | -0.007042 | 0.001255 | 1 | 2E-08 | TRUE |
| ukb-b-4616 | rs2786547 | T | C | 0.177277 | -0.010988 | 0.001602 | 1 | 6.8E-12 | TRUE |
| ukb-b-4616 | rs12042846 | C | T | 0.178571 | 0.009054 | 0.001607 | 1 | 1.8E-08 | TRUE |
| ukb-b-4616 | rs7555990 | T | C | 0.13587 | -0.010372 | 0.00179 | 1 | 6.8E-09 | TRUE |
| ukb-b-4616 | rs11121194 | T | C | 0.633703 | 0.0070632 | 0.001266 | 1 | 2.4E-08 | TRUE |
| ukb-b-4616 | rs6697561 | T | C | 0.223435 | 0.0096806 | 0.001516 | 1 | 1.7E-10 | TRUE |
| ukb-b-4616 | rs903678 | A | G | 0.336999 | 0.013653 | 0.001293 | 1 | 4.6E-26 | TRUE |
| ukb-b-4616 | rs12140153 | T | G | 0.09424 | -0.024271 | 0.002147 | 1 | 1.2E-29 | TRUE |
| ukb-b-4616 | rs1931175 | G | C | 0.38274 | 0.0074612 | 0.00126 | 1 | 3.2E-09 | TRUE |
| ukb-b-4616 | rs75022160 | T | C | 0.1366 | -0.009763 | 0.00179 | 2 | 4.9E-08 | TRUE |
| ukb-b-4616 | rs11688767 | T | A | 0.486928 | 0.0073185 | 0.001226 | 2 | 2.4E-09 | TRUE |
| ukb-b-4616 | rs2390669 | C | A | 0.128947 | -0.011278 | 0.001837 | 2 | 8.4E-10 | TRUE |
| ukb-b-4616 | rs13023587 | G | C | 0.508078 | -0.007927 | 0.001224 | 2 | 9.3E-11 | TRUE |
| ukb-b-4616 | rs12615434 | T | C | 0.11309 | 0.0116344 | 0.001933 | 2 | 1.8E-09 | TRUE |
| ukb-b-4616 | rs13033444 | G | A | 0.281934 | 0.0094407 | 0.001365 | 2 | 4.6E-12 | TRUE |
| ukb-b-4616 | rs7422655 | T | C | 0.736305 | -0.008053 | 0.001391 | 2 | 7.1E-09 | TRUE |
| ukb-b-4616 | rs908442 | T | A | 0.408474 | -0.009771 | 0.001247 | 2 | 4.7E-15 | TRUE |
| ukb-b-4616 | rs11125776 | G | T | 0.144182 | -0.012041 | 0.001748 | 2 | 5.6E-12 | TRUE |
| ukb-b-4616 | rs12992648 | G | A | 0.281533 | -0.007626 | 0.001361 | 2 | 2.1E-08 | TRUE |
| ukb-b-4616 | rs75411336 | T | C | 0.054402 | -0.015756 | 0.0027 | 3 | 5.4E-09 | TRUE |
| ukb-b-4616 | rs40005 | A | G | 0.76889 | 0.0080725 | 0.001454 | 3 | 2.8E-08 | TRUE |
| ukb-b-4616 | rs1001817 | T | C | 0.494347 | -0.007767 | 0.001225 | 3 | 2.3E-10 | TRUE |
| ukb-b-4616 | rs77154532 | G | A | 0.359504 | -0.007242 | 0.001281 | 3 | 1.6E-08 | TRUE |
| ukb-b-4616 | rs4856536 | A | G | 0.728768 | -0.008597 | 0.001381 | 3 | 4.8E-10 | TRUE |
| ukb-b-4616 | rs9998136 | G | C | 0.748397 | 0.0094814 | 0.001417 | 4 | 2.2E-11 | TRUE |
| ukb-b-4616 | rs113886333 | T | C | 0.036115 | 0.0182674 | 0.003298 | 4 | 3E-08 | TRUE |
| ukb-b-4616 | rs7698842 | G | A | 0.741815 | 0.0077781 | 0.001417 | 4 | 4E-08 | TRUE |
| ukb-b-4616 | rs4692709 | T | C | 0.546706 | -0.007001 | 0.001236 | 4 | 1.5E-08 | TRUE |
| ukb-b-4616 | rs12657723 | T | C | 0.320683 | 0.0081807 | 0.001312 | 5 | 4.4E-10 | TRUE |
| ukb-b-4616 | rs467897 | A | G | 0.68009 | -0.009309 | 0.001315 | 5 | 1.5E-12 | TRUE |
| ukb-b-4616 | rs2431108 | C | T | 0.328424 | 0.012511 | 0.001302 | 5 | 7.5E-22 | TRUE |
| ukb-b-4616 | rs10875622 | A | G | 0.575874 | 0.0102674 | 0.001241 | 5 | 1.3E-16 | TRUE |
| ukb-b-4616 | rs6452787 | G | A | 0.466869 | -0.007245 | 0.001227 | 5 | 3.5E-09 | TRUE |
| ukb-b-4616 | rs2099810 | G | A | 0.496352 | -0.007808 | 0.001225 | 5 | 1.9E-10 | TRUE |
| ukb-b-4616 | rs72781017 | G | A | 0.402575 | -0.007568 | 0.001248 | 5 | 1.3E-09 | TRUE |
| ukb-b-4616 | rs2653349 | G | A | 0.787137 | -0.016308 | 0.001495 | 6 | 1E-27 | TRUE |
| ukb-b-4616 | rs7752899 | T | C | 0.442806 | 0.0084798 | 0.001231 | 6 | 5.7E-12 | TRUE |
| ukb-b-4616 | rs62425620 | T | C | 0.369978 | 0.0077519 | 0.001276 | 6 | 1.2E-09 | TRUE |
| ukb-b-4616 | rs34262487 | A | C | 0.072621 | -0.014638 | 0.002366 | 6 | 6.1E-10 | TRUE |
| ukb-b-4616 | rs6919087 | G | T | 0.311989 | -0.010382 | 0.001323 | 6 | 4.3E-15 | TRUE |
| ukb-b-4616 | rs2143792 | A | G | 0.431158 | -0.007216 | 0.001245 | 6 | 6.8E-09 | TRUE |
| ukb-b-4616 | rs1856502 | A | T | 0.456906 | 0.007104 | 0.001228 | 6 | 7.2E-09 | TRUE |
| ukb-b-4616 | rs785145 | G | T | 0.431525 | 0.0070649 | 0.001236 | 6 | 1.1E-08 | TRUE |
| ukb-b-4616 | rs614987 | C | A | 0.613566 | 0.0107556 | 0.001259 | 6 | 1.3E-17 | TRUE |
| ukb-b-4616 | rs9467772 | T | A | 0.199731 | -0.009118 | 0.001528 | 6 | 2.4E-09 | TRUE |
| ukb-b-4616 | rs35851551 | G | A | 0.101129 | -0.01195 | 0.002049 | 7 | 5.5E-09 | TRUE |
| ukb-b-4616 | rs1011024 | G | A | 0.154712 | -0.010227 | 0.001698 | 7 | 1.7E-09 | TRUE |
| ukb-b-4616 | rs6942927 | A | G | 0.12313 | 0.0146516 | 0.002171 | 7 | 1.5E-11 | TRUE |
| ukb-b-4616 | rs7814873 | T | C | 0.616133 | -0.007045 | 0.001275 | 8 | 3.3E-08 | TRUE |
| ukb-b-4616 | rs351776 | C | A | 0.548043 | 0.007626 | 0.00123 | 8 | 5.7E-10 | TRUE |
| ukb-b-4616 | rs13266972 | G | A | 0.698405 | -0.007295 | 0.001335 | 8 | 4.7E-08 | TRUE |
| ukb-b-4616 | rs285815 | A | T | 0.546163 | -0.007384 | 0.001231 | 8 | 2E-09 | TRUE |
| ukb-b-4616 | rs10757347 | G | A | 0.222815 | 0.0081036 | 0.001476 | 9 | 4.1E-08 | TRUE |
| ukb-b-4616 | rs971415 | G | A | 0.122716 | -0.010834 | 0.001865 | 9 | 6.3E-09 | TRUE |
| ukb-b-4616 | rs12346996 | C | T | 0.728608 | -0.008322 | 0.001378 | 9 | 1.5E-09 | TRUE |
| ukb-b-4616 | rs10868046 | A | G | 0.603704 | 0.0072737 | 0.001261 | 9 | 7.9E-09 | TRUE |
| ukb-b-4616 | rs7038206 | G | A | 0.608433 | 0.0073823 | 0.001255 | 9 | 4E-09 | TRUE |
| ukb-b-4616 | rs62560863 | T | C | 0.100702 | 0.0114884 | 0.002039 | 9 | 1.8E-08 | TRUE |
| ukb-b-4616 | rs13284688 | C | T | 0.207055 | 0.0144228 | 0.001511 | 9 | 1.4E-21 | TRUE |
| ukb-b-4616 | rs11252681 | A | G | 0.045481 | 0.016111 | 0.002937 | 10 | 4.1E-08 | TRUE |
| ukb-b-4616 | rs224111 | A | G | 0.388797 | -0.007842 | 0.001259 | 10 | 4.6E-10 | TRUE |
| ukb-b-4616 | rs11258652 | A | C | 0.235891 | -0.010013 | 0.001444 | 10 | 4.1E-12 | TRUE |
| ukb-b-4616 | rs10764260 | A | G | 0.363097 | -0.007333 | 0.001281 | 10 | 1E-08 | TRUE |
| ukb-b-4616 | rs4587762 | A | G | 0.604757 | -0.006941 | 0.001256 | 11 | 3.3E-08 | TRUE |
| ukb-b-4616 | rs10835420 | A | T | 0.248826 | -0.00858 | 0.001415 | 11 | 1.3E-09 | TRUE |
| ukb-b-4616 | rs174541 | C | T | 0.35911 | 0.0101856 | 0.001275 | 11 | 1.4E-15 | TRUE |
| ukb-b-4616 | rs6483215 | G | A | 0.764113 | -0.008418 | 0.001442 | 11 | 5.3E-09 | TRUE |
| ukb-b-4616 | rs11224896 | C | T | 0.110091 | -0.011186 | 0.001956 | 11 | 1.1E-08 | TRUE |
| ukb-b-4616 | rs10840017 | G | A | 0.233223 | -0.008666 | 0.001493 | 11 | 6.4E-09 | TRUE |
| ukb-b-4616 | rs4402351 | G | A | 0.148069 | -0.011765 | 0.001733 | 12 | 1.1E-11 | TRUE |
| ukb-b-4616 | rs1479116 | A | G | 0.351118 | 0.0079645 | 0.001295 | 12 | 7.8E-10 | TRUE |
| ukb-b-4616 | rs35011311 | T | G | 0.264996 | -0.009469 | 0.001396 | 12 | 1.2E-11 | TRUE |
| ukb-b-4616 | rs11615756 | T | C | 0.403516 | 0.0182385 | 0.001251 | 12 | 3.7E-48 | TRUE |
| ukb-b-4616 | rs2769916 | A | G | 0.688798 | 0.0087737 | 0.001326 | 13 | 3.6E-11 | TRUE |
| ukb-b-4616 | rs2370926 | C | T | 0.367305 | -0.008283 | 0.001274 | 14 | 7.9E-11 | TRUE |
| ukb-b-4616 | rs10150432 | G | A | 0.184721 | 0.0104521 | 0.00158 | 14 | 3.7E-11 | TRUE |
| ukb-b-4616 | rs11071755 | A | G | 0.424644 | -0.00696 | 0.001239 | 15 | 1.9E-08 | TRUE |
| ukb-b-4616 | rs17158413 | A | G | 0.237392 | 0.0085573 | 0.001442 | 15 | 3E-09 | TRUE |
| ukb-b-4616 | rs7191614 | G | A | 0.290789 | 0.0076981 | 0.001353 | 16 | 1.3E-08 | TRUE |
| ukb-b-4616 | rs7198121 | C | T | 0.538397 | -0.00682 | 0.001231 | 16 | 3E-08 | TRUE |
| ukb-b-4616 | rs60920123 | A | G | 0.432894 | -0.007449 | 0.001238 | 16 | 1.8E-09 | TRUE |
| ukb-b-4616 | rs8050478 | A | G | 0.500601 | -0.007635 | 0.001225 | 16 | 4.6E-10 | TRUE |
| ukb-b-4616 | rs2668643 | A | C | 0.224123 | -0.021199 | 0.001469 | 17 | 3.4E-47 | TRUE |
| ukb-b-4616 | rs3935190 | A | G | 0.536792 | 0.0082697 | 0.001235 | 17 | 2.1E-11 | TRUE |
| ukb-b-4616 | rs12451365 | C | T | 0.204133 | 0.0108452 | 0.001519 | 17 | 9.3E-13 | TRUE |
| ukb-b-4616 | rs962247 | A | G | 0.475853 | -0.00818 | 0.001236 | 18 | 3.6E-11 | TRUE |
| ukb-b-4616 | rs9965170 | A | G | 0.423866 | -0.014193 | 0.001239 | 18 | 2.3E-30 | TRUE |
| ukb-b-4616 | rs17816756 | C | T | 0.16373 | 0.0091996 | 0.00166 | 18 | 3E-08 | TRUE |
| ukb-b-4616 | rs2033103 | T | C | 0.450439 | 0.0075353 | 0.001231 | 18 | 9.3E-10 | TRUE |
| ukb-b-4616 | rs3810484 | G | A | 0.443064 | -0.007106 | 0.001233 | 20 | 8.2E-09 | TRUE |
| ukb-b-4616 | rs17265513 | C | T | 0.198242 | 0.0088806 | 0.001536 | 20 | 7.3E-09 | TRUE |
| ukb-b-4616 | rs910187 | A | G | 0.373013 | -0.00724 | 0.001267 | 20 | 1.1E-08 | TRUE |
| ukb-b-4616 | rs1883048 | C | T | 0.524834 | 0.0078482 | 0.001233 | 21 | 2E-10 | TRUE |
| ukb-b-4616 | rs2284016 | C | T | 0.399515 | 0.0070943 | 0.001251 | 22 | 1.4E-08 | TRUE |

Abbreviations: *β*, the regression coefficient based on the carnitine effect allele; EA, effect allele; EAF, effect allele frequency; IVs, instrumental variants; NEA, non‐effect allele; SE, standard error; SNP, single‐nucleotide polymorphism.

Table S6. Sleep Apnea Genetic IVs

| ID | SNP | EA | NEA | EAF | β | SE | chr | P-value | mr_keep.outcome |
| --- | --- | --- | --- | --- | --- | --- | --- | --- | --- |
| finn-b-G6_SLEEPAPNO_INCLAVO | rs10928560 | T | C | 0.1948 | -0.0874 | 0.0158 | 2 | 3.17E-08 | TRUE |
| finn-b-G6_SLEEPAPNO_INCLAVO | rs4837016 | A | G | 0.4662 | -0.0697 | 0.0125 | 9 | 2.3E-08 | TRUE |
| finn-b-G6_SLEEPAPNO_INCLAVO | rs10507084 | T | C | 0.1794 | 0.1076 | 0.0163 | 12 | 4.07E-11 | TRUE |
| finn-b-G6_SLEEPAPNO_INCLAVO | rs9937053 | A | G | 0.4299 | 0.1021 | 0.0125 | 16 | 4.03E-16 | TRUE |
| finn-b-G6_SLEEPAPNO_INCLAVO | rs142006783 | C | T | 0.03779 | 0.1781 | 0.0327 | 16 | 4.95E-08 | TRUE |

Abbreviations: *β*, the regression coefficient based on the carnitine effect allele; EA, effect allele; EAF, effect allele frequency; IVs, instrumental variants; NEA, non‐effect allele; SE, standard error; SNP, single‐nucleotide polymorphism.

Table S7. Snoring IVs

| ID | SNP | EA | NEA | EAF | β | SE | chr | P-value | mr_keep.outcome |
| --- | --- | --- | --- | --- | --- | --- | --- | --- | --- |
| ebi-a-GCST009760 | rs1416685 | C | G | 0.4125 | 0.0062 | 0.0011 | 1 | 5E-09 | TRUE |
| ebi-a-GCST009760 | rs75144690 | G | T | 0.7078 | -0.006 | 0.0011 | 1 | 2E-08 | TRUE |
| ebi-a-GCST009760 | rs12119849 | A | G | 0.0825 | 0.0123 | 0.0019 | 1 | 4E-11 | TRUE |
| ebi-a-GCST009760 | rs2115855 | G | T | 0.7485 | 0.0064 | 0.0012 | 2 | 4E-08 | TRUE |
| ebi-a-GCST009760 | rs61597598 | A | G | 0.1163 | 0.0119 | 0.0015 | 2 | 5E-15 | TRUE |
| ebi-a-GCST009760 | rs202110996 | ATT | A | 0.3936 | -0.006 | 0.0011 | 3 | 5E-09 | TRUE |
| ebi-a-GCST009760 | rs1374895 | T | C | 0.5547 | -0.006 | 0.0011 | 3 | 4E-10 | TRUE |
| ebi-a-GCST009760 | rs34811474 | A | G | 0.2167 | -0.008 | 0.0012 | 4 | 1E-10 | TRUE |
| ebi-a-GCST009760 | rs2307111 | C | T | 0.6044 | -0.008 | 0.0011 | 5 | 5E-13 | TRUE |
| ebi-a-GCST009760 | rs34732995 | CTA | C | / | -0.006 | 0.001 | 5 | 2E-08 | TRUE |
| ebi-a-GCST009760 | rs4976269 | A | G | 0.341 | -0.007 | 0.0011 | 5 | 9E-10 | TRUE |
| ebi-a-GCST009760 | rs2207944 | C | T | 0.4573 | 0.0059 | 0.0011 | 6 | 2E-08 | TRUE |
| ebi-a-GCST009760 | rs17060460 | G | A | 0.7654 | 0.0071 | 0.0012 | 6 | 1E-08 | TRUE |
| ebi-a-GCST009760 | rs947612 | A | G | 0.7714 | -0.007 | 0.0012 | 6 | 2E-08 | TRUE |
| ebi-a-GCST009760 | rs17151229 | C | G | 0.3539 | 0.0065 | 0.0011 | 7 | 2E-09 | TRUE |
| ebi-a-GCST009760 | rs7829639 | G | A | 0.2972 | 0.0074 | 0.0012 | 8 | 1E-10 | TRUE |
| ebi-a-GCST009760 | rs13251292 | G | A | 0.5855 | 0.0074 | 0.0011 | 8 | 4E-12 | TRUE |
| ebi-a-GCST009760 | rs4744369 | A | T | 0.6044 | 0.0058 | 0.0011 | 9 | 4E-08 | TRUE |
| ebi-a-GCST009760 | rs725861 | G | A | 0.8082 | 0.0091 | 0.0013 | 10 | 1E-11 | TRUE |
| ebi-a-GCST009760 | rs2049045 | C | G | 0.1909 | -0.008 | 0.0013 | 11 | 9E-10 | TRUE |
| ebi-a-GCST009760 | rs11018488 | T | A | 0.6362 | -0.007 | 0.0011 | 11 | 5E-10 | TRUE |
| ebi-a-GCST009760 | rs10878269 | T | C | 0.6501 | 0.0089 | 0.0011 | 12 | 2E-16 | TRUE |
| ebi-a-GCST009760 | rs9583546 | C | G | 0.6312 | 0.0059 | 0.0011 | 13 | 4E-08 | TRUE |
| ebi-a-GCST009760 | rs12429765 | G | A | 0.507 | -0.007 | 0.0011 | 13 | 6E-11 | TRUE |
| ebi-a-GCST009760 | rs592333 | G | A | 0.4423 | 0.0091 | 0.0011 | 13 | 1E-17 | TRUE |
| ebi-a-GCST009760 | rs2664299 | C | T | 0.5855 | -0.008 | 0.0011 | 14 | 1E-12 | TRUE |
| ebi-a-GCST009760 | rs57292959 | T | G | 0.5567 | 0.0069 | 0.0011 | 16 | 5E-11 | TRUE |
| ebi-a-GCST009760 | rs59502288 | GTCATCCA | G | / | 0.0067 | 0.0011 | 16 | 9E-10 | TRUE |
| ebi-a-GCST009760 | rs8069947 | T | C | 0.5119 | -0.007 | 0.0011 | 17 | 3E-10 | TRUE |
| ebi-a-GCST009760 | rs227727 | T | A | 0.4503 | 0.006 | 0.001 | 17 | 1E-08 | TRUE |
| ebi-a-GCST009760 | rs11409890 | TA | T | / | -0.007 | 0.0011 | 17 | 2E-10 | TRUE |
| ebi-a-GCST009760 | rs78608249 | T | C | 0.7256 | 0.0065 | 0.0012 | 17 | 2E-08 | TRUE |
| ebi-a-GCST009760 | rs57222984 | G | A | 0.7346 | 0.0084 | 0.0012 | 17 | 5E-12 | TRUE |
| ebi-a-GCST009760 | rs180110 | A | G | 0.3698 | 0.0068 | 0.0011 | 17 | 2E-10 | TRUE |
| ebi-a-GCST009760 | rs4987719 | T | C | 0.0278 | 0.0162 | 0.0029 | 18 | 4E-08 | TRUE |
| ebi-a-GCST009760 | rs8108822 | T | C | 0.8976 | -0.011 | 0.0018 | 19 | 6E-10 | TRUE |
| ebi-a-GCST009760 | rs6099273 | T | C | 0.7654 | 0.0067 | 0.0012 | 20 | 3E-08 | TRUE |
| ebi-a-GCST009760 | rs6054427 | A | G | 0.6223 | 0.0063 | 0.0011 | 20 | 4E-09 | TRUE |

Abbreviations: *β*, the regression coefficient based on the carnitine effect allele; EA, effect allele; EAF, effect allele frequency; IVs, instrumental variants; NEA, non‐effect allele; SE, standard error; SNP, single‐nucleotide polymorphism.

Table S8. Results of Mendelian Randomization

| Outcome ID | Exposure ID | nSNP | method | SE | p | OR | 95%CI lower | 95%CI upper | FDR adjusted_p |
| --- | --- | --- | --- | --- | --- | --- | --- | --- | --- |
| Major Depressive Disorder | Sleep duration | 56 | MR Egger | 0.575 | 0.866 | 0.907 | 0.294 | 2.801 | 0.989 |
|  |  |  | Inverse variance weighted | 0.158 | 0.322 | 0.855 | 0.627 | 1.166 | 0.564 |
|  |  |  | Inverse variance weighted (fixed effects) | 0.103 | 0.126 | 0.855 | 0.699 | 1.045 | 0.238 |
|  |  |  | Maximum likelihood | 0.106 | 0.118 | 0.848 | 0.689 | 1.043 | 0.229 |
|  |  |  | Penalised weighted median | 0.172 | 0.445 | 0.877 | 0.625 | 1.229 | 0.584 |
|  |  |  | Simple mode | 0.419 | 0.225 | 0.598 | 0.263 | 1.36 | 0.639 |
|  |  |  | Weighted median | 0.177 | 0.491 | 0.885 | 0.626 | 1.252 | 0.607 |
|  |  |  | Weighted mode | 0.401 | 0.45 | 0.737 | 0.336 | 1.617 | 0.748 |
| Major Depressive Disorder | Insomnia | 32 | MR Egger | 0.607 | 0.871 | 0.905 | 0.275 | 2.978 | 0.989 |
|  |  |  | Inverse variance weighted | 0.207 | 0.001 | 2.017 | 1.344 | 3.026 | 0.011 |
|  |  |  | Inverse variance weighted (fixed effects) | 0.141 | 0.000 | 2.017 | 1.529 | 2.66 | 0.000 |
|  |  |  | Maximum likelihood | 0.146 | 0.000 | 2.052 | 1.541 | 2.731 | 0.000 |
|  |  |  | Penalised weighted median | 0.23 | 0.109 | 1.446 | 0.921 | 2.271 | 0.229 |
|  |  |  | Simple mode | 0.593 | 0.003 | 6.919 | 2.163 | 22.137 | 0.063 |
|  |  |  | Weighted median | 0.232 | 0.112 | 1.446 | 0.918 | 2.279 | 0.235 |
|  |  |  | Weighted mode | 0.356 | 0.429 | 1.33 | 0.662 | 2.674 | 0.748 |
| Major Depressive Disorder | Sleep apnea | 5 | MR Egger | 0.466 | 0.37 | 1.632 | 0.655 | 4.069 | 0.943 |
|  |  |  | Inverse variance weighted | 0.094 | 0.692 | 1.038 | 0.863 | 1.249 | 0.855 |
|  |  |  | Inverse variance weighted (fixed effects) | 0.054 | 0.485 | 1.038 | 0.935 | 1.153 | 0.637 |
|  |  |  | Maximum likelihood | 0.056 | 0.468 | 1.041 | 0.934 | 1.161 | 0.639 |
|  |  |  | Penalised weighted median | 0.072 | 0.026 | 1.173 | 1.019 | 1.349 | 0.078 |
|  |  |  | Simple mode | 0.149 | 0.504 | 1.115 | 0.833 | 1.492 | 0.756 |
|  |  |  | Weighted median | 0.077 | 0.066 | 1.152 | 0.991 | 1.338 | 0.154 |
|  |  |  | Weighted mode | 0.076 | 0.122 | 1.161 | 1 | 1.348 | 0.521 |
| Major Depressive Disorder | Chronotype | 133 | MR Egger | 0.204 | 0.657 | 1.095 | 0.734 | 1.635 | 0.989 |
|  |  |  | Inverse variance weighted | 0.066 | 0.247 | 1.079 | 0.949 | 1.228 | 0.564 |
|  |  |  | Inverse variance weighted (fixed effects) | 0.05 | 0.131 | 1.079 | 0.977 | 1.191 | 0.238 |
|  |  |  | Maximum likelihood | 0.051 | 0.128 | 1.081 | 0.978 | 1.196 | 0.229 |
|  |  |  | Penalised weighted median | 0.08 | 0.264 | 1.094 | 0.934 | 1.281 | 0.381 |
|  |  |  | Simple mode | 0.263 | 0.808 | 0.938 | 0.56 | 1.572 | 0.899 |
|  |  |  | Weighted median | 0.083 | 0.341 | 1.083 | 0.919 | 1.275 | 0.477 |
|  |  |  | Weighted mode | 0.202 | 0.92 | 1.02 | 0.686 | 1.517 | 0.987 |
| Major Depressive Disorder | Daytime dozing | 28 | MR Egger | 1.233 | 0.267 | 4.053 | 0.362 | 45.379 | 0.943 |
|  |  |  | Inverse variance weighted | 0.281 | 0.546 | 1.185 | 0.683 | 2.054 | 0.835 |
|  |  |  | Inverse variance weighted (fixed effects) | 0.218 | 0.436 | 1.185 | 0.773 | 1.815 | 0.637 |
|  |  |  | Maximum likelihood | 0.222 | 0.426 | 1.193 | 0.772 | 1.843 | 0.639 |
|  |  |  | Penalised weighted median | 0.343 | 0.89 | 1.048 | 0.535 | 2.055 | 0.948 |
|  |  |  | Simple mode | 0.77 | 0.91 | 1.092 | 0.241 | 4.942 | 0.910 |
|  |  |  | Weighted median | 0.338 | 0.816 | 1.082 | 0.558 | 2.097 | 0.902 |
|  |  |  | Weighted mode | 0.637 | 0.987 | 1.01 | 0.29 | 3.523 | 0.987 |
| Major Depressive Disorder | Napping during the day | 83 | MR Egger | 0.557 | 0.127 | 2.357 | 0.792 | 7.015 | 0.943 |
|  |  |  | Inverse variance weighted | 0.154 | 0 | 1.807 | 1.336 | 2.443 | 0.000 |
|  |  |  | Inverse variance weighted (fixed effects) | 0.107 | 0 | 1.807 | 1.464 | 2.23 | 0.000 |
|  |  |  | Maximum likelihood | 0.11 | 0 | 1.839 | 1.482 | 2.282 | 0.000 |
|  |  |  | Penalised weighted median | 0.174 | 0.001 | 1.759 | 1.252 | 2.472 | 0.007 |
|  |  |  | Simple mode | 0.436 | 0.25 | 1.656 | 0.705 | 3.888 | 0.639 |
|  |  |  | Weighted median | 0.176 | 0.001 | 1.772 | 1.255 | 2.502 | 0.021 |
|  |  |  | Weighted mode | 0.371 | 0.218 | 1.585 | 0.765 | 3.281 | 0.546 |
| Major Depressive Disorder | Snoring | 23 | MR Egger | 1.699 | 0.218 | 0.115 | 0.004 | 3.229 | 0.943 |
|  |  |  | Inverse variance weighted | 0.341 | 0.599 | 0.836 | 0.428 | 1.632 | 0.835 |
|  |  |  | Inverse variance weighted (fixed effects) | 0.253 | 0.478 | 0.836 | 0.509 | 1.372 | 0.637 |
|  |  |  | Maximum likelihood | 0.259 | 0.505 | 0.842 | 0.507 | 1.397 | 0.639 |
|  |  |  | Penalised weighted median | 0.385 | 0.017 | 0.398 | 0.187 | 0.846 | 0.071 |
|  |  |  | Simple mode | 0.712 | 0.157 | 0.352 | 0.087 | 1.421 | 0.620 |
|  |  |  | Weighted median | 0.382 | 0.039 | 0.455 | 0.215 | 0.96 | 0.117 |
|  |  |  | Weighted mode | 0.667 | 0.124 | 0.344 | 0.093 | 1.272 | 0.521 |
| Schizophrenia | Sleep duration | 57 | MR Egger | 1.142 | 0.797 | 0.745 | 0.079 | 6.978 | 0.989 |
|  |  |  | Inverse variance weighted | 0.292 | 0.007 | 2.2 | 1.241 | 3.901 | 0.049 |
|  |  |  | Inverse variance weighted (fixed effects) | 0.138 | 0 | 2.2 | 1.678 | 2.884 | 0.000 |
|  |  |  | Maximum likelihood | 0.147 | 0 | 2.362 | 1.771 | 3.149 | 0.000 |
|  |  |  | Penalised weighted median | 0.271 | 0.026 | 1.825 | 1.074 | 3.103 | 0.078 |
|  |  |  | Simple mode | 0.675 | 0.881 | 1.106 | 0.295 | 4.157 | 0.910 |
|  |  |  | Weighted median | 0.272 | 0.023 | 1.852 | 1.087 | 3.157 | 0.117 |
|  |  |  | Weighted mode | 0.409 | 0.229 | 1.643 | 0.738 | 3.66 | 0.546 |
| Schizophrenia | Insomnia | 34 | MR Egger | 1.305 | 0.919 | 0.875 | 0.068 | 11.287 | 0.989 |
|  |  |  | Inverse variance weighted | 0.406 | 0.988 | 1.006 | 0.454 | 2.231 | 0.988 |
|  |  |  | Inverse variance weighted (fixed effects) | 0.186 | 0.973 | 1.006 | 0.699 | 1.448 | 0.973 |
|  |  |  | Maximum likelihood | 0.196 | 0.973 | 1.007 | 0.686 | 1.478 | 0.973 |
|  |  |  | Penalised weighted median | 0.315 | 0.014 | 0.459 | 0.248 | 0.852 | 0.071 |
|  |  |  | Simple mode | 0.533 | 0.177 | 0.48 | 0.169 | 1.363 | 0.620 |
|  |  |  | Weighted median | 0.308 | 0.033 | 0.519 | 0.284 | 0.948 | 0.117 |
|  |  |  | Weighted mode | 0.366 | 0.03 | 0.436 | 0.213 | 0.894 | 0.347 |
| Schizophrenia | Sleep apnea | 5 | MR Egger | 0.41 | 0.436 | 1.444 | 0.646 | 3.225 | 0.943 |
|  |  |  | Inverse variance weighted | 0.081 | 0.855 | 1.015 | 0.865 | 1.191 | 0.908 |
|  |  |  | Inverse variance weighted (fixed effects) | 0.07 | 0.83 | 1.015 | 0.886 | 1.163 | 0.908 |
|  |  |  | Maximum likelihood | 0.071 | 0.828 | 1.015 | 0.884 | 1.166 | 0.907 |
|  |  |  | Penalised weighted median | 0.09 | 0.948 | 1.006 | 0.843 | 1.2 | 0.948 |
|  |  |  | Simple mode | 0.178 | 0.313 | 1.227 | 0.866 | 1.739 | 0.657 |
|  |  |  | Weighted median | 0.089 | 0.948 | 1.006 | 0.845 | 1.198 | 0.948 |
|  |  |  | Weighted mode | 0.108 | 0.429 | 0.909 | 0.735 | 1.124 | 0.748 |
| Schizophrenia | Chronotype | 142 | MR Egger | 0.432 | 0.573 | 1.277 | 0.547 | 2.979 | 0.989 |
|  |  |  | Inverse variance weighted | 0.137 | 0.125 | 1.233 | 0.943 | 1.613 | 0.328 |
|  |  |  | Inverse variance weighted (fixed effects) | 0.067 | 0.002 | 1.233 | 1.083 | 1.405 | 0.007 |
|  |  |  | Maximum likelihood | 0.07 | 0.002 | 1.244 | 1.085 | 1.426 | 0.007 |
|  |  |  | Penalised weighted median | 0.119 | 0.653 | 1.055 | 0.835 | 1.334 | 0.762 |
|  |  |  | Simple mode | 0.357 | 0.483 | 0.778 | 0.387 | 1.566 | 0.756 |
|  |  |  | Weighted median | 0.118 | 0.273 | 1.139 | 0.903 | 1.436 | 0.446 |
|  |  |  | Weighted mode | 0.274 | 0.973 | 0.991 | 0.579 | 1.697 | 0.987 |
| Schizophrenia | Daytime dozing | 23 | MR Egger | 3.127 | 0.681 | 0.272 | 0.001 | 124.846 | 0.989 |
|  |  |  | Inverse variance weighted | 0.667 | 0.025 | 4.439 | 1.201 | 16.407 | 0.088 |
|  |  |  | Inverse variance weighted (fixed effects) | 0.344 | 0 | 4.439 | 2.264 | 8.703 | 0.000 |
|  |  |  | Maximum likelihood | 0.365 | 0 | 4.743 | 2.32 | 9.696 | 0.000 |
|  |  |  | Penalised weighted median | 0.501 | 0.043 | 2.758 | 1.032 | 7.367 | 0.100 |
|  |  |  | Simple mode | 0.786 | 0.274 | 2.416 | 0.518 | 11.281 | 0.639 |
|  |  |  | Weighted median | 0.492 | 0.038 | 2.783 | 1.06 | 7.305 | 0.117 |
|  |  |  | Weighted mode | 0.72 | 0.234 | 2.416 | 0.589 | 9.912 | 0.546 |
| Schizophrenia | Napping during the day | 82 | MR Egger | 1.208 | 0.881 | 1.199 | 0.112 | 12.785 | 0.989 |
|  |  |  | Inverse variance weighted | 0.328 | 0.023 | 2.111 | 1.109 | 4.018 | 0.088 |
|  |  |  | Inverse variance weighted (fixed effects) | 0.147 | 0 | 2.111 | 1.581 | 2.818 | 0.000 |
|  |  |  | Maximum likelihood | 0.157 | 0 | 2.196 | 1.615 | 2.985 | 0.000 |
|  |  |  | Penalised weighted median | 0.279 | 0.001 | 2.45 | 1.417 | 4.238 | 0.007 |
|  |  |  | Simple mode | 0.804 | 0.163 | 3.097 | 0.641 | 14.971 | 0.620 |
|  |  |  | Weighted median | 0.28 | 0.006 | 2.17 | 1.254 | 3.756 | 0.042 |
|  |  |  | Weighted mode | 0.705 | 0.052 | 4.027 | 1.011 | 16.034 | 0.364 |
| Schizophrenia | Snoring | 23 | MR Egger | 3.329 | 0.721 | 0.299 | 0 | 203.925 | 0.989 |
|  |  |  | Inverse variance weighted | 0.643 | 0.301 | 0.514 | 0.146 | 1.813 | 0.564 |
|  |  |  | Inverse variance weighted (fixed effects) | 0.339 | 0.05 | 0.514 | 0.265 | 0.999 | 0.117 |
|  |  |  | Maximum likelihood | 0.354 | 0.062 | 0.517 | 0.259 | 1.035 | 0.145 |
|  |  |  | Penalised weighted median | 0.533 | 0.254 | 0.544 | 0.192 | 1.547 | 0.381 |
|  |  |  | Simple mode | 0.862 | 0.497 | 0.552 | 0.102 | 2.989 | 0.756 |
|  |  |  | Weighted median | 0.532 | 0.297 | 0.574 | 0.202 | 1.629 | 0.446 |
|  |  |  | Inverse variance weighted (fixed effects) | 0.339 | 0.050 | 0.514 | 0.265 | 0.999 | 0.117 |
| ADHD | Sleep duration | 52 | MR Egger | 1.091 | 0.272 | 0.298 | 0.035 | 2.525 | 0.943 |
|  |  |  | Inverse variance weighted | 0.206 | 0.022 | 0.625 | 0.417 | 0.934 | 0.088 |
|  |  |  | Inverse variance weighted (fixed effects) | 0.188 | 0.012 | 0.625 | 0.432 | 0.902 | 0.036 |
|  |  |  | Maximum likelihood | 0.191 | 0.018 | 0.637 | 0.438 | 0.926 | 0.054 |
|  |  |  | Penalised weighted median | 0.286 | 0.042 | 0.559 | 0.319 | 0.979 | 0.100 |
|  |  |  | Simple mode | 0.586 | 0.12 | 0.396 | 0.126 | 1.249 | 0.620 |
|  |  |  | Weighted median | 0.281 | 0.045 | 0.569 | 0.328 | 0.987 | 0.118 |
|  |  |  | Weighted mode | 0.639 | 0.154 | 0.396 | 0.113 | 1.387 | 0.539 |
| ADHD | Insomnia | 31 | MR Egger | 1.068 | 0.958 | 1.058 | 0.13 | 8.578 | 0.989 |
|  |  |  | Inverse variance weighted | 0.344 | 0.298 | 1.43 | 0.729 | 2.804 | 0.564 |
|  |  |  | Inverse variance weighted (fixed effects) | 0.24 | 0.136 | 1.43 | 0.893 | 2.289 | 0.238 |
|  |  |  | Maximum likelihood | 0.246 | 0.131 | 1.449 | 0.895 | 2.346 | 0.229 |
|  |  |  | Penalised weighted median | 0.368 | 0.272 | 1.499 | 0.728 | 3.086 | 0.381 |
|  |  |  | Simple mode | 0.672 | 0.36 | 1.868 | 0.501 | 6.967 | 0.687 |
|  |  |  | Weighted median | 0.365 | 0.29 | 1.471 | 0.719 | 3.008 | 0.446 |
|  |  |  | Weighted mode | 0.529 | 0.463 | 1.483 | 0.525 | 4.184 | 0.748 |
| ADHD | Sleep apnea | 4 | MR Egger | 0.526 | 0.371 | 1.826 | 0.652 | 5.115 | 0.943 |
|  |  |  | Inverse variance weighted | 0.088 | 0.865 | 1.015 | 0.854 | 1.206 | 0.908 |
|  |  |  | Inverse variance weighted (fixed effects) | 0.088 | 0.865 | 1.015 | 0.854 | 1.206 | 0.908 |
|  |  |  | Maximum likelihood | 0.089 | 0.864 | 1.015 | 0.854 | 1.208 | 0.907 |
|  |  |  | Penalised weighted median | 0.103 | 0.912 | 1.011 | 0.827 | 1.237 | 0.948 |
|  |  |  | Simple mode | 0.146 | 0.66 | 0.931 | 0.699 | 1.241 | 0.866 |
|  |  |  | Weighted median | 0.104 | 0.913 | 1.011 | 0.825 | 1.24 | 0.948 |
|  |  |  | Weighted mode | 0.123 | 0.715 | 1.051 | 0.825 | 1.337 | 0.903 |
| ADHD | Chronotype | 129 | MR Egger | 0.424 | 0.989 | 0.994 | 0.433 | 2.282 | 0.989 |
|  |  |  | Inverse variance weighted | 0.132 | 0.617 | 0.936 | 0.723 | 1.212 | 0.835 |
|  |  |  | Inverse variance weighted (fixed effects) | 0.085 | 0.44 | 0.936 | 0.792 | 1.106 | 0.637 |
|  |  |  | Maximum likelihood | 0.087 | 0.436 | 0.934 | 0.787 | 1.109 | 0.639 |
|  |  |  | Penalised weighted median | 0.139 | 0.515 | 1.095 | 0.834 | 1.437 | 0.636 |
|  |  |  | Simple mode | 0.401 | 0.705 | 1.164 | 0.53 | 2.554 | 0.871 |
|  |  |  | Weighted median | 0.138 | 0.536 | 1.089 | 0.831 | 1.428 | 0.625 |
|  |  |  | Weighted mode | 0.332 | 0.751 | 1.111 | 0.58 | 2.13 | 0.903 |
| ADHD | Daytime dozing | 30 | MR Egger | 2.441 | 0.291 | 13.821 | 0.116 | 1653.116 | 0.943 |
|  |  |  | Inverse variance weighted | 0.558 | 0.844 | 0.896 | 0.3 | 2.675 | 0.908 |
|  |  |  | Inverse variance weighted (fixed effects) | 0.354 | 0.756 | 0.896 | 0.448 | 1.792 | 0.882 |
|  |  |  | Maximum likelihood | 0.364 | 0.768 | 0.898 | 0.44 | 1.832 | 0.896 |
|  |  |  | Penalised weighted median | 0.588 | 0.224 | 0.489 | 0.155 | 1.55 | 0.381 |
|  |  |  | Simple mode | 1.258 | 0.541 | 0.459 | 0.039 | 5.41 | 0.757 |
|  |  |  | Weighted median | 0.589 | 0.398 | 0.608 | 0.191 | 1.928 | 0.522 |
|  |  |  | Weighted mode | 1.234 | 0.555 | 0.479 | 0.043 | 5.375 | 0.777 |
| ADHD | Napping during the day | 78 | MR Egger | 0.862 | 0.449 | 1.926 | 0.356 | 10.43 | 0.943 |
|  |  |  | Inverse variance weighted | 0.245 | 0.125 | 1.455 | 0.901 | 2.35 | 0.328 |
|  |  |  | Inverse variance weighted (fixed effects) | 0.181 | 0.038 | 1.455 | 1.02 | 2.074 | 0.100 |
|  |  |  | Maximum likelihood | 0.185 | 0.036 | 1.472 | 1.025 | 2.115 | 0.095 |
|  |  |  | Penalised weighted median | 0.278 | 0 | 3.224 | 1.871 | 5.554 | 0.000 |
|  |  |  | Simple mode | 0.661 | 0.043 | 3.9 | 1.068 | 14.247 | 0.452 |
|  |  |  | Weighted median | 0.288 | 0.004 | 2.31 | 1.314 | 4.061 | 0.042 |
|  |  |  | Weighted mode | 0.645 | 0.033 | 4.049 | 1.144 | 14.324 | 0.347 |
| ADHD | Snoring | 29 | MR Egger | 2.758 | 0.167 | 50.475 | 0.226 | 11249.576 | 0.943 |
|  |  |  | Inverse variance weighted | 0.495 | 0.636 | 1.265 | 0.479 | 3.338 | 0.835 |
|  |  |  | Inverse variance weighted (fixed effects) | 0.369 | 0.524 | 1.265 | 0.614 | 2.605 | 0.647 |
|  |  |  | Maximum likelihood | 0.377 | 0.517 | 1.276 | 0.61 | 2.67 | 0.639 |
|  |  |  | Penalised weighted median | 0.584 | 0.18 | 2.185 | 0.696 | 6.862 | 0.344 |
|  |  |  | Simple mode | 1.203 | 0.813 | 1.334 | 0.126 | 14.097 | 0.899 |
|  |  |  | Weighted median | 0.562 | 0.246 | 1.92 | 0.638 | 5.775 | 0.446 |
|  |  |  | Weighted mode | 1.26 | 0.774 | 1.441 | 0.122 | 17.033 | 0.903 |

Abbreviation: MR, Mendelian randomization; ADHD, Attention-Deficit / Hyperactivity Disorder.

Table S9. Sensitivity analysis for causal effect of sleep on psychiatric disorders

| outcome | exposure | MR Egger | | | IVW | | |
| --- | --- | --- | --- | --- | --- | --- | --- |
|  |  | Q | Q_df | Q_pval | Q | Q_df | Q_pval |
| Major Depressive Disorder | Sleep duration | 130.7916 | 54 | 2.57666E-08 | 130.8198 | 55 | 4.0431E-08 |
|  | Insomnia | 62.5061 | 30 | 0.000453047 | 66.58675 | 31 | 0.00021064 |
|  | Sleep apnea | 9.356878 | 3 | 0.02490371 | 12.423 | 4 | 0.01446792 |
|  | Chronotype | 224.3044 | 131 | 7.19514E-07 | 224.3146 | 132 | 9.5055E-07 |
|  | Daytime dozing | 43.14576 | 26 | 0.0186422 | 44.88837 | 27 | 0.01670688 |
|  | Napping during day | 168.5467 | 81 | 4.19622E-08 | 169.0605 | 82 | 5.3473E-08 |
|  | Snoring | 37.54867 | 21 | 0.01454941 | 40.07379 | 22 | 0.0105991 |
| schizophrenia | Sleep duration | 245.906 | 55 | 5.82389E-26 | 250.2134 | 56 | 2.2953E-26 |
|  | Insomnia | 157.832 | 32 | 1.43951E-18 | 157.8948 | 33 | 3.1657E-18 |
|  | Sleep apnea | 4.356251 | 3 | 0.2254764 | 5.475196 | 4 | 0.2419184 |
|  | Chronotype | 595.1614 | 140 | 2.09779E-57 | 595.1916 | 141 | 4.2925E-57 |
|  | Daytime dozing | 79.76776 | 21 | 8.82944E-09 | 82.94166 | 2.20E+01 | 5.2833E-09 |
|  | Napping during day | 400.6075 | 80 | 3.62137E-44 | 401.7951 | 81 | 5.058E-44 |
|  | Snoring | 79.20989 | 21 | 1.09396E-08 | 79.31397 | 22 | 2.1003E-08 |
| ADHD | Sleep duration | 60.60001 | 50 | 0.1448436 | 61.18047 | 51 | 0.1555655 |
|  | Insomnia | 61.25166 | 29 | 0.000430457 | 61.4403 | 30 | 0.00061405 |
|  | Sleep apnea | 0.2524679 | 2 | 0.8814086 | 1.535626 | 3 | 0.6740727 |
|  | Chronotype | 305.575 | 127 | 9.53402E-17 | 305.6287 | 128 | 1.4664E-16 |
|  | Daytime dozing | 68.89582 | 28 | 2.65094E-05 | 72.15559 | 29 | 1.5289E-05 |
|  | Napping during day | 140.5593 | 76 | 9.63212E-06 | 140.7727 | 77 | 1.2659E-05 |
|  | Snoring | 47.27876 | 27 | 0.009231773 | 50.50768 | 28 | 0.00567342 |

Abbreviation: IVW, Inverse variance weighted; ADHD, Attention-Deficit / Hyperactivity Disorder.

Table S10. Pleiotropy analysis for causal effect of sleep on psychiatric disorders

| exposure | outcome | egger_intercept | SE | pvalue |
| --- | --- | --- | --- | --- |
| Sleep duration | Major Depressive Disorder | -0.001 | 0.007 | 0.914 |
| Insomnia |  | 0.010 | 0.007 | 0.172 |
| Sleep apnea |  | -0.042 | 0.042 | 0.395 |
| Chronotype |  | 0.000 | 0.003 | 0.939 |
| Daytime dozing |  | -0.010 | 0.009 | 0.315 |
| Napping during day |  | -0.003 | 0.005 | 0.621 |
| Snoring |  | 0.016 | 0.013 | 0.248 |
| Sleep duration | schizophrenia | 0.013 | 0.013 | 0.331 |
| Insomnia |  | 0.002 | 0.015 | 0.911 |
| Sleep apnea |  | -0.033 | 0.038 | 0.445 |
| Chronotype |  | -0.001 | 0.007 | 0.933 |
| Daytime dozing |  | 0.021 | 0.023 | 0.371 |
| Napping during day |  | 0.006 | 0.011 | 0.628 |
| Snoring |  | 0.004 | 0.026 | 0.870 |
| Sleep duration | ADHD | 0.008 | 0.012 | 0.492 |
| Insomnia |  | 0.004 | 0.012 | 0.767 |
| Sleep apnea |  | -0.053 | 0.047 | 0.375 |
| Chronotype |  | -0.001 | 0.007 | 0.882 |
| Daytime dozing |  | -0.021 | 0.019 | 0.259 |
| Napping during day |  | -0.003 | 0.008 | 0.735 |
| Snoring |  | -0.028 | 0.020 | 0.186 |

Abbreviation: ADHD, Attention-Deficit / Hyperactivity Disorder.

# Supplementary figure


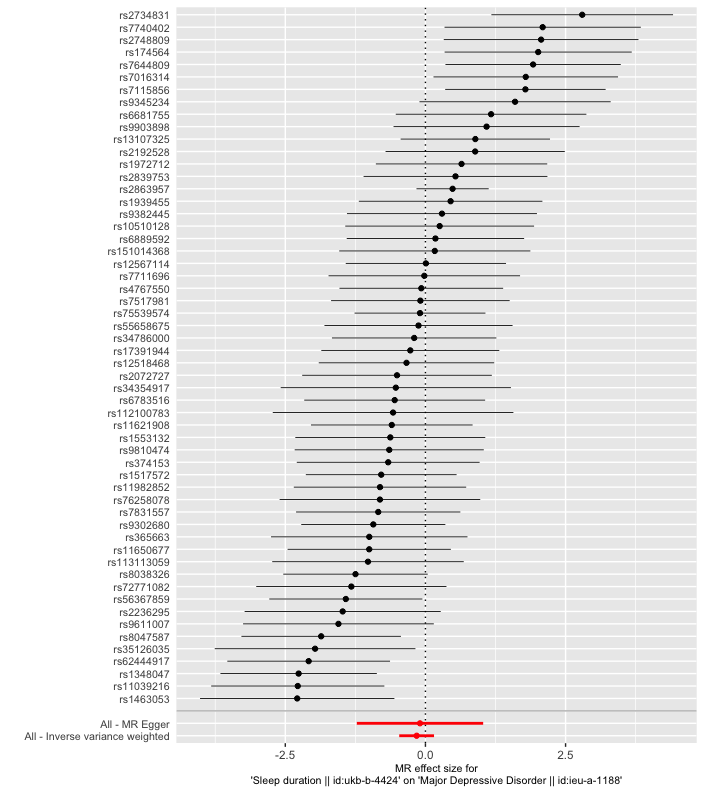


Fig S1. Single single-nucleotide polymorphism (SNP) analysis for individual and combined SNP effects of sleep duration on Major Depressive Disorder.


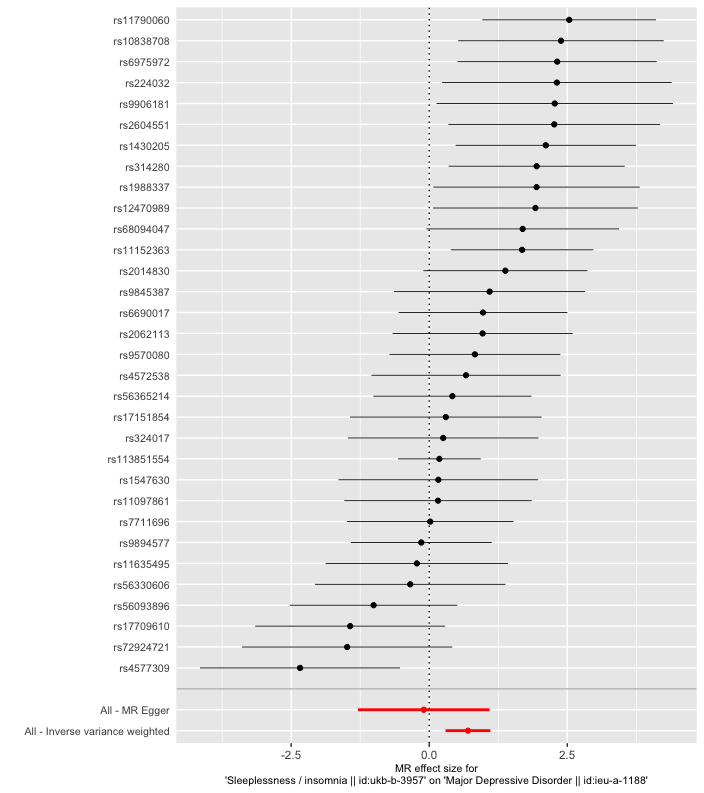


Fig S2. Single single-nucleotide polymorphism (SNP) analysis for individual and combined SNP effects of insomnia on Major Depressive Disorder.


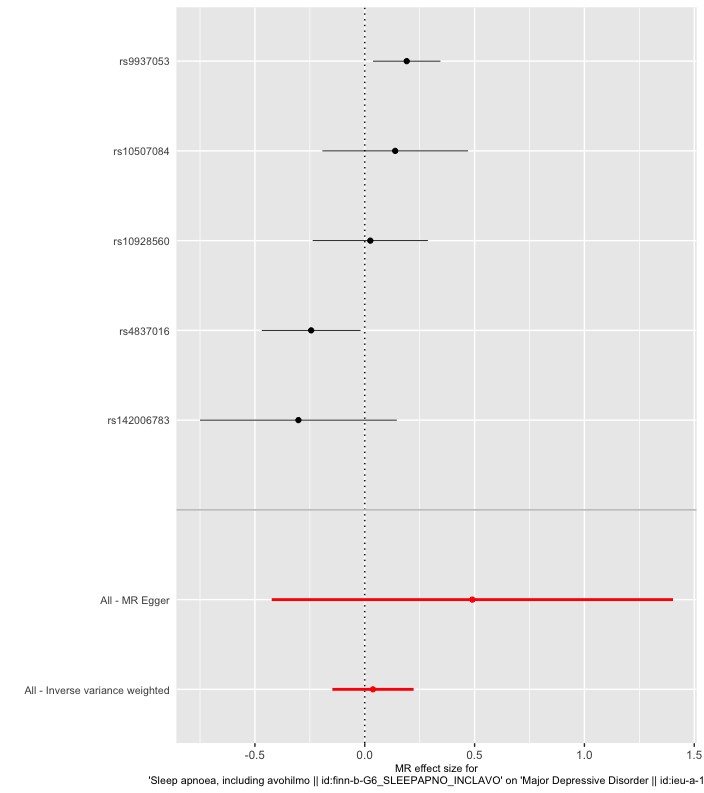


Fig S3. Single single-nucleotide polymorphism (SNP) analysis for individual and combined SNP effects of Sleep apnea on Major Depressive Disorder.


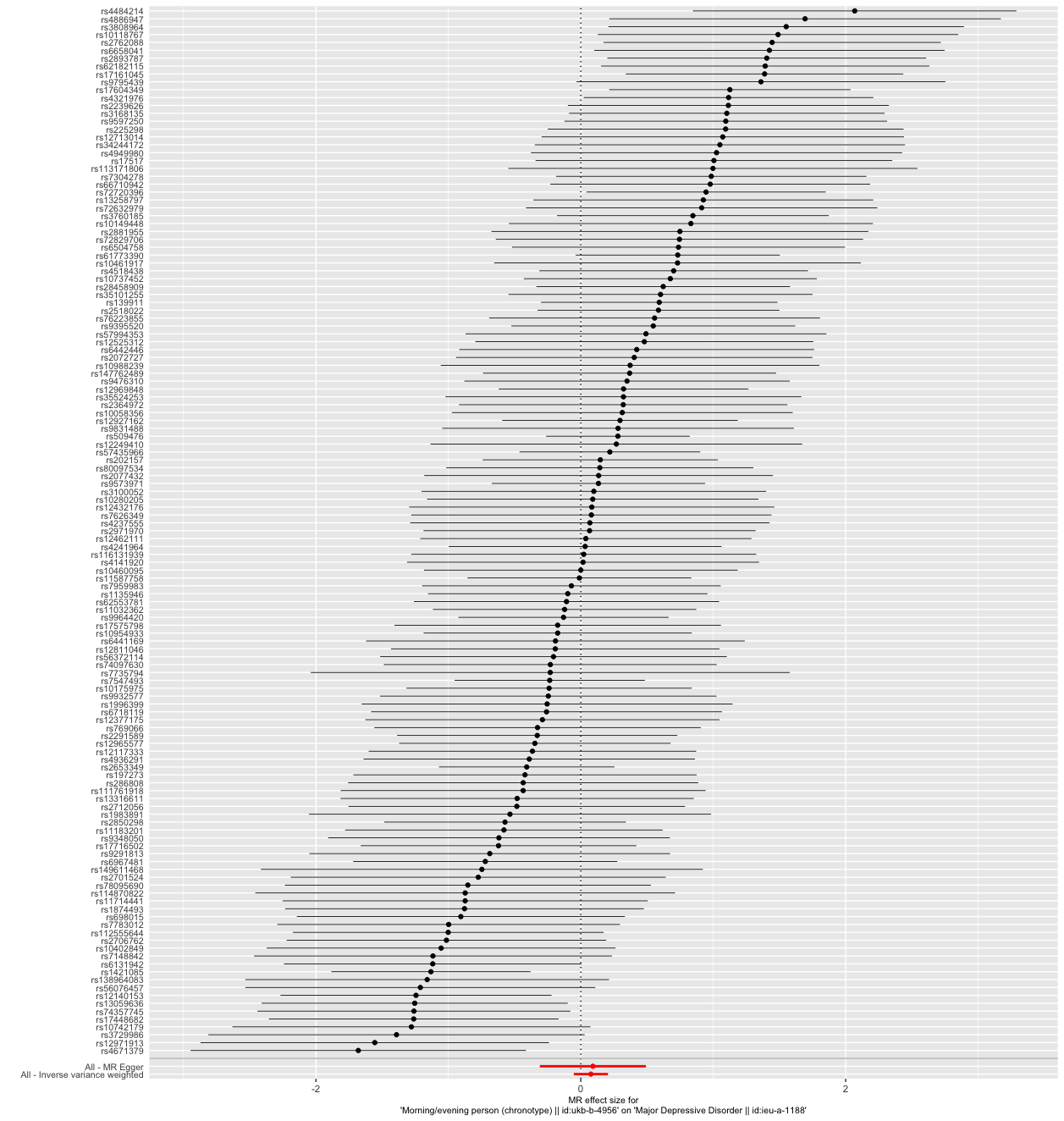


Fig S4. Single single-nucleotide polymorphism (SNP) analysis for individual and combined SNP effects of chronotype on Major Depressive Disorder.


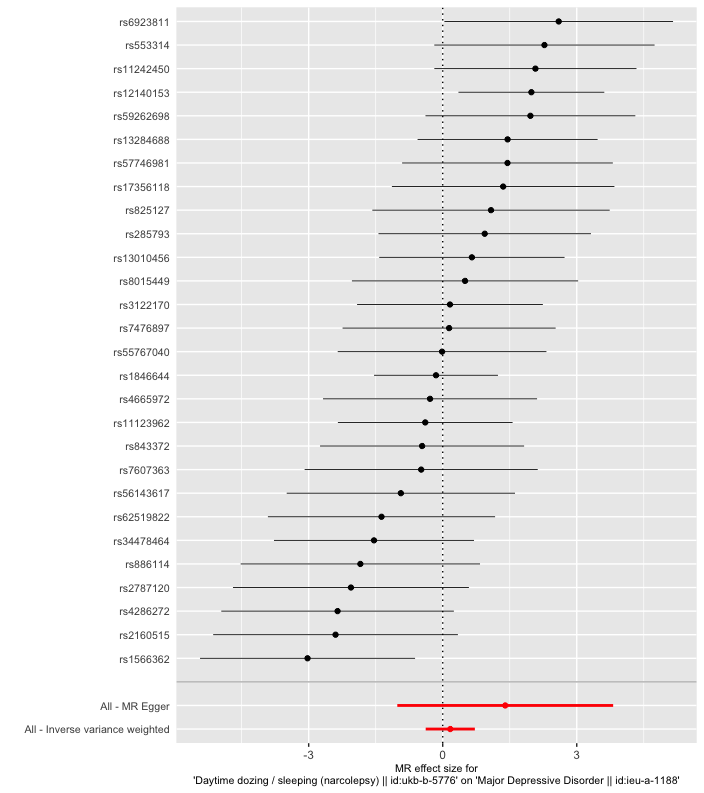


Fig S5. Single single-nucleotide polymorphism (SNP)analysis for individual and combined SNP effects of daytime dozing on Major Depressive Disorder.


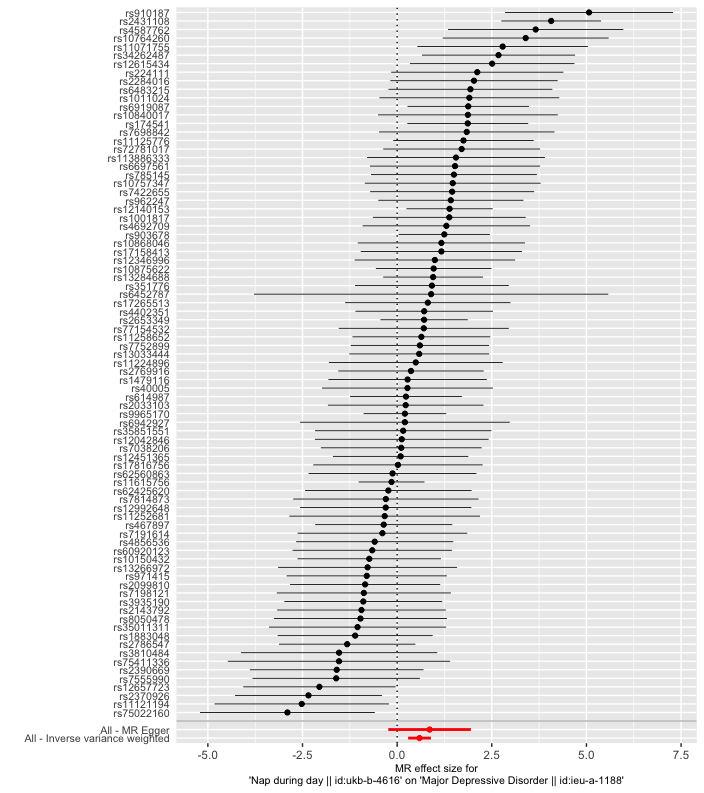


Fig S6. Single single-nucleotide polymorphism (SNP) analysis for individual and combined SNP effects of Napping during the day on Major Depressive Disorder.


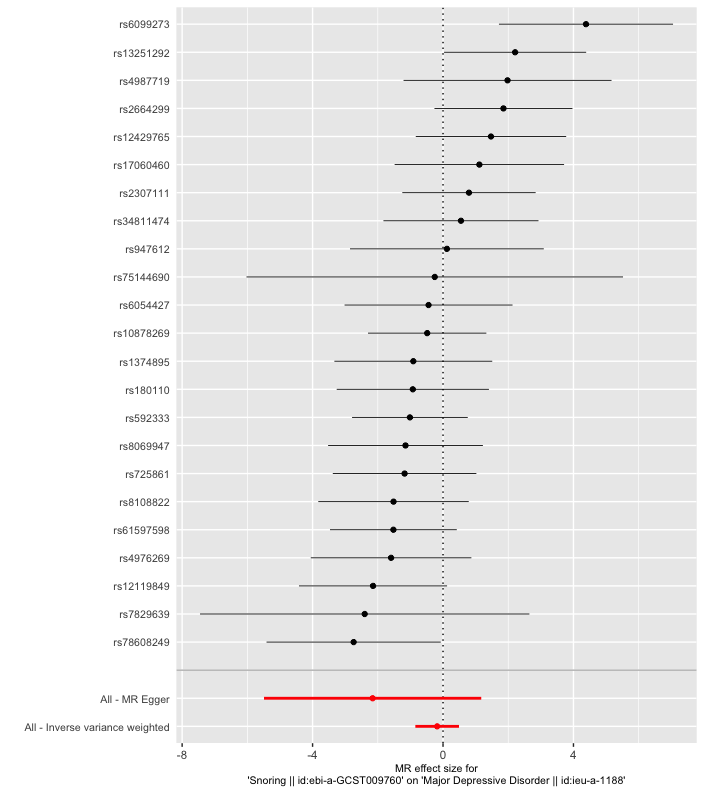


Fig S7. Single single-nucleotide polymorphism (SNP) analysis for individual and combined SNP effects of Snoring on Major Depressive Disorder.


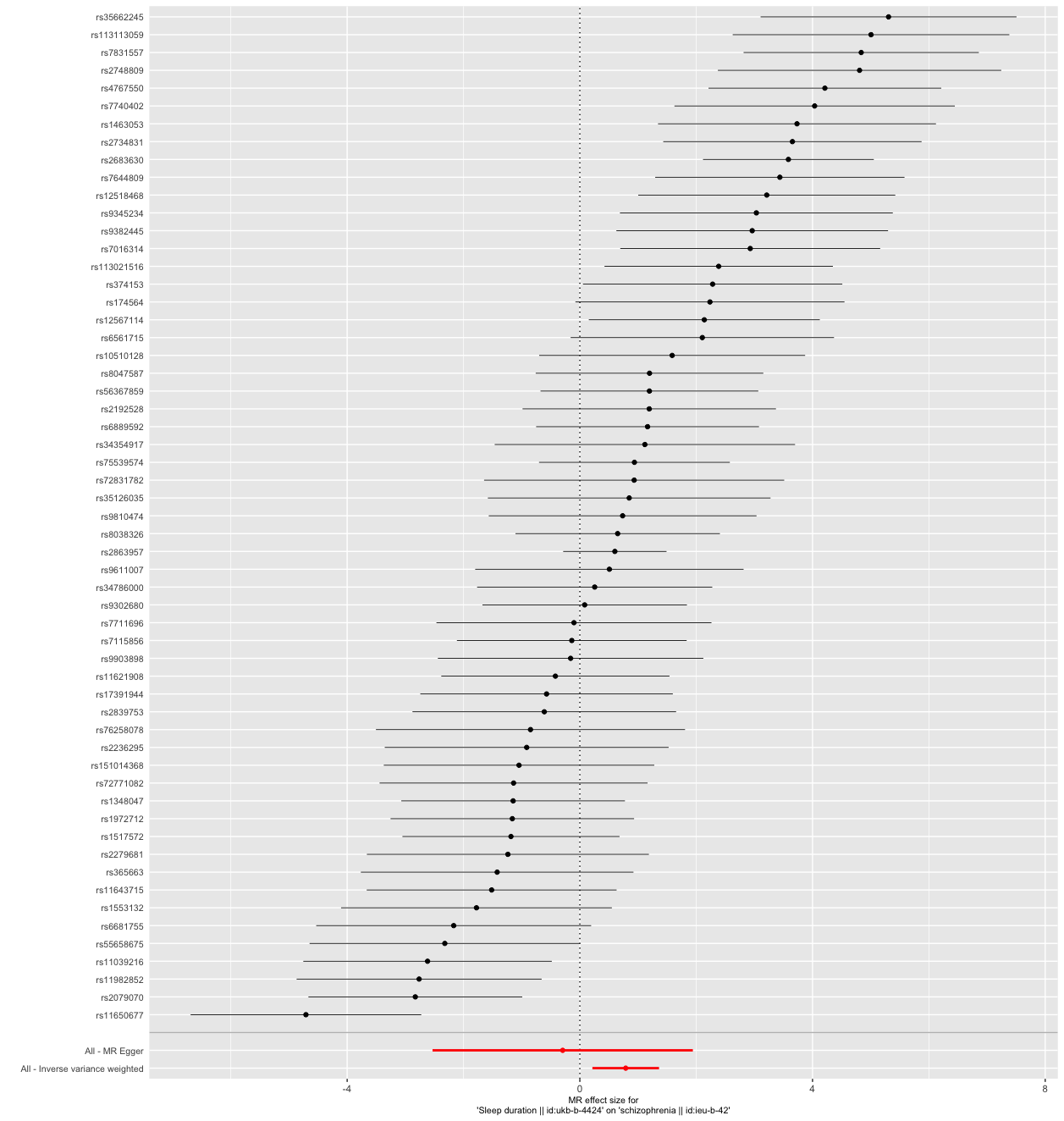


Fig S8. Single single-nucleotide polymorphism (SNP) analysis for individual and combined SNP effects of Sleep duration on schizophrenia.


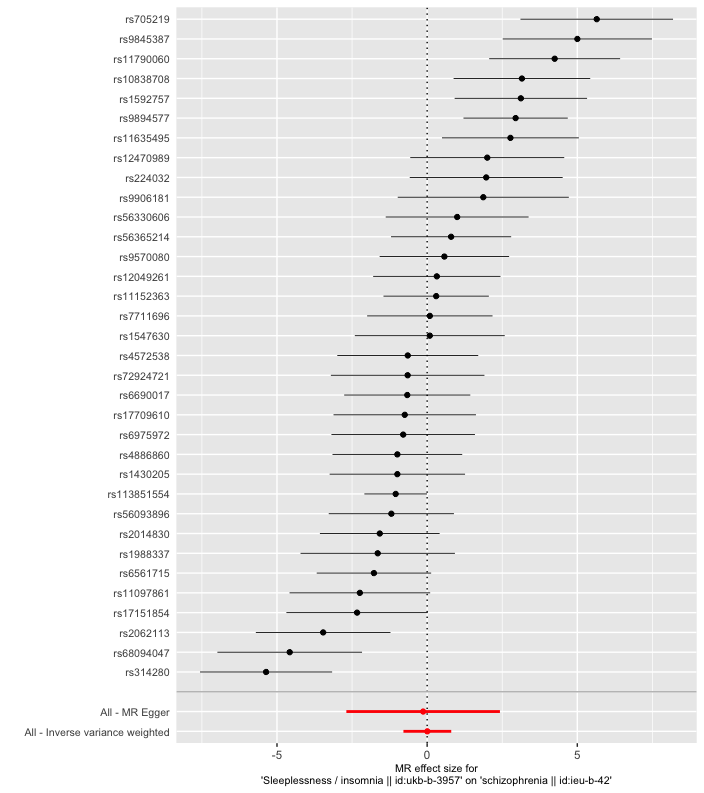


Fig S9. Single single-nucleotide polymorphism (SNP)analysis for individual and combined SNP effects of insomnia on schizophrenia.


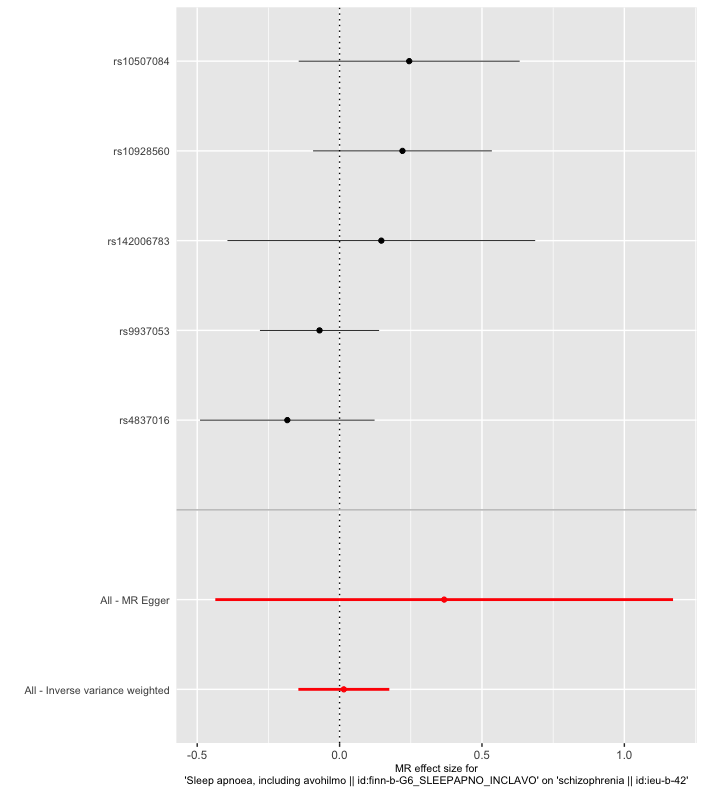


Fig S10. Single single-nucleotide polymorphism (SNP) analysis for individual and combined SNP effects of sleep apnea on schizophrenia.


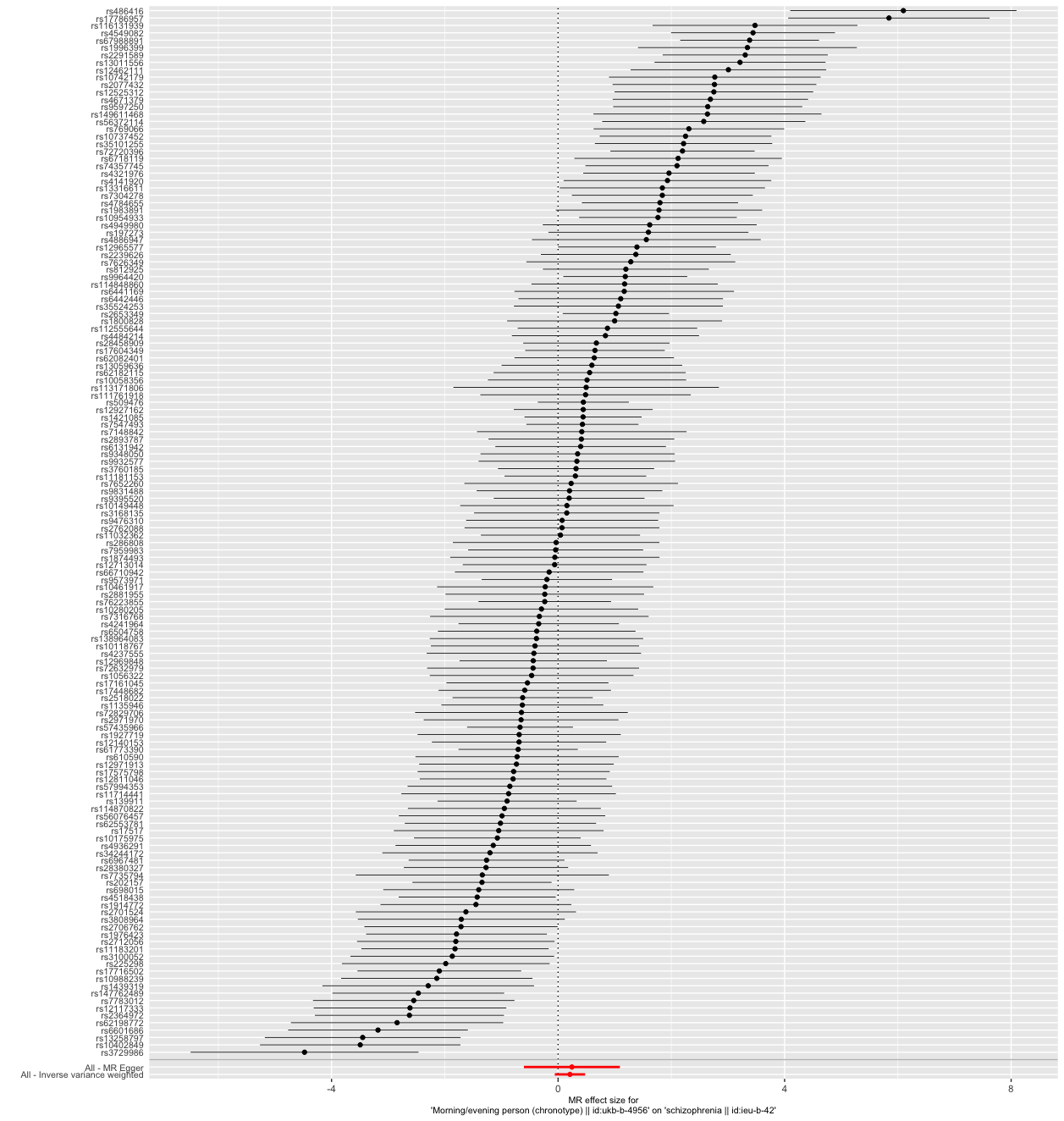


Fig S11. Single single-nucleotide polymorphism (SNP) analysis for individual and combined SNP effects of chronotype on schizophrenia.


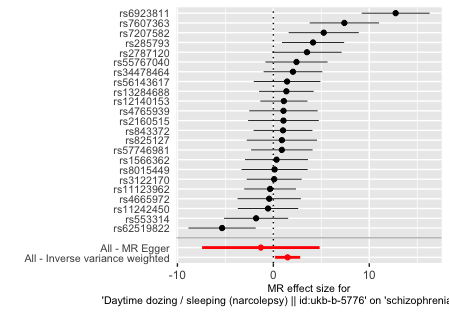


Fig S12. Single single-nucleotide polymorphism (SNP) analysis for individual and combined SNP effects of daytime dozing on schizophrenia.


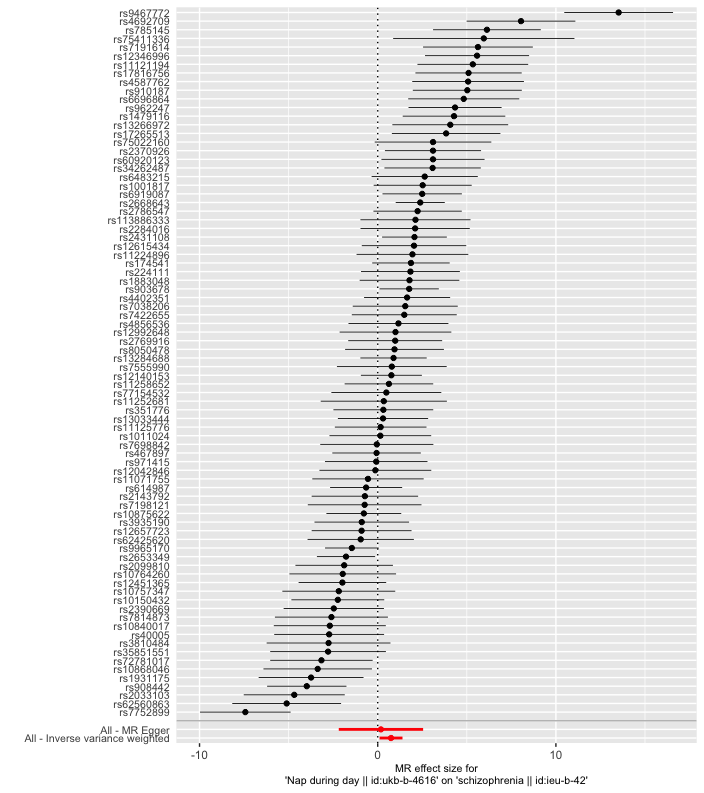


Fig S13. Single single-nucleotide polymorphism (SNP)analysis for individual and combined SNP effects of Napping during the day on schizophrenia.


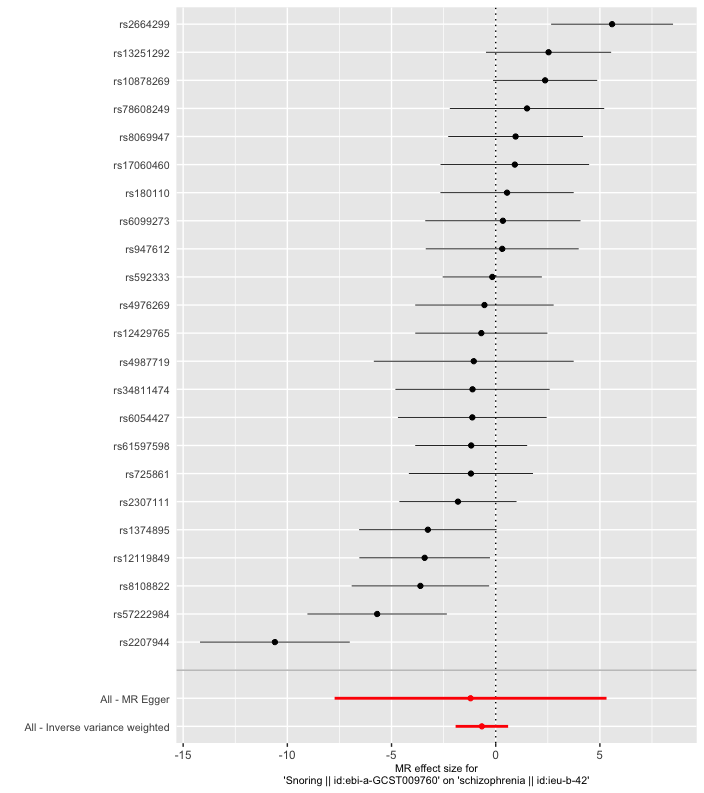


Fig S14. Single single-nucleotide polymorphism (SNP) analysis for individual and combined SNP effects of Snoring on schizophrenia.


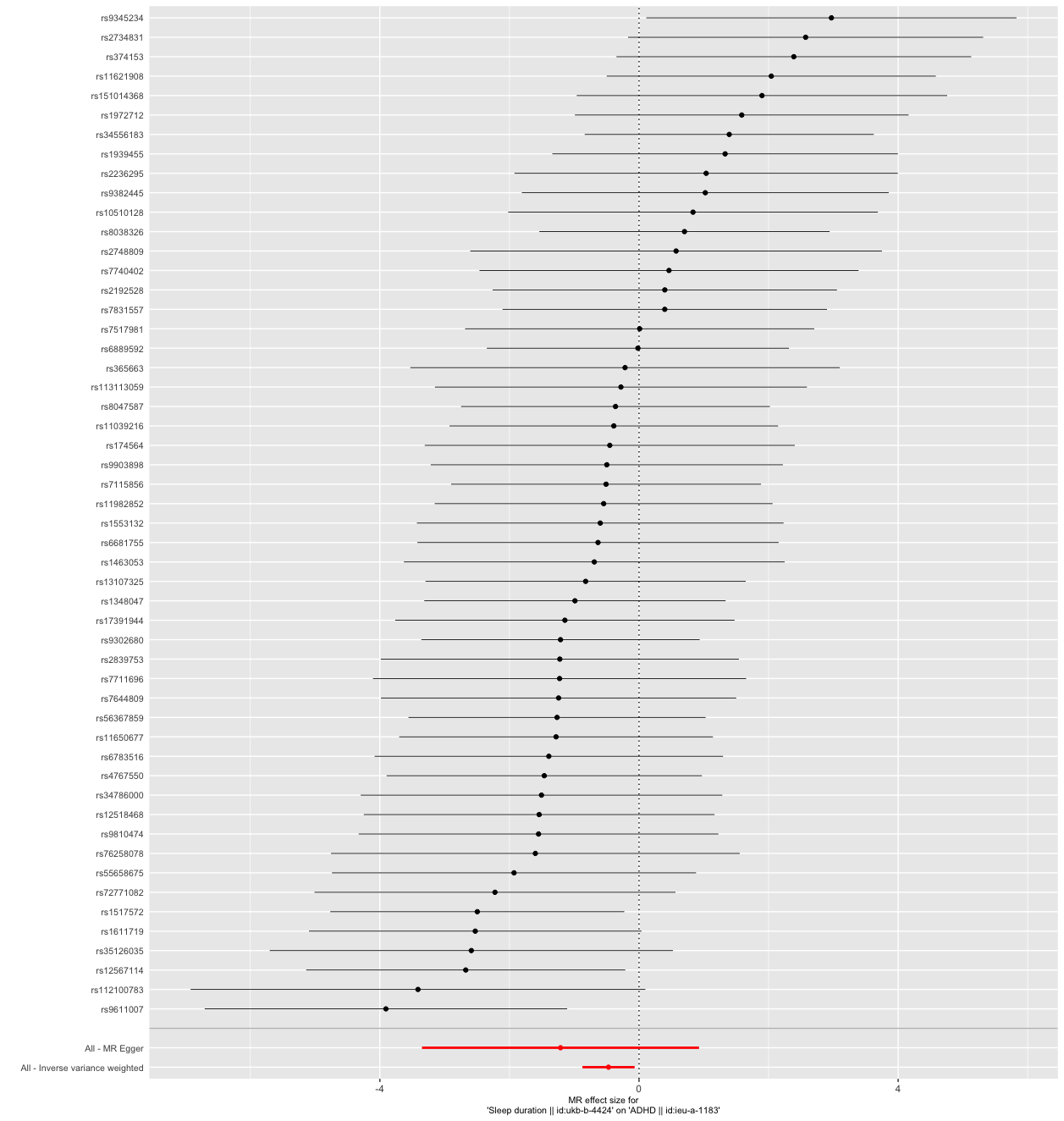


Fig S15 Single single-nucleotide polymorphism (SNP) analysis for individual and combined SNP effects of Sleep duration on Attention-Deficit / Hyperactivity Disorder.


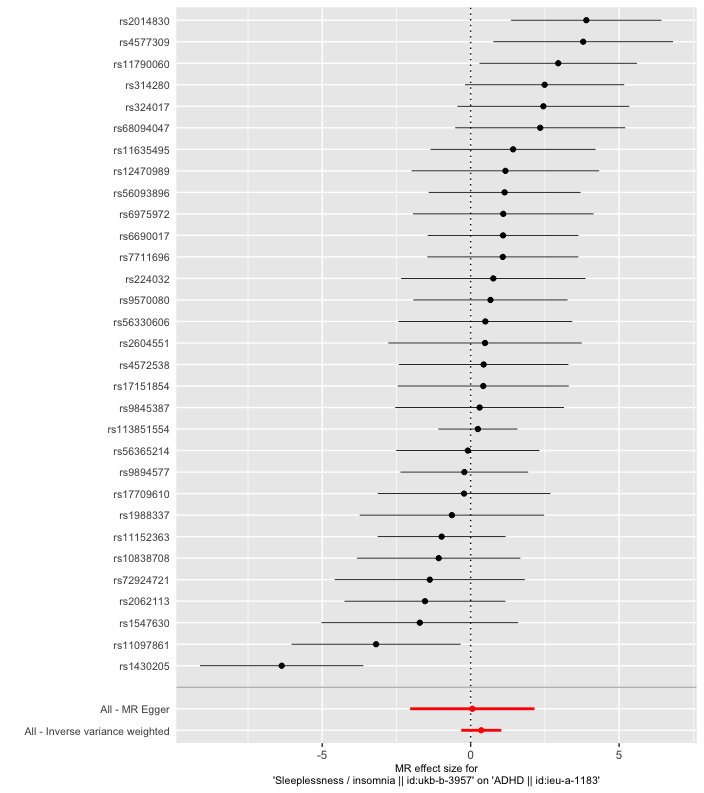


Fig S16. Single single-nucleotide polymorphism (SNP) analysis for individual and combined SNP effects of Insomnia on Attention-Deficit / Hyperactivity Disorder.


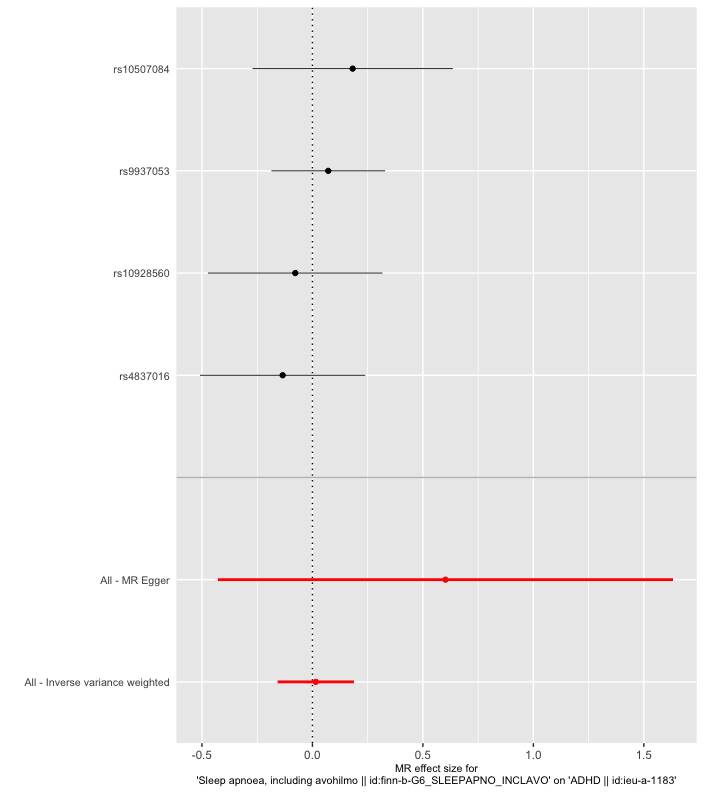


Fig S17. Single single-nucleotide polymorphism (SNP) analysis for individual and combined SNP effects of Sleep apnea on Attention-Deficit / Hyperactivity Disorder.


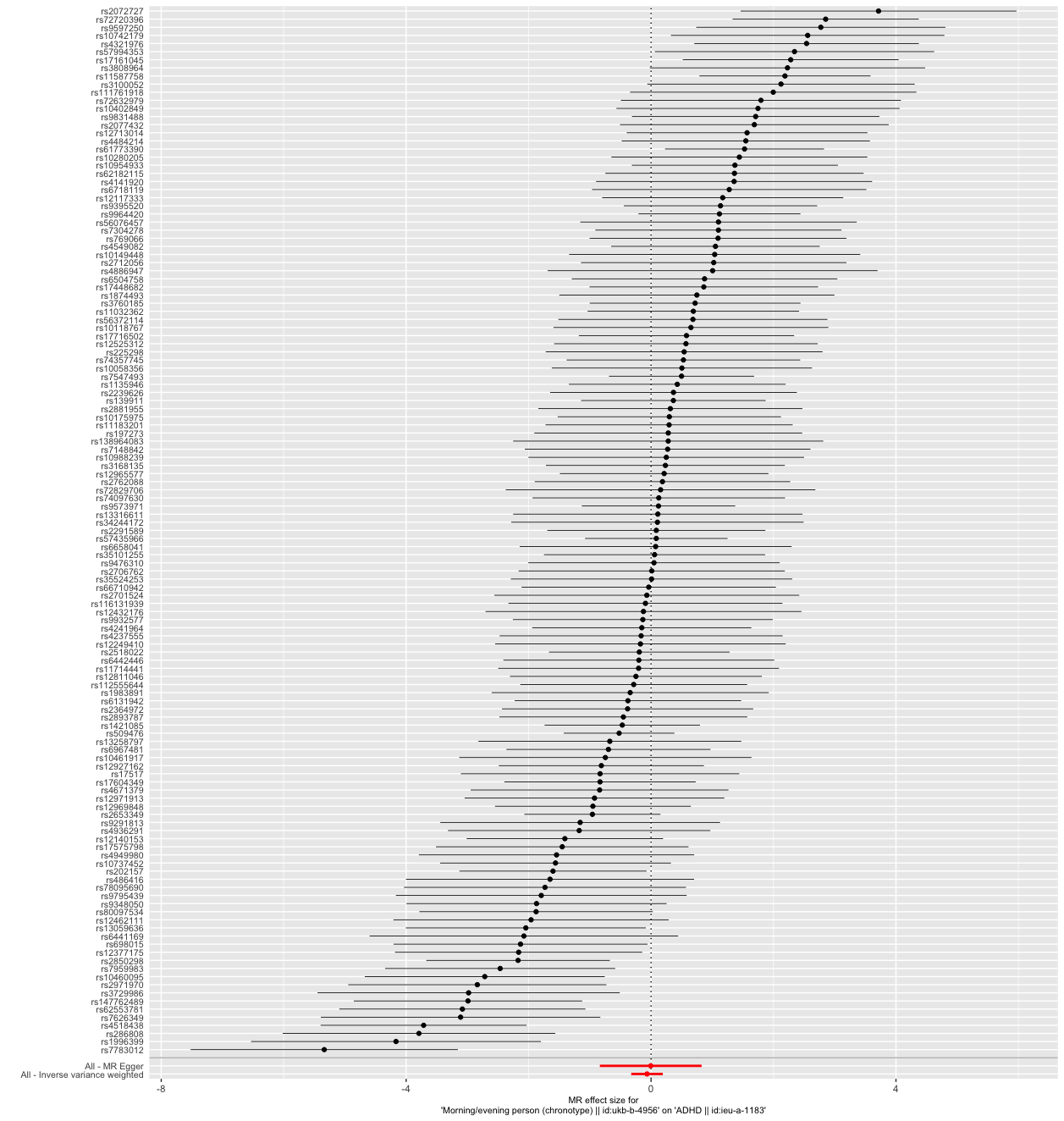


Fig S18. Single single-nucleotide polymorphism (SNP) analysis for individual and combined SNP effects of Chronotype on Attention-Deficit / Hyperactivity Disorder.


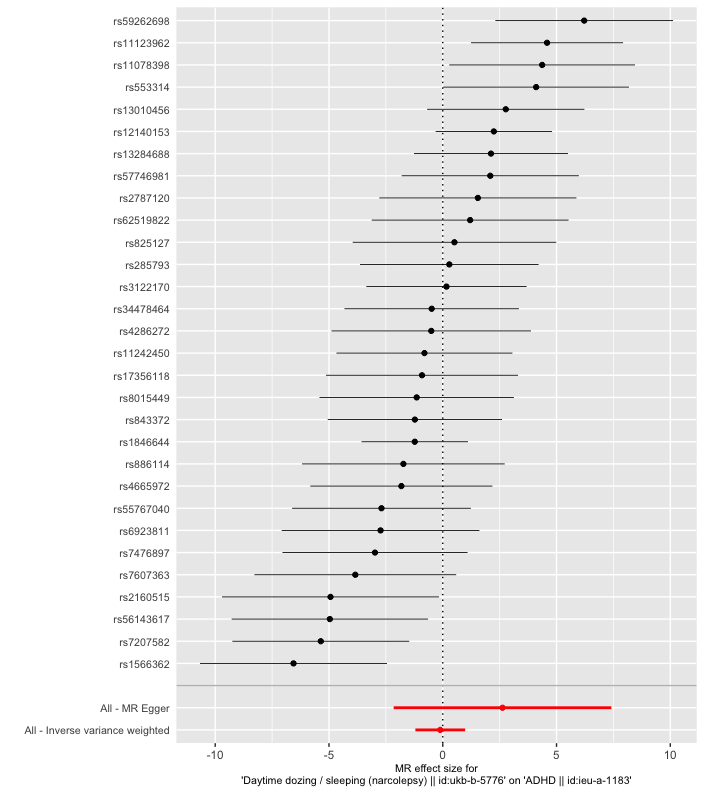


Fig S19. Single single-nucleotide polymorphism (SNP) analysis for individual and combined SNP effects of Daytime dozing on Attention-Deficit / Hyperactivity Disorder.


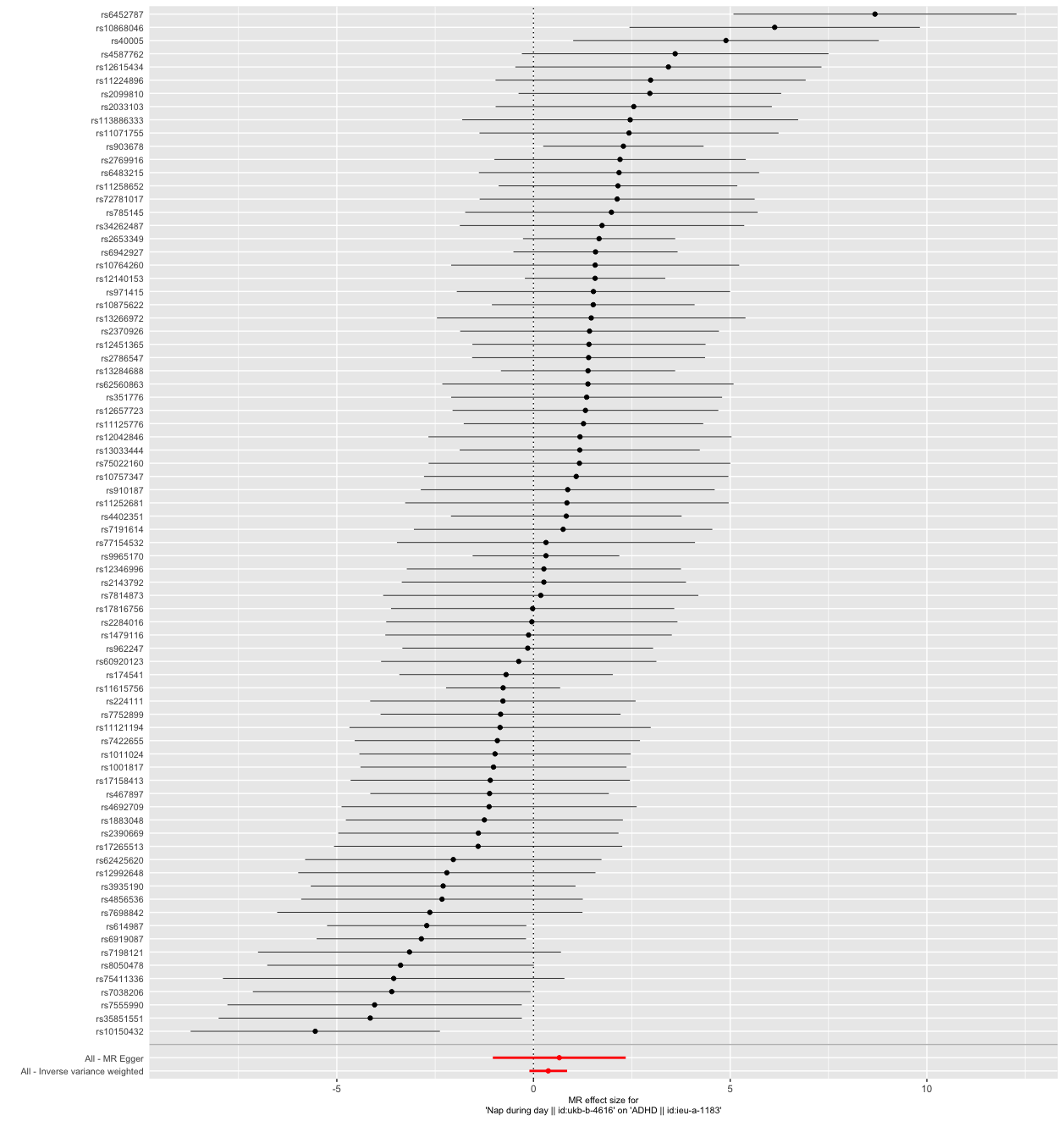


Fig S20. Single single-nucleotide polymorphism (SNP) analysis for individual and combined SNP effects of Napping during the day on Attention-Deficit / Hyperactivity Disorder.


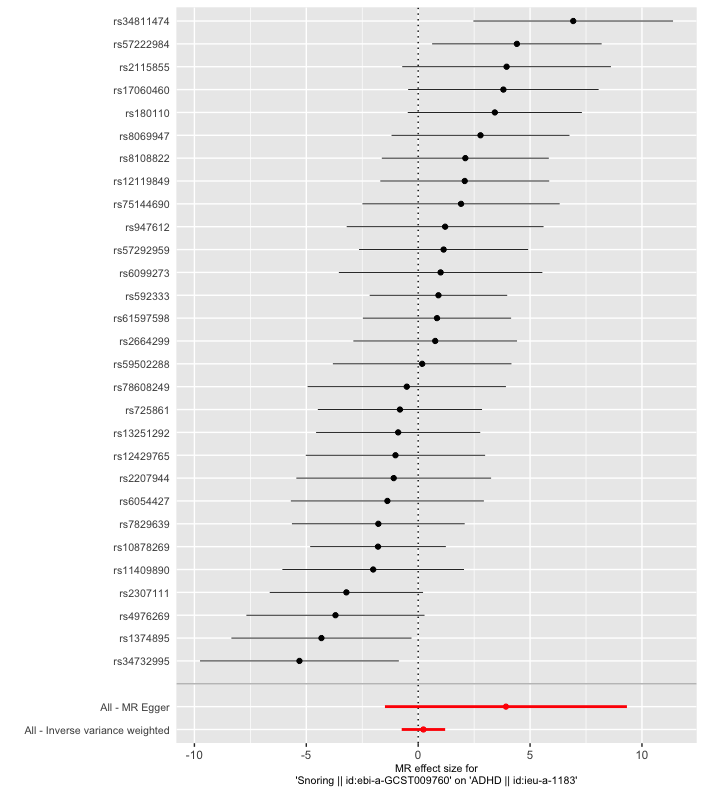


Fig S21. Single single-nucleotide polymorphism (SNP) analysis for individual and combined SNP effects of Snoring on Attention-Deficit / Hyperactivity Disorder.


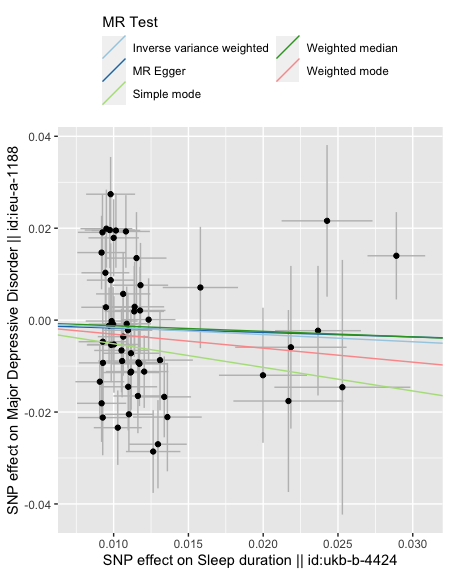


Fig S22. Scatter plot of single-nucleotide polymorphism (SNP) associated with the sleep duration and Major Depressive Disorder.


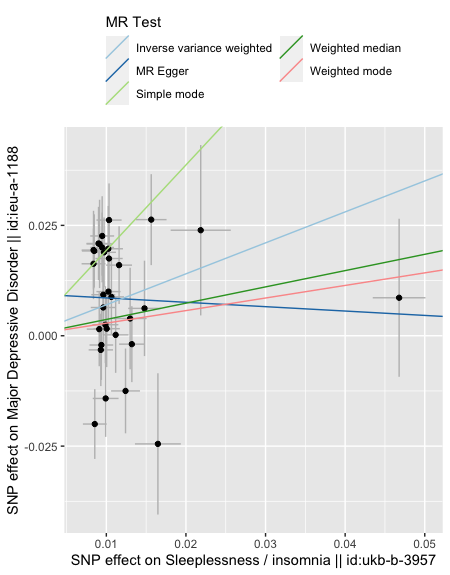


Fig S23. Scatter plot of single-nucleotide polymorphism (SNP) associated with the Insomnia and Major Depressive Disorder.


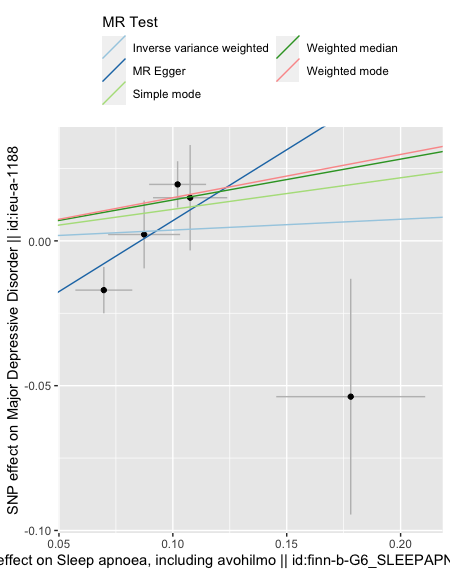


Fig S24. Scatter plot of single-nucleotide polymorphism (SNP) associated with the sleep apnea and Major Depressive Disorder.


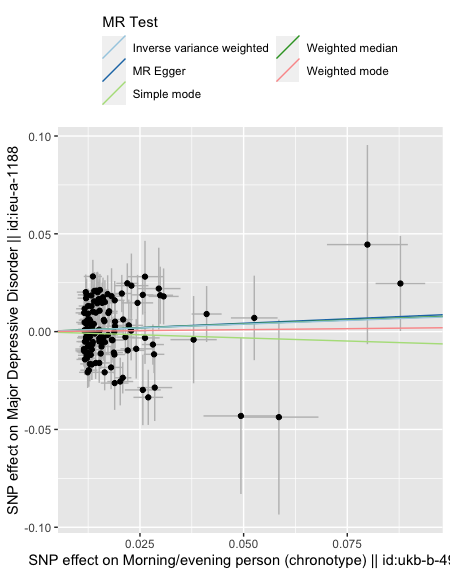


Fig S25. Scatter plot of single-nucleotide polymorphism (SNP) associated with the chronotype and Major Depressive Disorder.


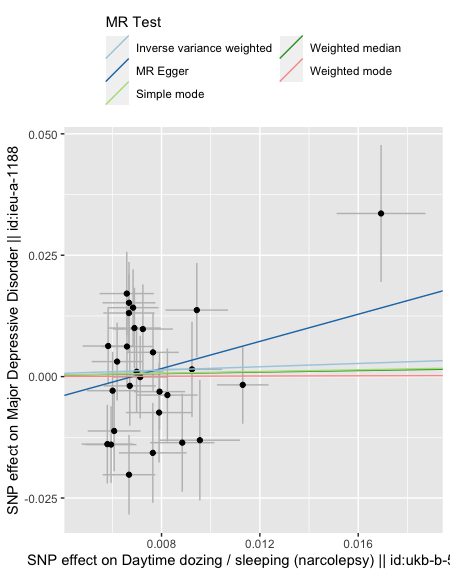


Fig S26. Scatter plot of single-nucleotide polymorphism (SNP) associated with the daytime dozing and Major Depressive Disorder.


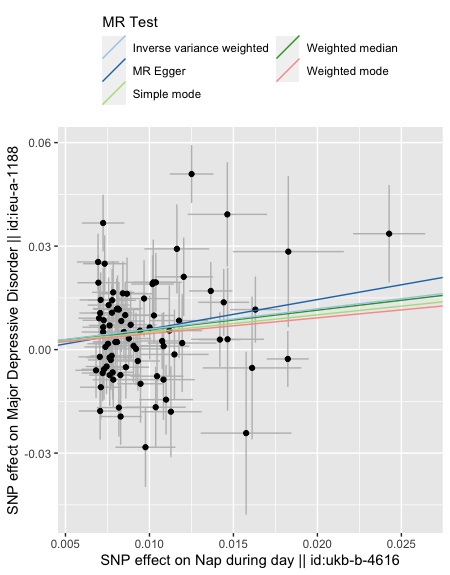


Fig S27. Scatter plot of single-nucleotide polymorphism (SNP) associated with the napping during the day and Major Depressive Disorder.


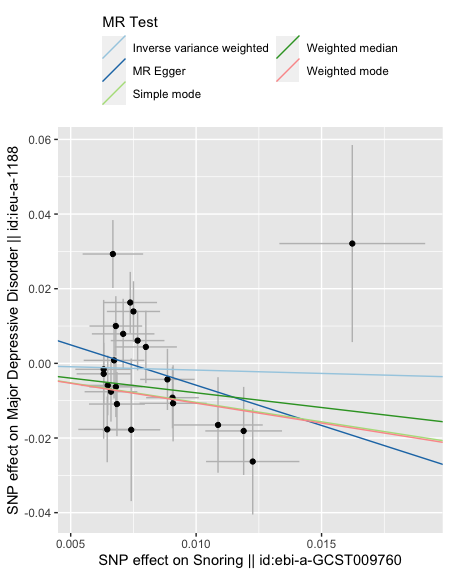


Fig S28. Scatter plot of single-nucleotide polymorphism (SNP) associated with the snoring and Major Depressive Disorder.


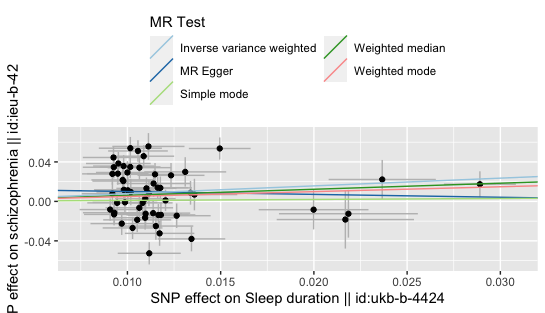


Fig S29. Scatter plot of single-nucleotide polymorphism (SNP) associated with the sleep duration and schizophrenia.


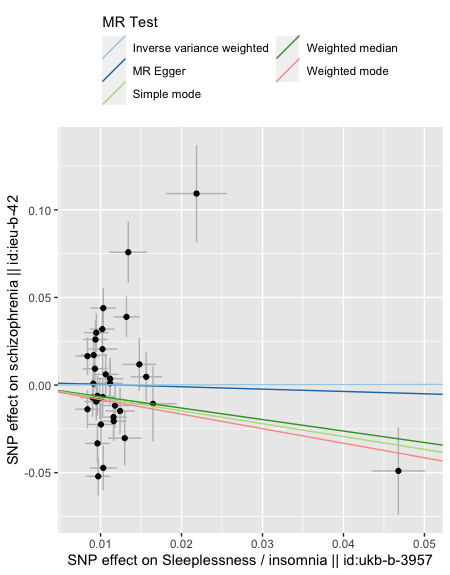


Fig S30. Scatter plot of single-nucleotide polymorphism (SNP) associated with the insomnia and schizophrenia.


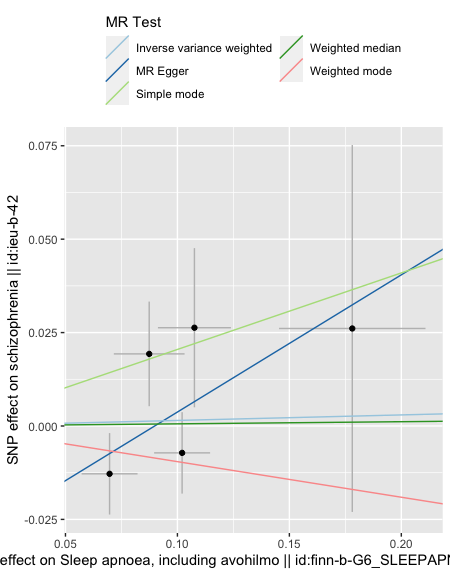


Fig S31. Scatter plot of single-nucleotide polymorphism (SNP) associated with the sleep apnea and schizophrenia.


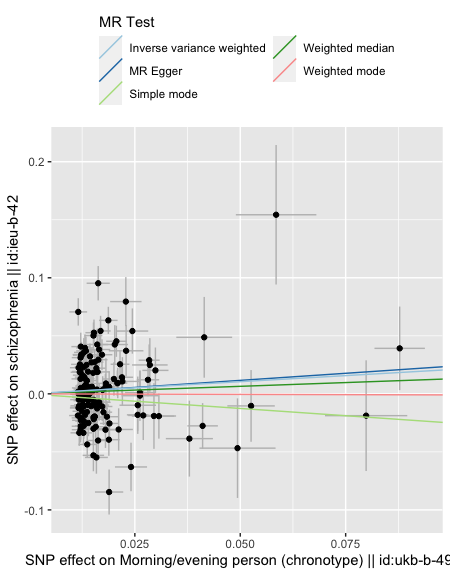


Fig S32. Scatter plot of single-nucleotide polymorphism (SNP) associated with the chronotype and schizophrenia.


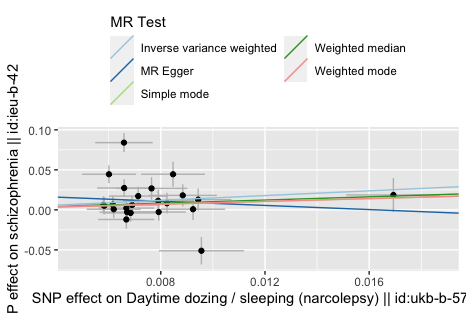


Fig S33. Scatter plot of single-nucleotide polymorphism (SNP) associated with the daytime dozing and schizophrenia.


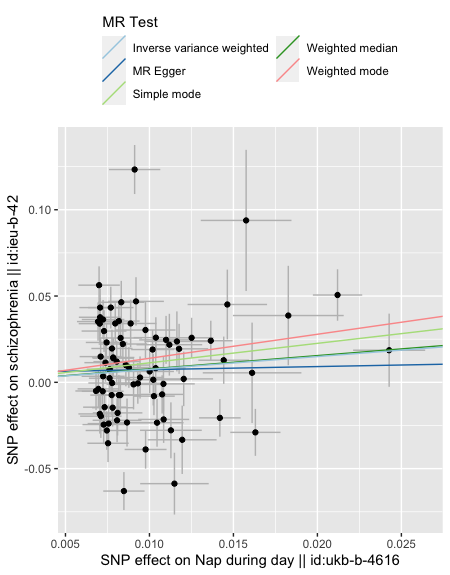


Fig S34. Scatter plot of single-nucleotide polymorphism (SNP) associated with the napping during the day and schizophrenia.


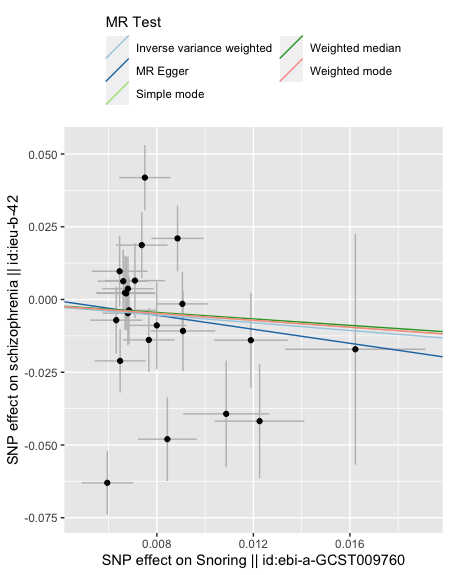


Fig S35. Scatter plot of single-nucleotide polymorphism (SNP) associated with the snoring and schizophrenia.


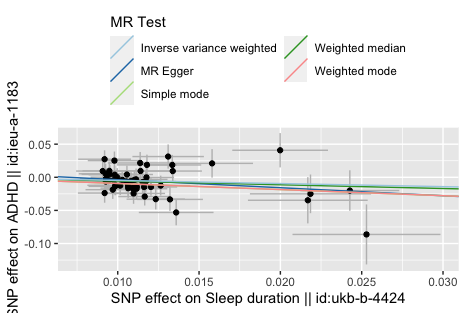


Fig S36. Scatter plot of single-nucleotide polymorphism (SNP) associated with the sleep duration and Attention-Deficit / Hyperactivity Disorder.


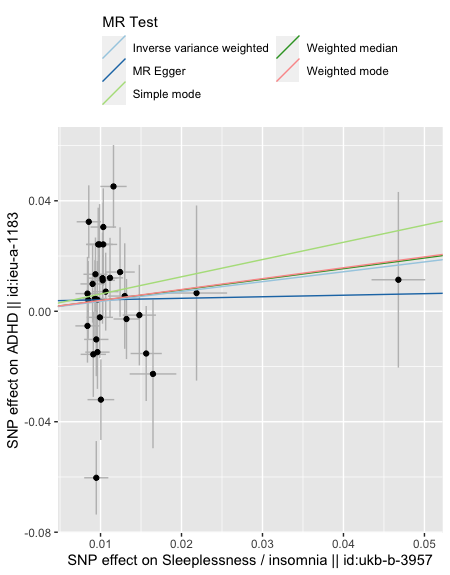


Fig S37. Scatter plot of single-nucleotide polymorphism (SNP) associated with insomnia and Attention-Deficit / Hyperactivity Disorder.


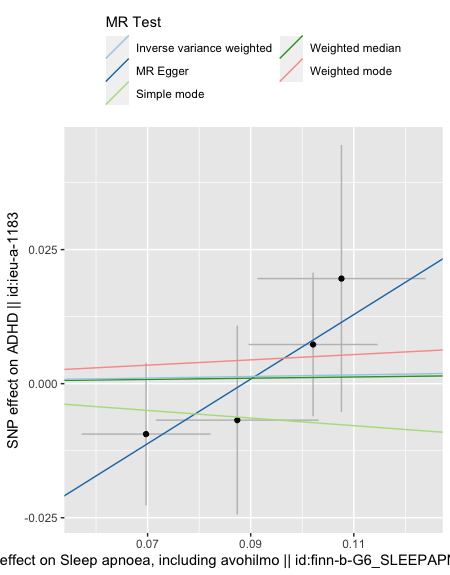


Fig S38. Scatter plot of single-nucleotide polymorphism (SNP) associated with the sleep apnea and Attention-Deficit / Hyperactivity Disorder.


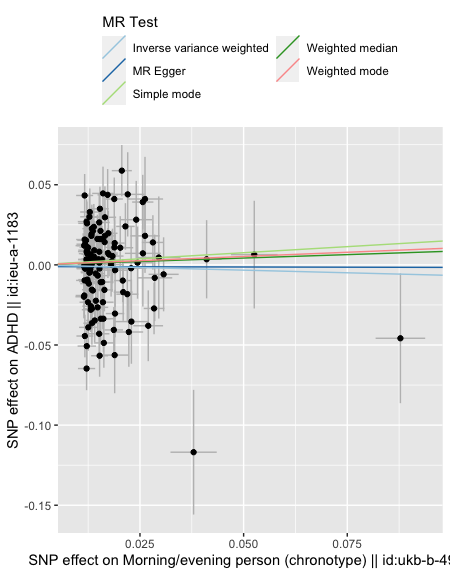


Fig S39. Scatter plot of single-nucleotide polymorphism (SNP) associated with the chronotype and Attention-Deficit / Hyperactivity Disorder.


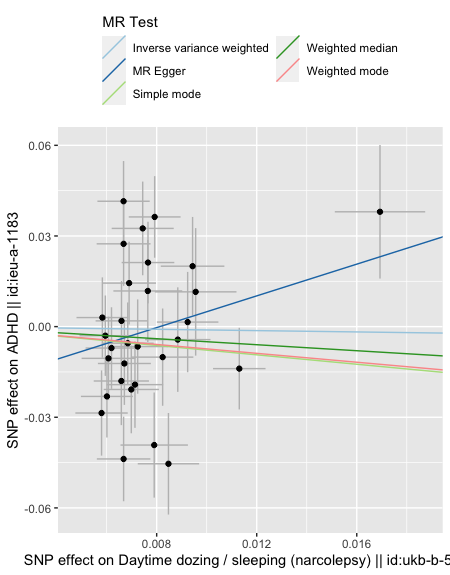


Fig S40. Scatter plot of single-nucleotide polymorphism (SNP) associated with the daytime dozing and Attention-Deficit / Hyperactivity Disorder.


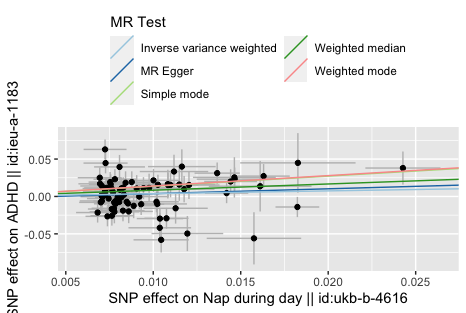


Fig S41. Scatter plot of single-nucleotide polymorphism (SNP) associated with the napping during the day and Attention-Deficit / Hyperactivity Disorder.


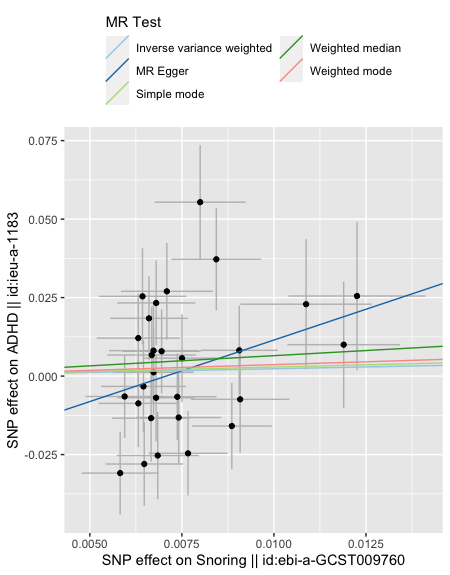


Fig S42. Scatter plot of single-nucleotide polymorphism (SNP) associated with the snoring and Attention-Deficit / Hyperactivity Disorder.


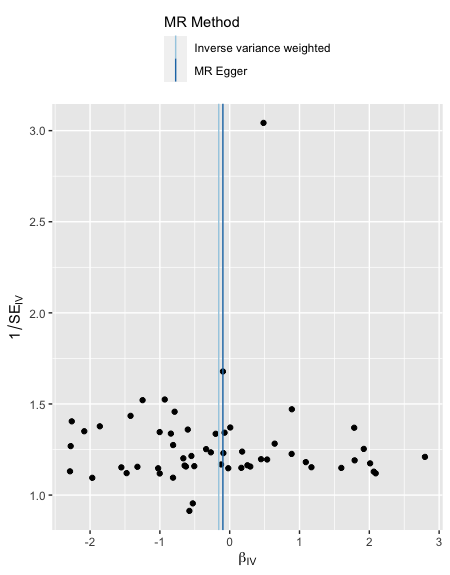


Fig S43. Funnel plot of instrument precision against instrumental variable estimates for each genetic variant separately for Mendelian randomization analysis of sleep duration on Major Depressive Disorder.


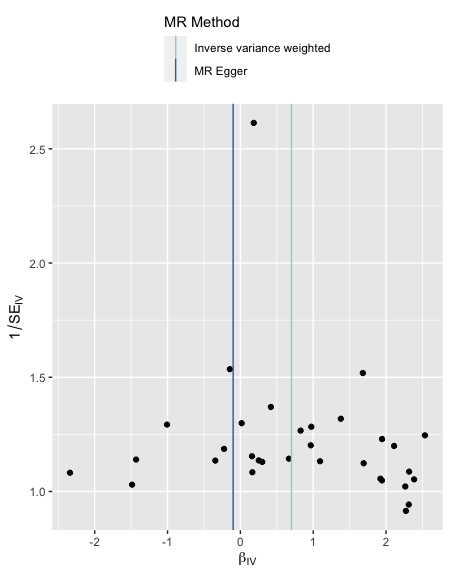


Fig S44. Funnel plot of instrument precision against instrumental variable estimates for each genetic variant separately for Mendelian randomization analysis of insomnia on Major Depressive Disorder.


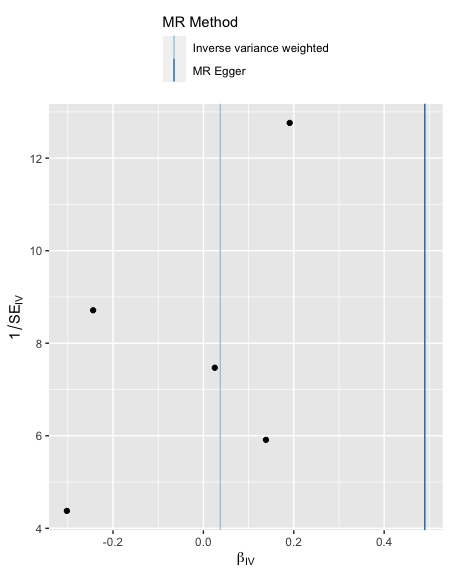


Fig S45. Funnel plot of instrument precision against instrumental variable estimates for each genetic variant separately for Mendelian randomization analysis of sleep apnea on Major Depressive Disorder.


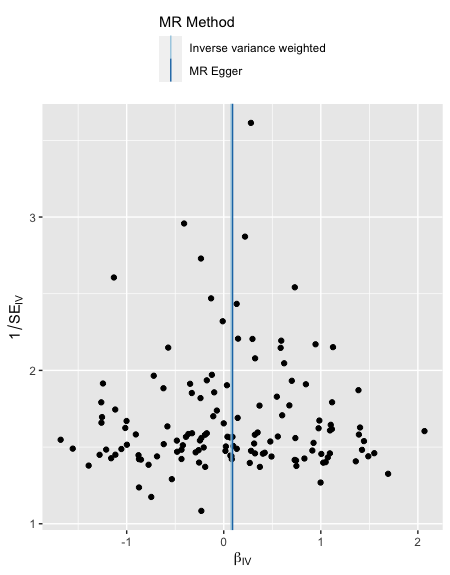


Fig S46. Funnel plot of instrument precision against instrumental variable estimates for each genetic variant separately for Mendelian randomization analysis of chronotype on Major Depressive Disorder.


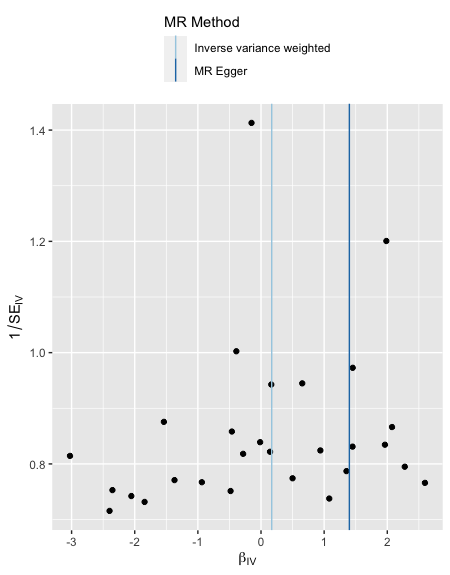


Fig S47. Funnel plot of instrument precision against instrumental variable estimates for each genetic variant separately for Mendelian randomization analysis of daytime dozing on Major Depressive Disorder.


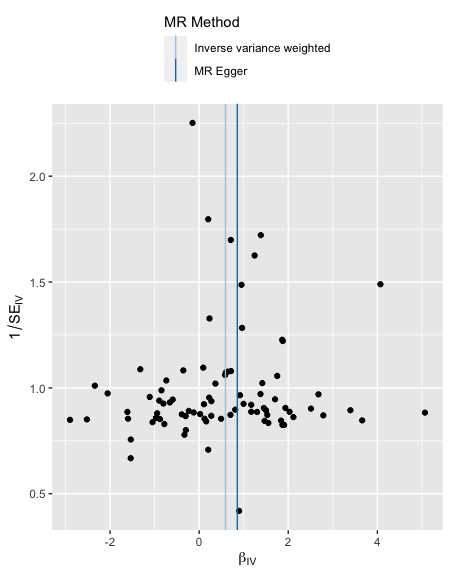


Fig S48. Funnel plot of instrument precision against instrumental variable estimates for each genetic variant separately for Mendelian randomization analysis of napping during the day on Major Depressive Disorder.


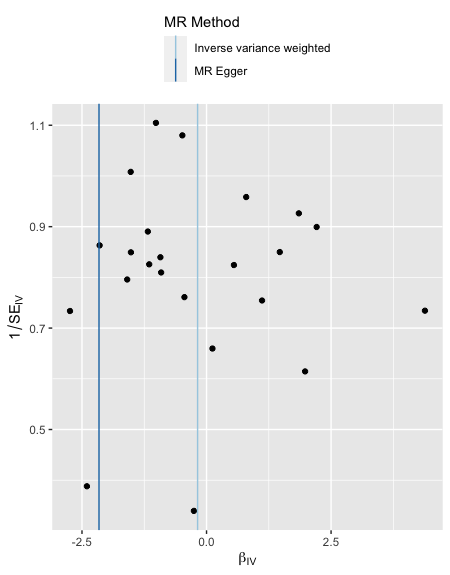


Fig S49. Funnel plot of instrument precision against instrumental variable estimates for each genetic variant separately for Mendelian randomization analysis of snoring on Major Depressive Disorder.


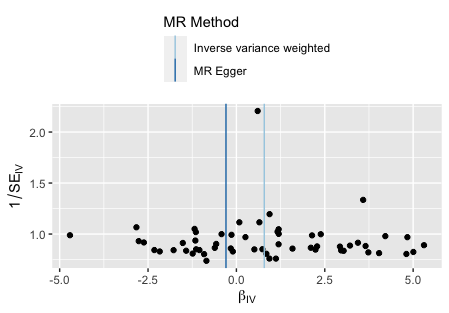


Fig S50. Funnel plot of instrument precision against instrumental variable estimates for each genetic variant separately for Mendelian randomization analysis of sleep duration on schizophrenia.


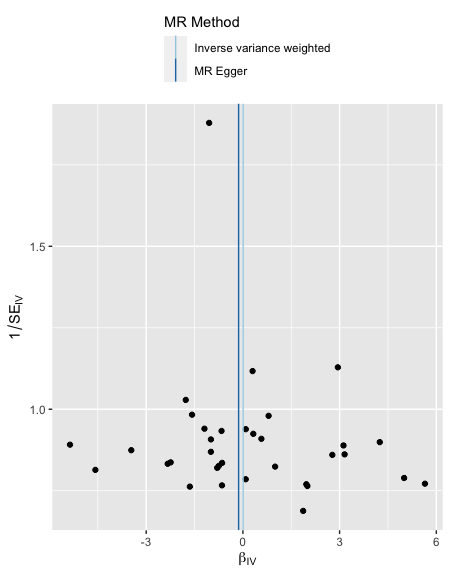


Fig S51. Funnel plot of instrument precision against instrumental variable estimates for each genetic variant separately for Mendelian randomization analysis of insomnia on schizophrenia.


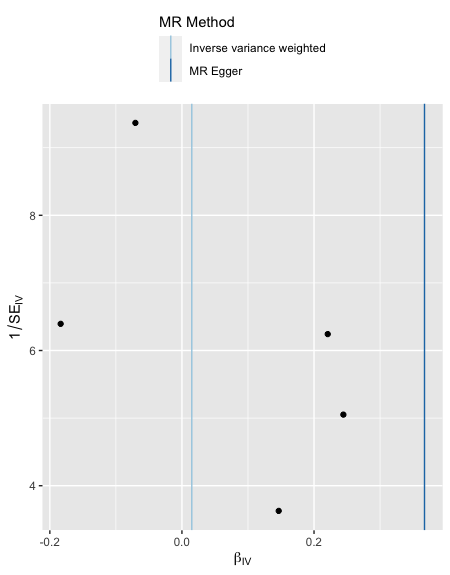


Fig S52. Funnel plot of instrument precision against instrumental variable estimates for each genetic variant separately for Mendelian randomization analysis of sleep apnea on schizophrenia.


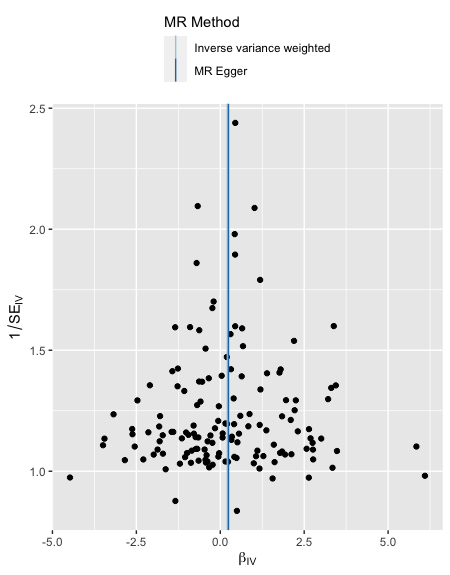


Fig S53. Funnel plot of instrument precision against instrumental variable estimates for each genetic variant separately for Mendelian randomization analysis of chronotype on schizophrenia.


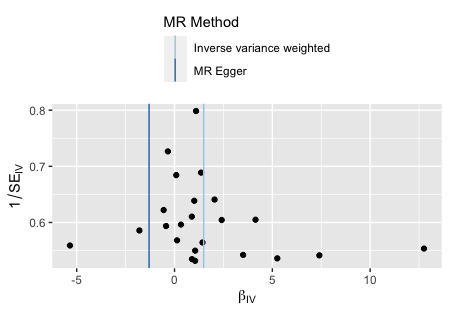


Fig S54. Funnel plot of instrument precision against instrumental variable estimates for each genetic variant separately for Mendelian randomization analysis of daytime dozing on schizophrenia.


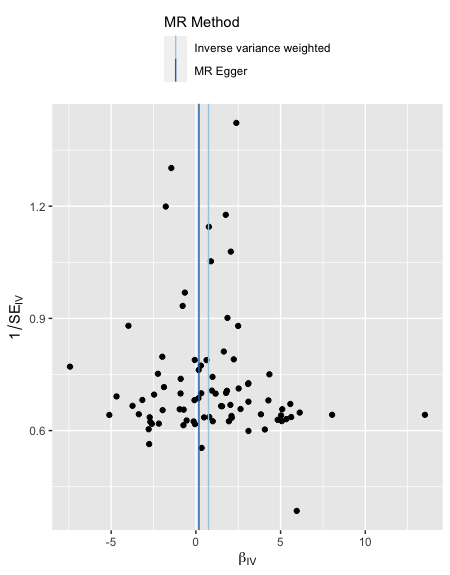


Fig S55. Funnel plot of instrument precision against instrumental variable estimates for each genetic variant separately for Mendelian randomization analysis of napping during the day on schizophrenia.


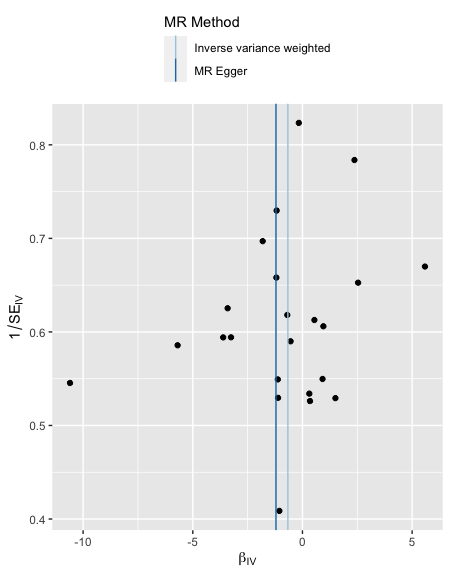


Fig S56. Funnel plot of instrument precision against instrumental variable estimates for each genetic variant separately for Mendelian randomization analysis of snoring on schizophrenia.


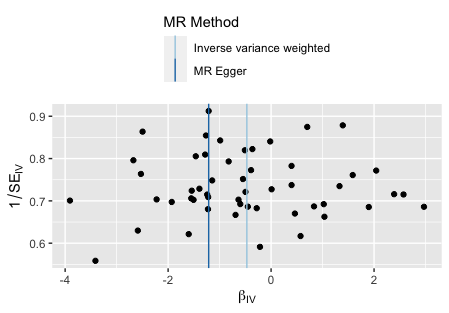


Fig S57. Funnel plot of instrument precision against instrumental variable estimates for each genetic variant separately for Mendelian randomization analysis of sleep duration on Attention-Deficit / Hyperactivity Disorder.


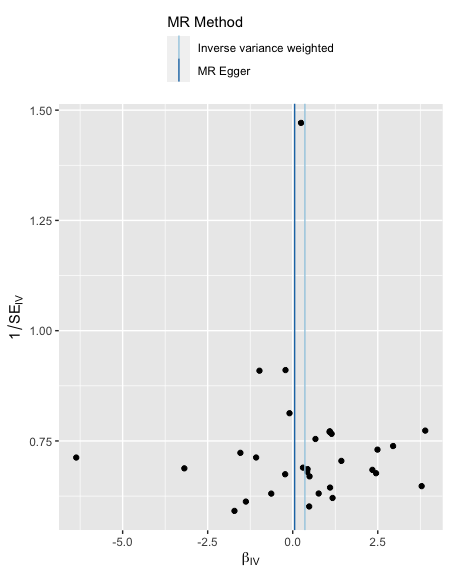


Fig S58. Funnel plot of instrument precision against instrumental variable estimates for each genetic variant separately for Mendelian randomization analysis of insomnia on Attention-Deficit / Hyperactivity Disorder.


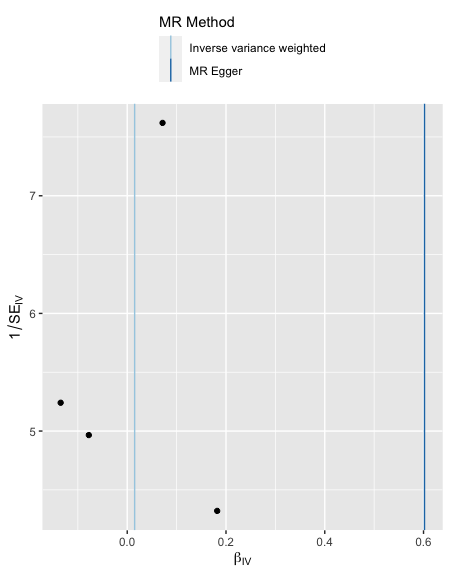


Fig S59. Funnel plot of instrument precision against instrumental variable estimates for each genetic variant separately for Mendelian randomization analysis of sleep apnea on Attention-Deficit / Hyperactivity Disorder.


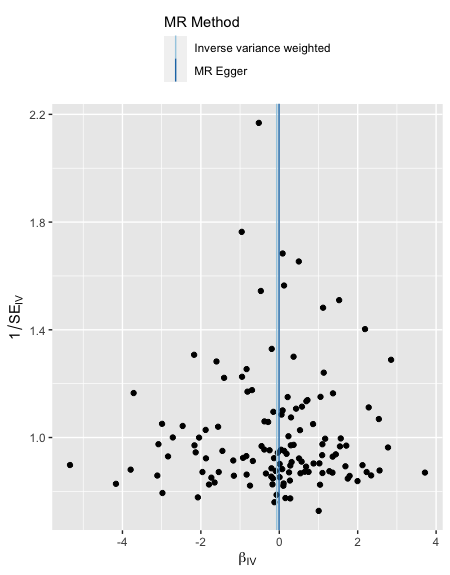


Fig S60. Funnel plot of instrument precision against instrumental variable estimates for each genetic variant separately for Mendelian randomization analysis of chronotype on Attention-Deficit / Hyperactivity Disorder.


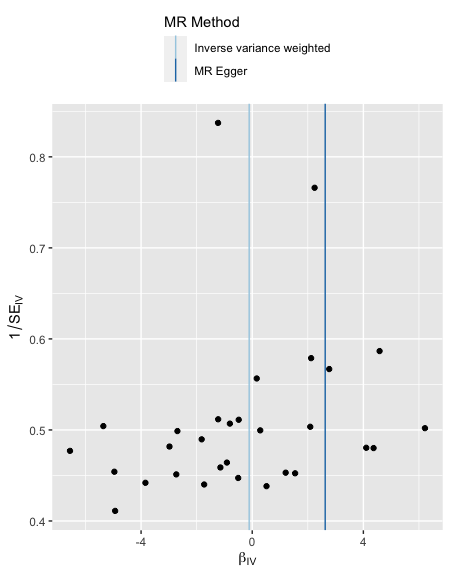


Fig S61. Funnel plot of instrument precision against instrumental variable estimates for each genetic variant separately for Mendelian randomization analysis of daytime dozing on Attention-Deficit / Hyperactivity Disorder.


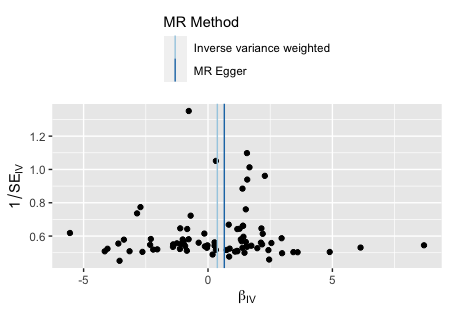


Fig S62. Funnel plot of instrument precision against instrumental variable estimates for each genetic variant separately for Mendelian randomization analysis of napping during the day on Attention-Deficit / Hyperactivity Disorder.


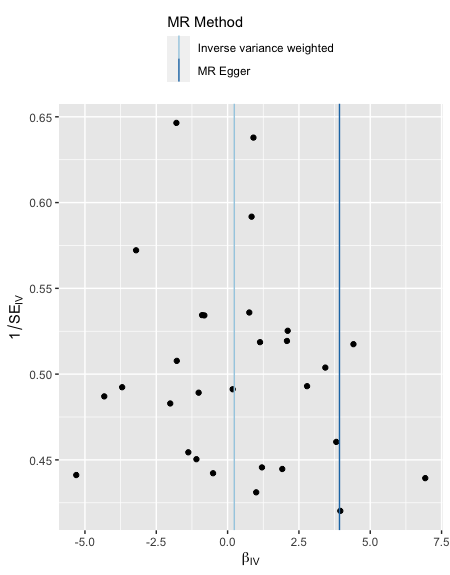


Fig S63. Funnel plot of instrument precision against instrumental variable estimates for each genetic variant separately for Mendelian randomization analysis of snoring on Attention-Deficit / Hyperactivity Disorder.


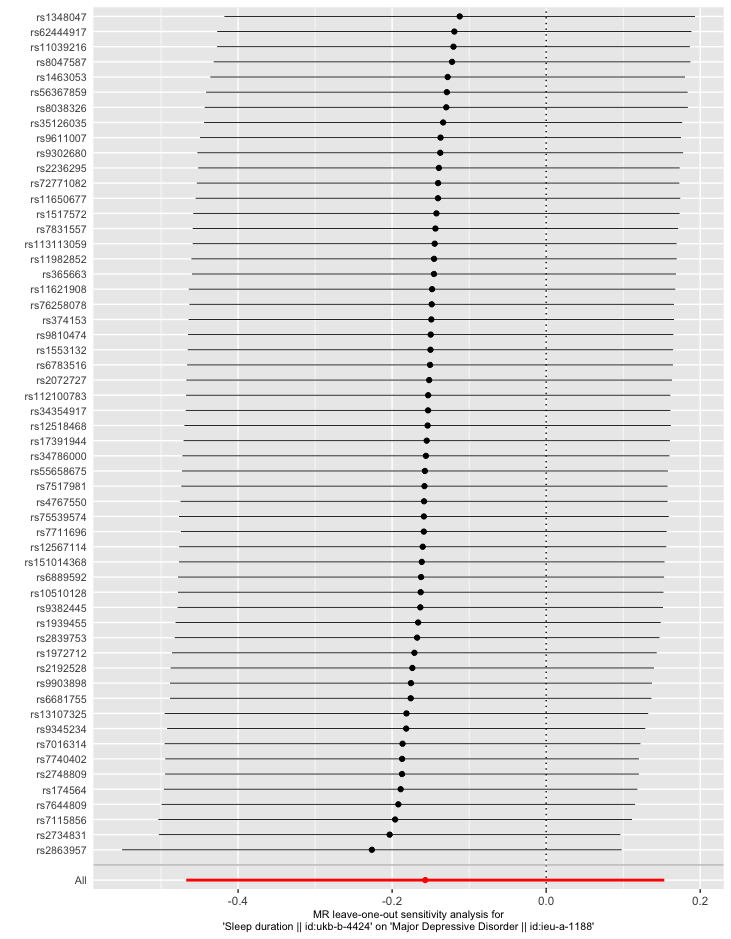
Fig S64. Leave-one-out analysis of association between genetically predicted sleep duration on Major Depressive Disorder.


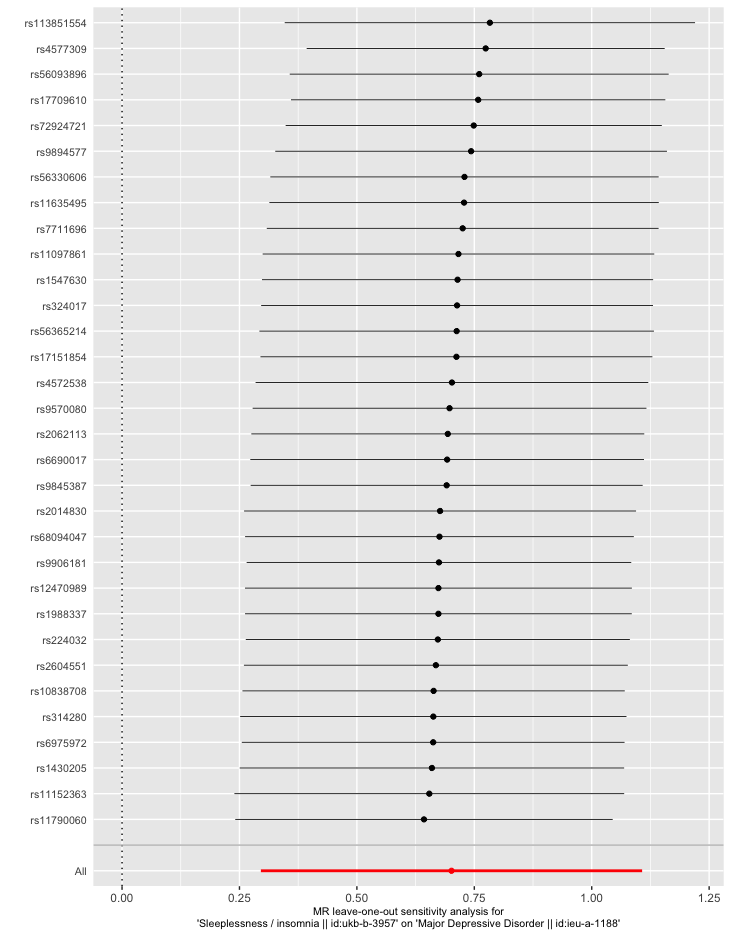
Fig S65. Leave-one-out analysis of association between genetically predicted insomnia on Major Depressive Disorder.


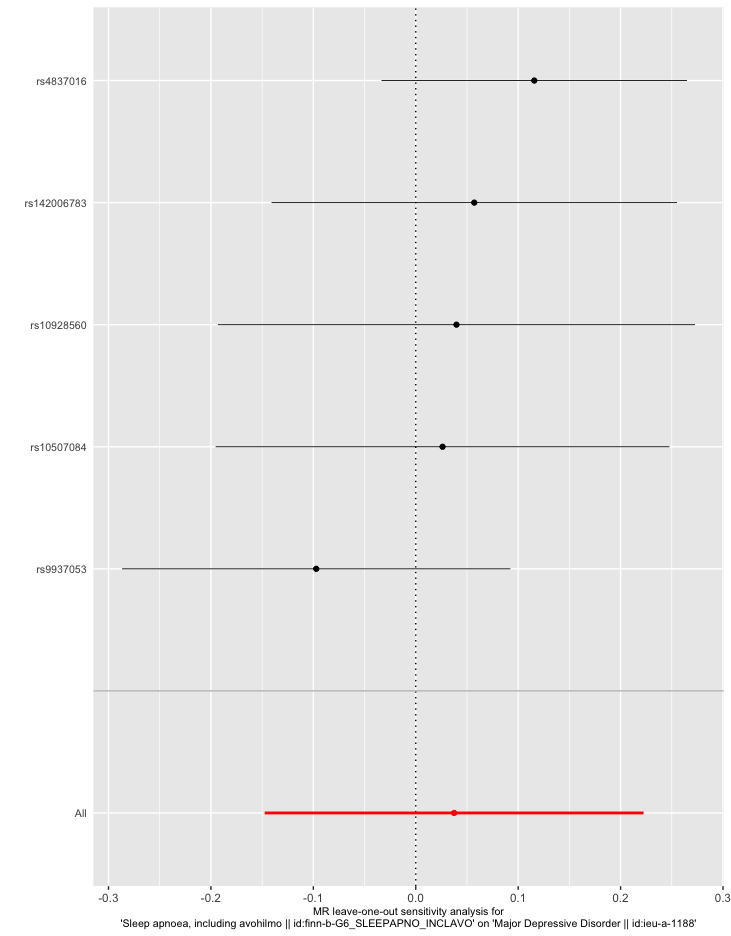
Fig S67. Leave-one-out analysis of association between genetically predicted sleep apnea on Major Depressive Disorder.


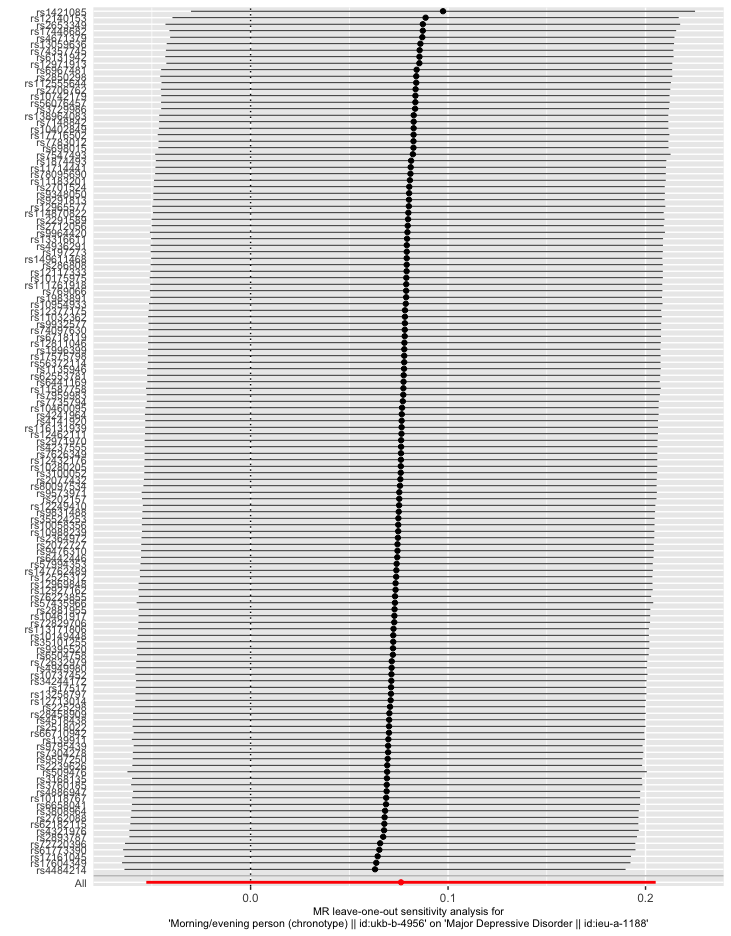
Fig S67. Leave-one-out analysis of association between genetically predicted chronotype on Major Depressive Disorder.


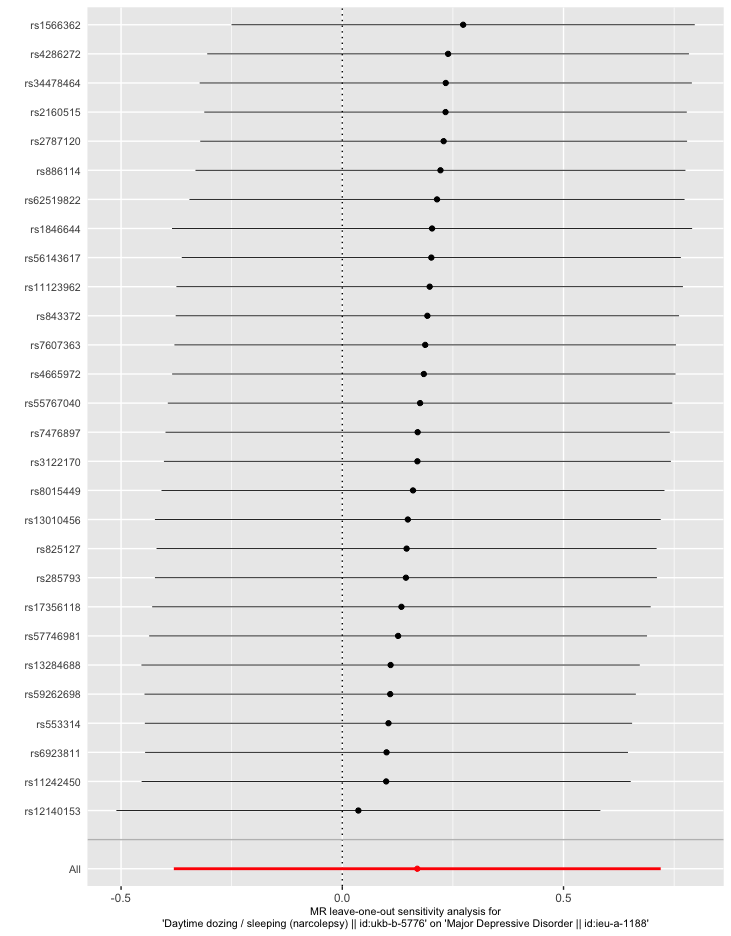
Fig S68. Leave-one-out analysis of association between genetically predicted daytime dozing on Major Depressive Disorder.


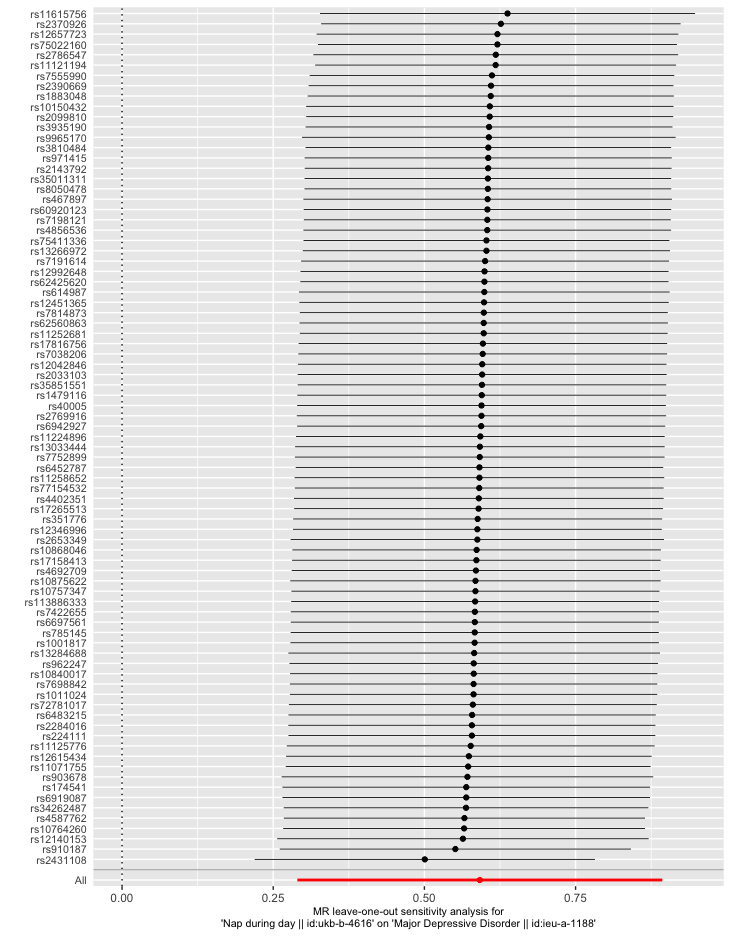
Fig S69. Leave-one-out analysis of association between genetically predicted napping during day on Major Depressive Disorder.


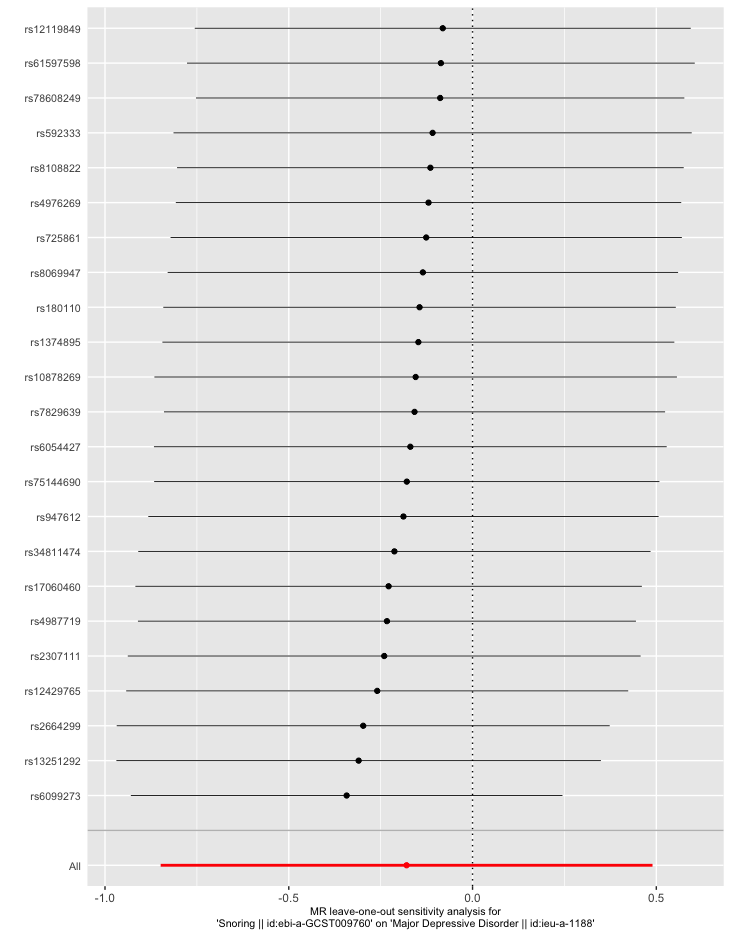
Fig S70. Leave-one-out analysis of association between genetically predicted snoring on Major Depressive Disorder.


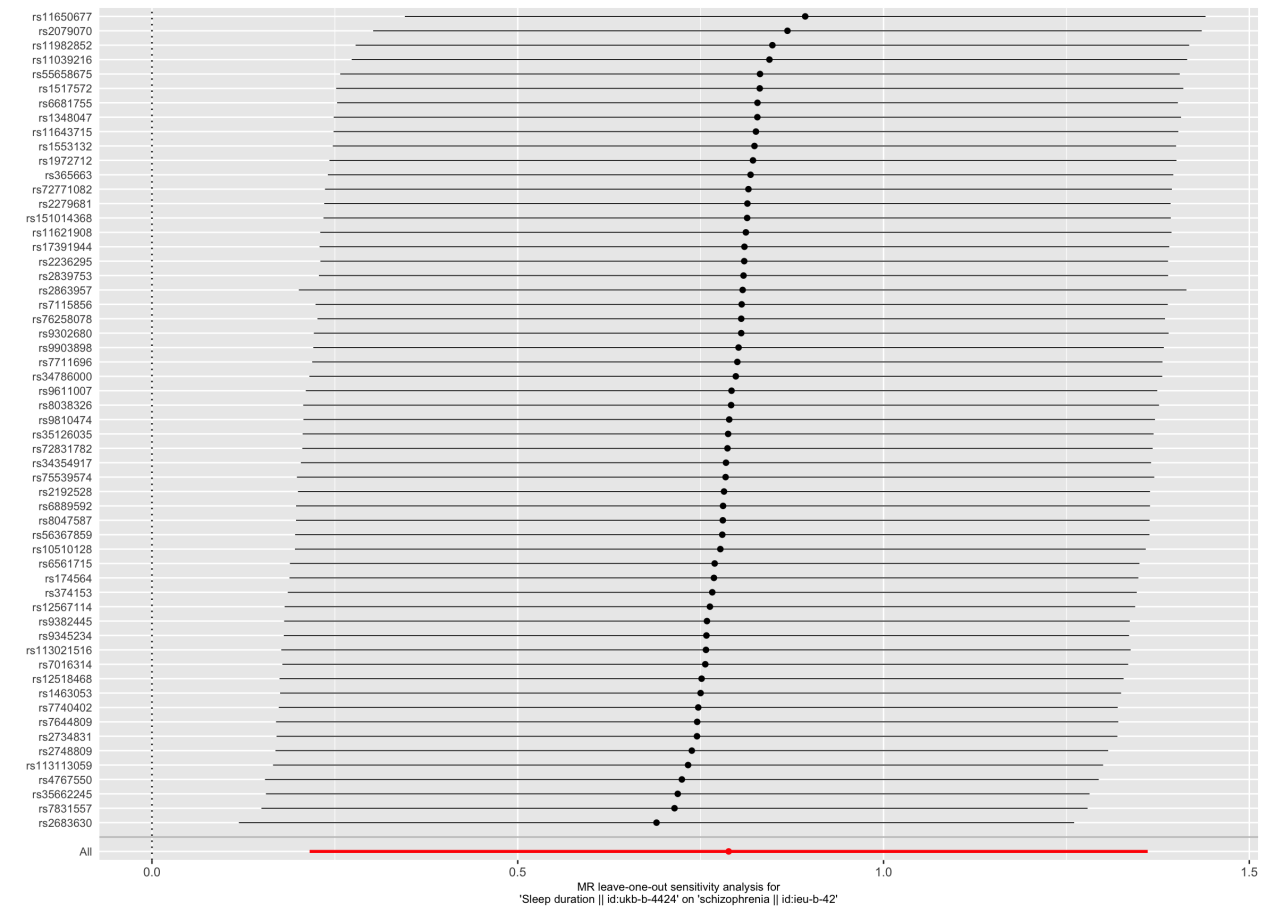
Fig S71. Leave-one-out analysis of association between genetically predicted sleep duration on schizophrenia.


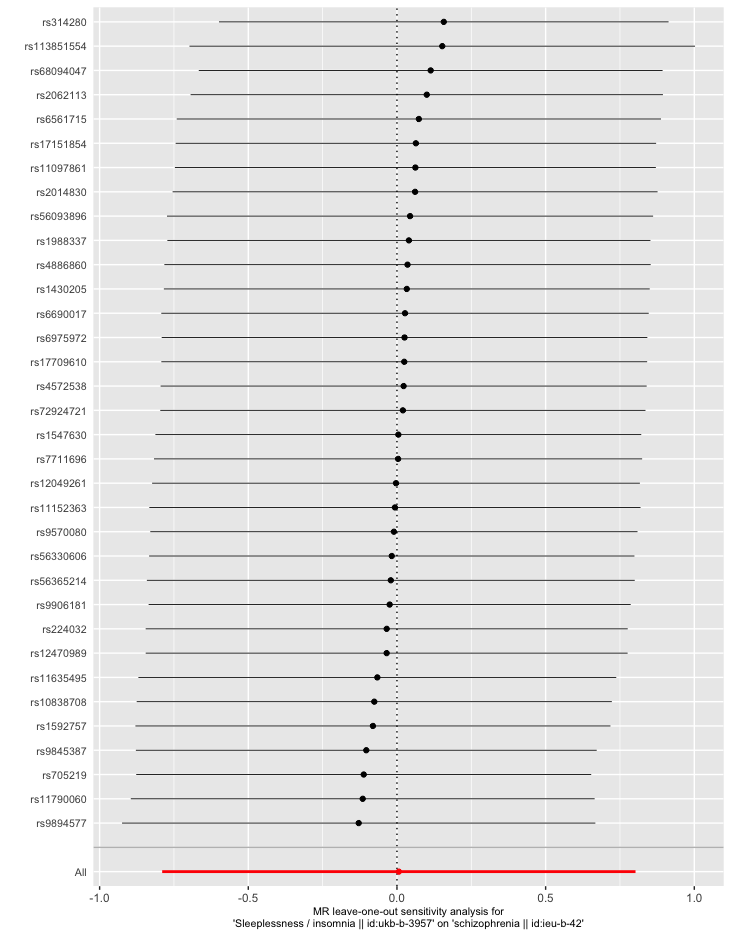


Fig S72. Leave-one-out analysis of association between genetically predicted insomnia on schizophrenia.


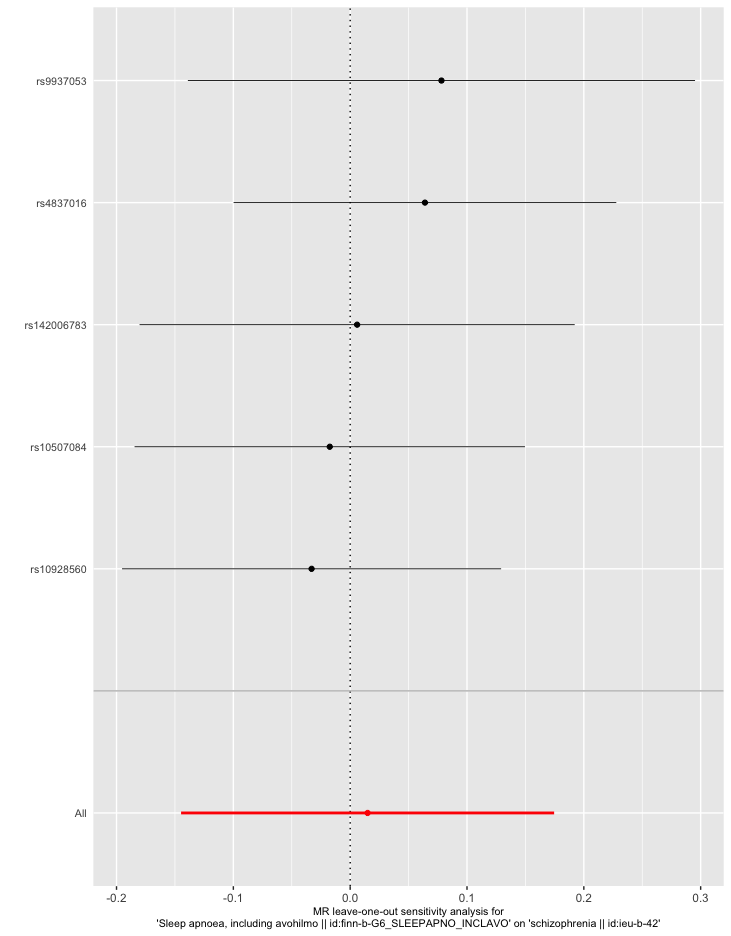
Fig S73. Leave-one-out analysis of association between genetically predicted sleep apnea on schizophrenia.


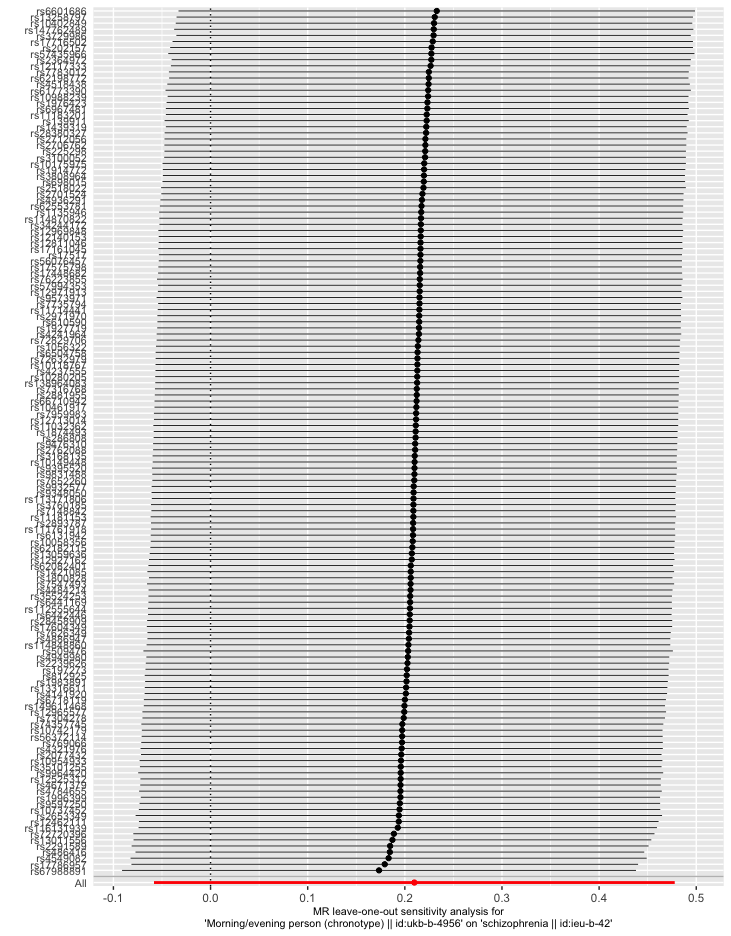
Fig S74. Leave-one-out analysis of association between genetically predicted chronotype on schizophrenia.


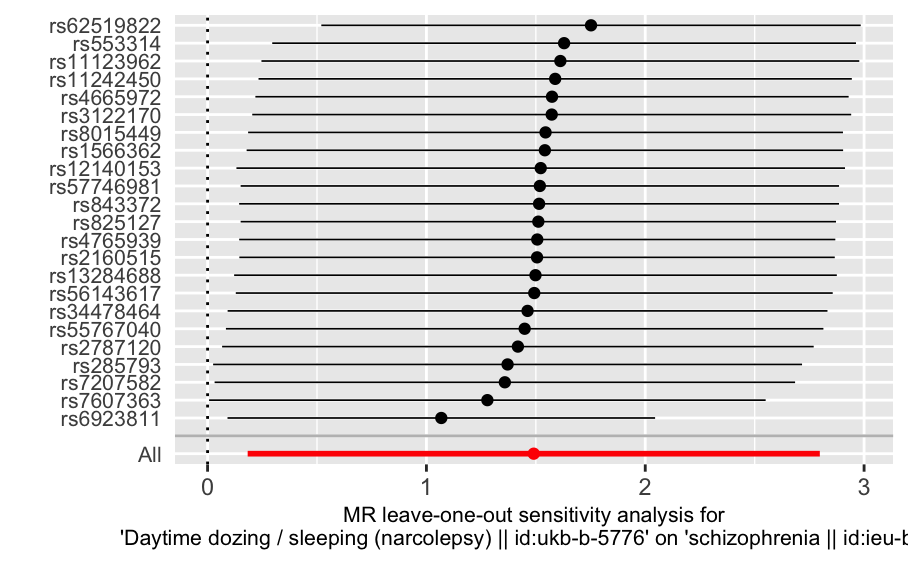


Fig S75. Leave-one-out analysis of association between genetically predicted daytime dozing on schizophrenia.


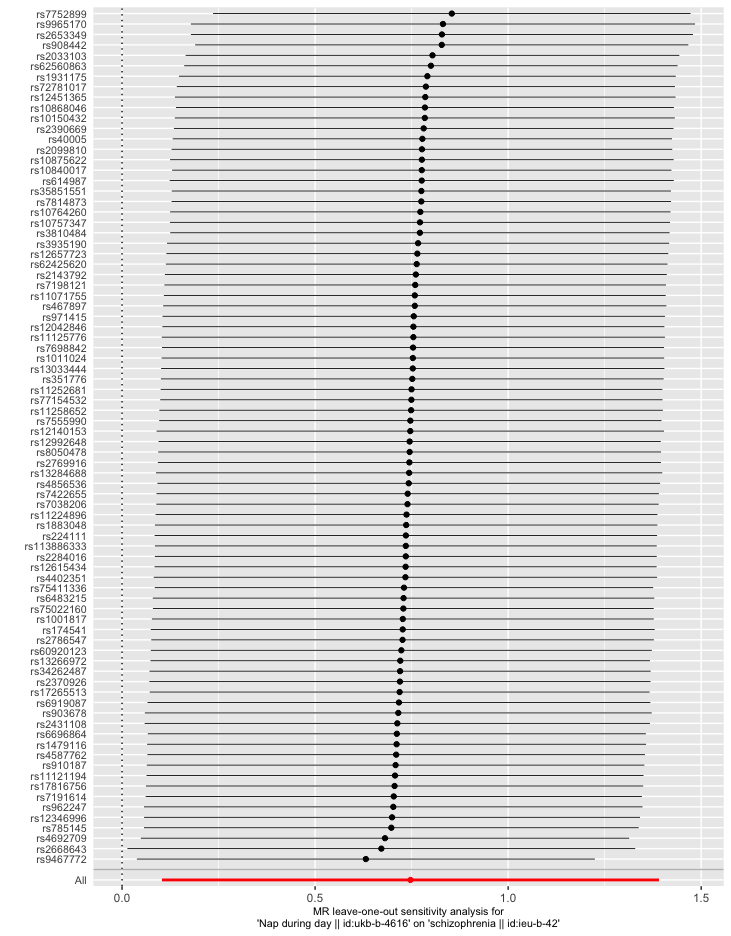
Fig S76. Leave-one-out analysis of association between genetically predicted napping during the day on schizophrenia.


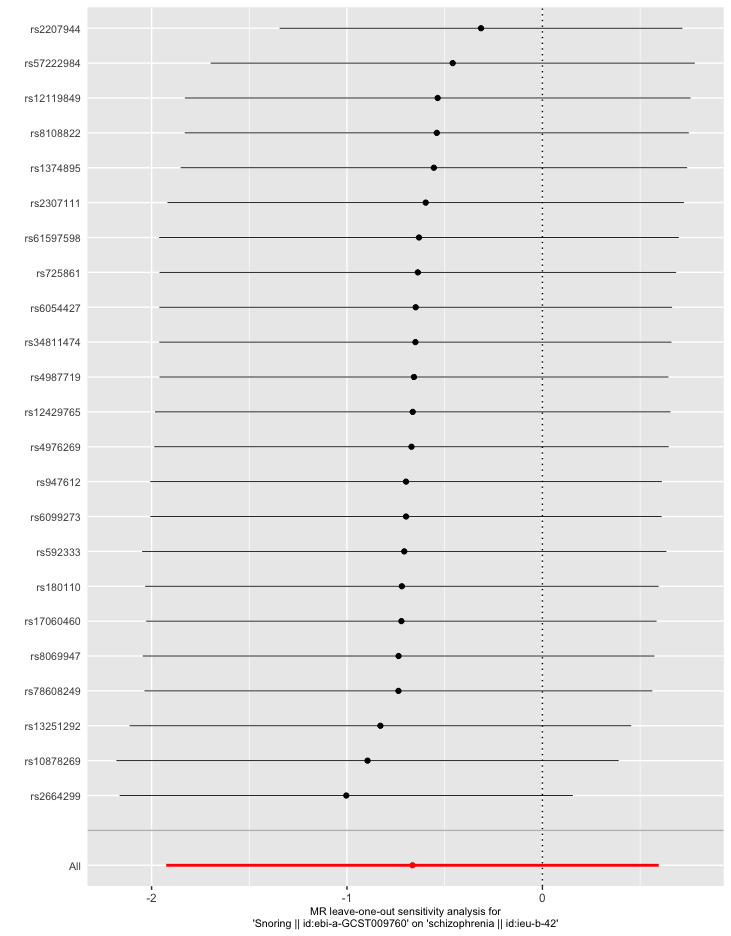
Fig S77. Leave-one-out analysis of association between genetically predicted snoring on schizophrenia.


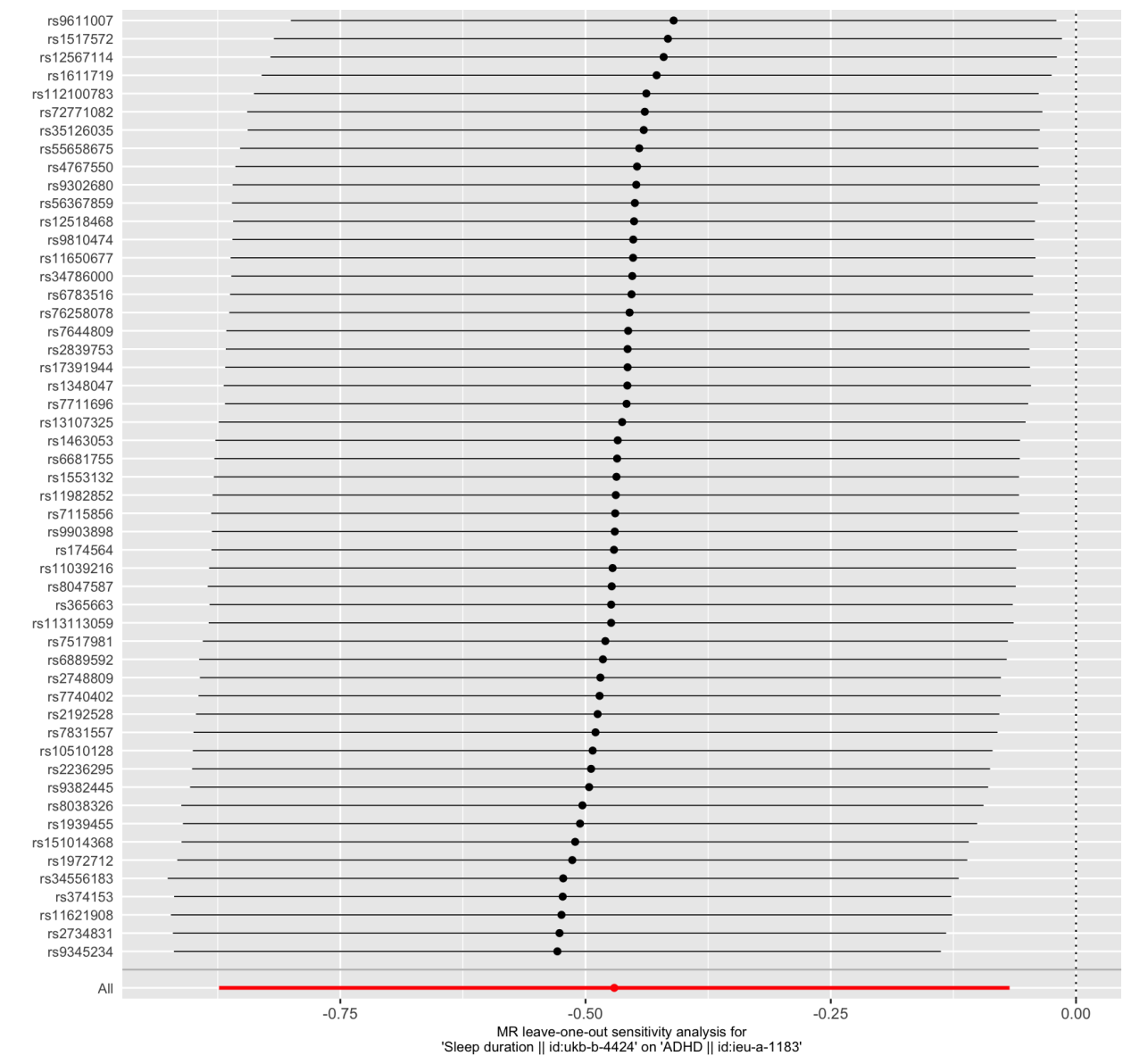
Fig S78. Leave-one-out analysis of association between genetically predicted sleep duration on Attention-Deficit / Hyperactivity Disorder.


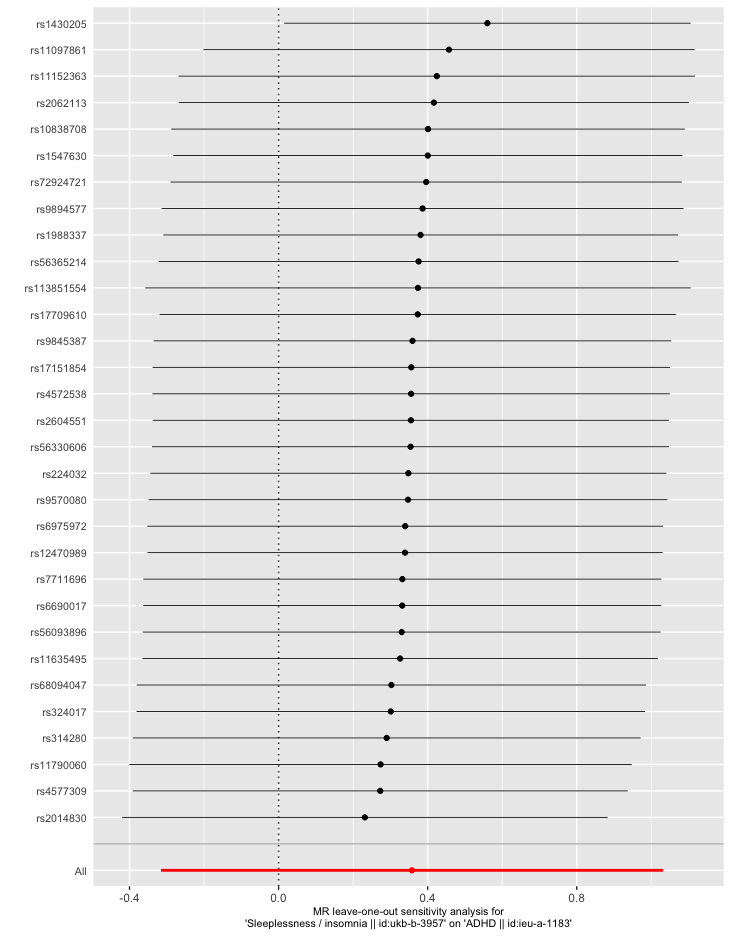
Fig S79. Leave-one-out analysis of association between genetically predicted insomnia duration on Attention-Deficit / Hyperactivity Disorder.


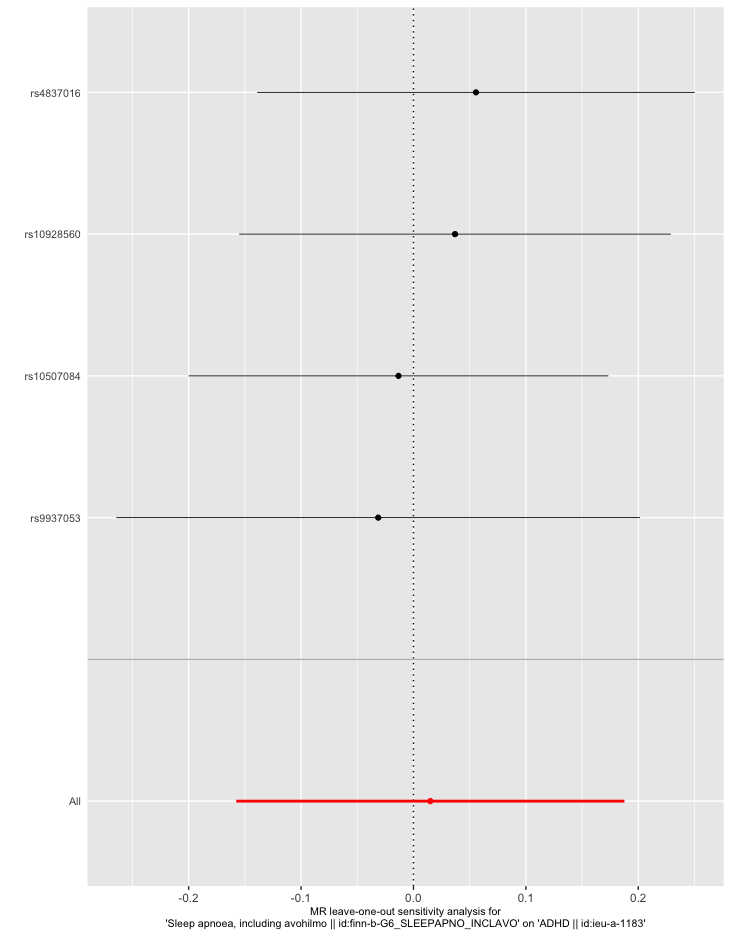
Fig S80. Leave-one-out analysis of association between genetically predicted sleep apnea on Attention-Deficit / Hyperactivity Disorder.


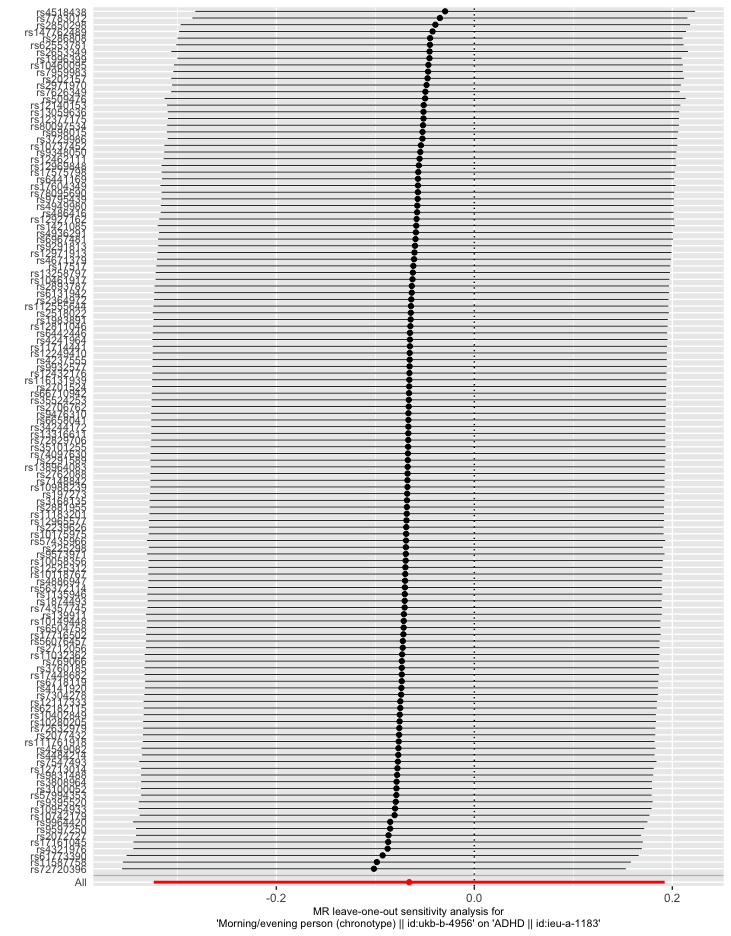
Fig S81. Leave-one-out analysis of association between genetically predicted chronotype on Attention-Deficit / Hyperactivity Disorder.


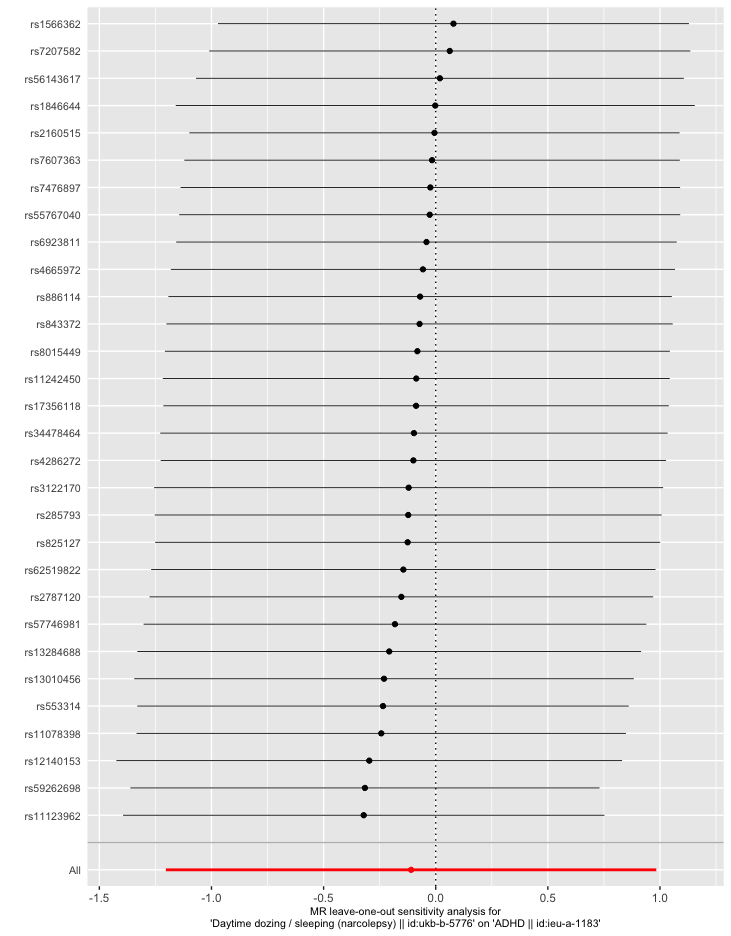
Fig S82. Leave-one-out analysis of association between genetically predicted daytime dozing on Attention-Deficit / Hyperactivity Disorder.


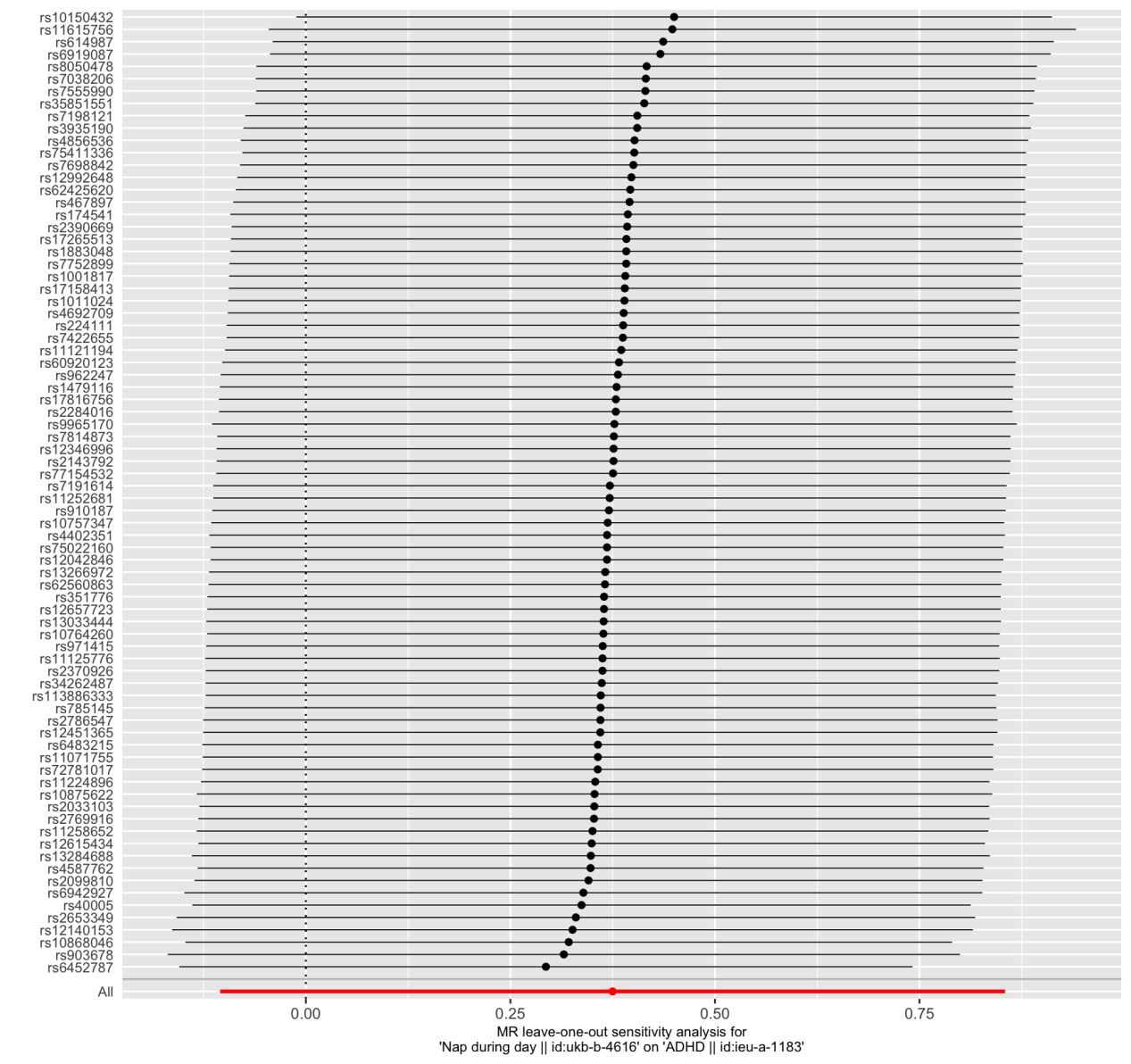
Fig S83. Leave-one-out analysis of association between genetically predicted napping during the day on Attention-Deficit / Hyperactivity Disorder.


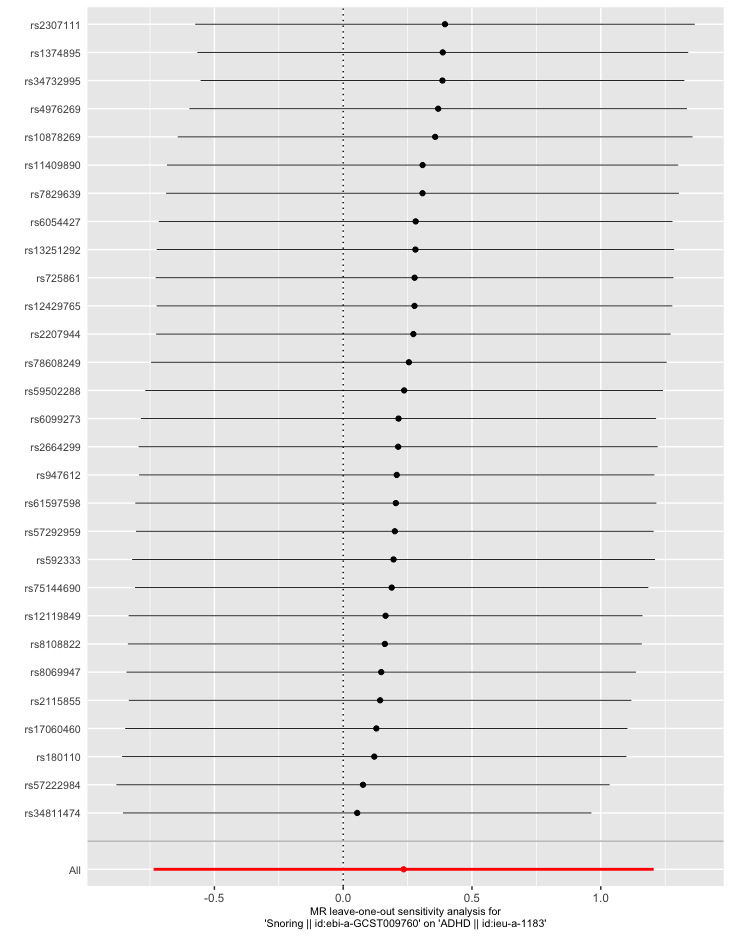
Fig S84. Leave-one-out analysis of association between genetically predicted snoring on Attention-Deficit / Hyperactivity Disorder.
